# Oxidant‐Free Amidation of Aldehydes Enabled by Electrophotocatalysis

**DOI:** 10.1002/chem.202502237

**Published:** 2025-08-11

**Authors:** Dimitris I. Ioannou, Elena Bombonato, Jiri Sanramat, Joost N. H. Reek, Timothy Noël

**Affiliations:** ^1^ Flow Chemistry Group van ’t Hoff Institute for Molecular Sciences (HIMS) University of Amsterdam Science Park 904 Amsterdam 1098 XH The Netherlands; ^2^ Supramolecular and Homogeneous Catalysis Group van ’t Hoff Institute for Molecular Sciences (HIMS) University of Amsterdam Science Park 904 Amsterdam 1098 XH The Netherlands; ^3^ Department of Chemistry “Giacomo Ciamician” Università di Bologna Via Selmi, 2 Bologna 40126 Italy

**Keywords:** electrophotocatalysis, hydrogen atom transfer, iron‐catalyzed, oxidant‐free, radical‐polar crossover

## Abstract

Electrophotocatalysis (EPC) is emerging as a powerful tool in organic synthesis, offering unique redox transformations without the need for sacrificial oxidants or reductants. Building on this activation mode, we have developed an electrophotocatalytic method for the direct amidation of aldehydes, utilizing electricity and protons as oxidants and light to generate chlorine radicals for hydrogen atom transfer (HAT). This sustainable approach is compatible with a wide range of aldehydes and nitrogen‐based nucleophiles, performing efficiently under batch conditions and demonstrating scalability with flow technology. The mild reaction conditions, adequate functional group tolerance, and versatility make this EPC protocol particularly suitable for C─N bond formation, enabling functionalization of diverse organic compounds and facilitating late‐stage modifications in drug development.

## Graphical abstract

An electrophotocatalytic method for C(sp^2^)–N bond formation via radical‐polar crossover (RPC)‐HAT catalysis is presented. The mild operating conditions enable functionalization of a wide range of compounds, with electricity as the sole oxidant.

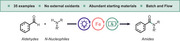

## 1 Introduction

Over the past 15 years, photocatalysis and electrocatalysis have transformed the landscape of organic synthesis, introducing innovative approaches that have expanded the synthetic repertoire of chemists.^[^ ^1^ ^]^ Photocatalysis harnesses light energy to generate open‐shell reaction intermediates under mild conditions, enabling the formation of reactive radicals that drive challenging molecular transformations.^[^ ^2^ ^]^ Electrocatalysis, on the other hand, uses electrical energy to initiate redox reactions, offering a broad redox window that enhances control over reaction conditions, thereby improving efficiency, selectivity, and sustainability.^[^ ^3^ ^]^ The integration of these two methodologies has led to the emergence of electrophotocatalysis (EPC), a hybrid strategy that combines light and electrical energy to unlock unique chemical transformations while eliminating the need for sacrificial oxidants and reductants. This rapidly growing field has opened new avenues for the development of more efficient, selective, and environmentally sustainable synthetic methodologies.^[^ ^4^ ^]^

Among the catalysts used in EPC, FeCl_3_ has emerged as particularly valuable due to its unique properties and versatility. In synthetic chemistry, FeCl_3_ is well‐known for its ability to generate chlorine radicals through photoinduced ligand‐to‐metal charge transfer (LMCT).^[^ ^5^ ^]^

Unlike nickel‐based catalysts, which can be prone to instability, Fe(III)–Cl complexes are highly stable and can efficiently release chlorine radicals upon exposure to light.^[^ ^6^ ^]^

**Scheme 1:**
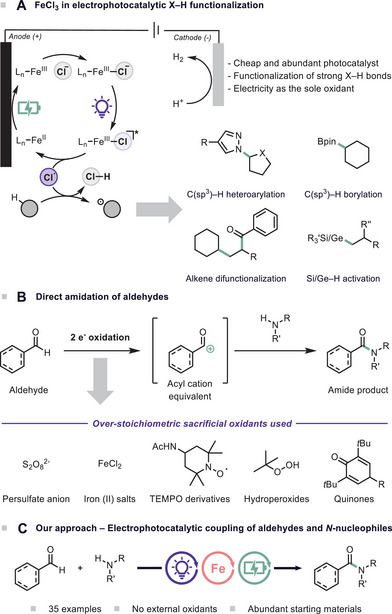
A) State of the art employing FeCl_3_ in EPC. B) Traditional approaches for the direct amidation of aldehydes. C) Electrophotocatalytic C(sp^2^)‒H coupling of aldehydes and *N*‐nucleophiles.

These radicals are highly effective in driving hydrogen atom transfer (HAT) processes, enabling efficient and selective functionalization of C─H bonds.^[^ ^7^ ^]^ By leveraging Fe(III)─Cl catalysts, EPC offers a sustainable and cost‐effective approach to synthesizing a broad range of valuable compounds, including C(sp^3^)─H heteroarylation,^[^ ^8^ ^]^ C(sp^3^)─H borylation,^[^ ^9^ ^]^ alkene difunctionalization,^[^ ^10^ ^]^ and Si/Ge─H activation,^[^ ^10a^ ^]^ unlocking new opportunities in synthetic chemistry (Scheme 1A).

Amide bonds are among the most ubiquitous functional groups found in nature, especially in biomolecules such as proteins, as well as in numerous pharmaceuticals and agrochemicals.^[^ ^11^ ^]^ Traditionally, amide synthesis involves the coupling between carboxylic acids and amines, often requiring the use of an excess of activating agents to drive the reaction forward.^[^ ^12^ ^]^ In recent years, an alternative method known as aldehyde oxidative amidation has emerged as a promising approach for the formation of amides. This method displays favorable atom economy and selectivity by using aldehydes as starting materials.^[^ ^13^ ^]^ However, it requires the use of an excess of sacrificial oxidants to promote the reaction. Common oxidants employed in this process include persulfate anions,^[^ ^14^ ^]^ transition metal salts,^[^ ^15^ ^]^ organoradical derivatives,^[^ ^16^ ^]^ hydroperoxides,^[^ ^17^ ^]^ and quinones.^[^ ^18^ ^]^ Despite their effectiveness, these oxidants can be costly and generate by‐products and waste, thus limiting the sustainability of the process (Scheme 1B).

Based on our previous work on HAT processes,^[^ ^8^, ^19^ ^]^ we hypothesized that the direct activation of aldehydes using an inexpensive FeCl_3_‐based HAT catalyst could provide a more efficient and scalable solution. By leveraging the capabilities of electrophotocatalytic activation, we envisioned a method to replace stoichiometric oxidants with an electrochemical process, minimizing waste while maintaining high selectivity and efficiency. This reaction blueprint offers a robust and sustainable strategy for oxidative amidation, addressing the lingering challenges associated with traditional methods and enhancing the overall utility of amide bond formation in synthetic chemistry (Scheme 1C).

## 2 Results and Discussion

Our initial investigations commenced with the direct azolation of aromatic aldehydes as a benchmark transformation to showcase our hypothesis (Table 1). The reactions were conducted in a batch EPC reactor (Supporting Information, Figure S1) using an acetonitrile solution containing 4‐chlorobenzaldehyde **1a** (0.15 M), pyrazole **2a** (0.1 M), and FeCl_3_ (15 mol%), in the presence of acetic acid (10 equiv) and Et_4_NCl (1.5 equiv). The reaction was irradiated with purple LEDs (λ = 390 nm, 52 W input power) and operated in galvanostatic mode (1.5 mA, 0.75 mA·cm^−2^, 4.5 F·mol^−1^). Product **3** was isolated in an excellent yield (75%) after a 16‐hour reaction time (Table 1, Entry 1). As expected, product formation was negligible in the absence of electricity or light, highlighting the electrophotocatalytic nature of this transformation (Table 1, Entries 2–3). The removal of the iron catalyst, or its use in a stoichiometric amount but without current, triggered undesirable side reactions and resulted in decreased yields (Table 1, Entries 4–5). Reversing the stoichiometry of the starting materials, using **1a** as the limiting reagent, resulted in comparable yields (Table 1, Entry 6), offering flexible reaction conditions that enable the selection of the limiting reagent based on the cost or availability of the coupling partners. Increasing the applied current to 3 mA (1.5 mA·cm^−2^), to reduce reaction times, did not furnish better results (Table 1, Entry 7). Similarly, reducing the electron equivalents to 2.5 F·mol^−1^, did not lead to full conversion of the starting material (Table 1, Entry 8). To further investigate alternative reaction conditions, we transitioned to a basic environment using 2,4,6‐collidine in CH_2_Cl_2_^[^ ^20^ ^]^; however, these conditions proved not compatible with the desired transformation (Table 1, Entry 9).

**Table 1:** Optimization of reaction conditions and control experiments for the electrophotocatalytic C(sp^2^)‒H amidation.^[^ ^a^ ^]^

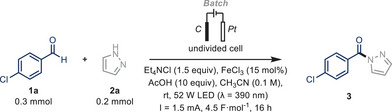		
Entry	Variation from conditions	Yield^[^ ^b^ ^]^
1	None	92 (75)
2	Without electricity	8
3	Without light irradiation	–
4	Without FeCl_3_	34
5	FeCl_3_ (100 mol%) – without electricity	50
6	**1a** 0.2 mmol, **2a** 0.6 mmol	90 (74)
7	3 mA, 4.5 F·mol^−1^, 8 hours	66
8	1.5 mA, 2.5 F·mol^−1^, 9 hours	48
9	2,4,6‐collidine‐CH_2_Cl_2_ instead of AcOH‐CH_3_CN	18

^[a]^ **1a** (0.3 mmol), **2a** (0.2 mmol), FeCl_3_ (15 mol%), Et_4_NCl (1.5 equiv), AcOH (10 equiv) in CH_3_CN (0.1 M, 2 mL); solution sparged with N_2_ prior to irradiation. 52 W LED (λ = 390 nm), undivided cell: C anode/Pt cathode, I = 1.5 mA, *j* = 0.75 mA·cm^−2^, 4.5 F·mol^−1^, 16 hours

^[b]^ Yields determined by ^1^H NMR spectroscopy using CH_2_Br_2_ as external standard. Yield of the isolated product is given in parenthesis.

**Scheme 2:**
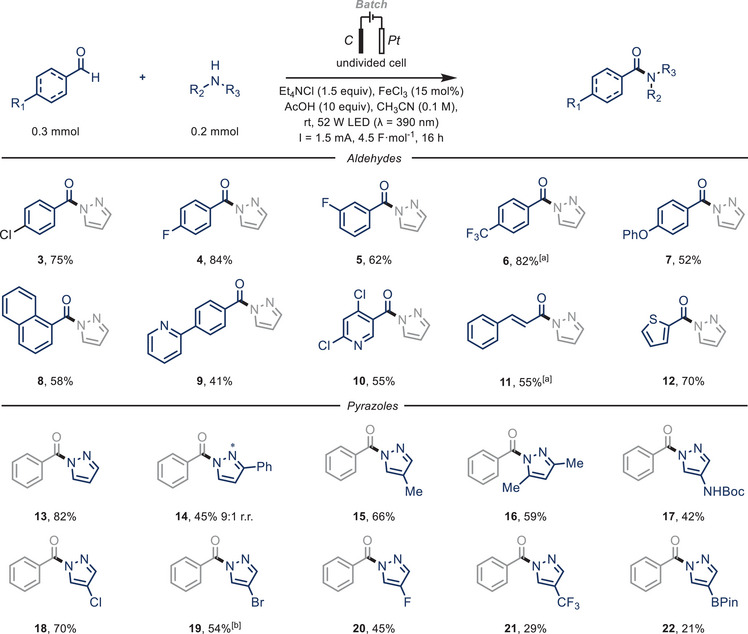
Substrate scope for the electrophotocatalytic C(sp^2^)‒H coupling of aldehydes and azoles. Aldehyde (0.3 mmol), Azole (0.2 mmol), FeCl_3_ (15 mol%), Et_4_NCl (1.5 equiv), AcOH (10 equiv) in CH_3_CN (0.1 M, 2 mL); solution sparged with N_2_ prior to irradiation. 52 W LED (λ = 390 nm), undivided cell: C anode/Pt cathode, I = 1.5 mA, *j* = 0.75 mA·cm^−2^, 4.5 F·mol^−1^, 16 hours. Isolated yields are given. [a] 6.7 F·mol^−1^, 24 hours. [b] Et_4_NBr was used instead of Et_4_NCl. Minor regioisomer is denoted with an asterisk.

Having established the optimized reaction conditions, we shifted our focus to exploring the scope of our electrophotocatalytic transformation, as illustrated in Scheme 2. Initial investigations centered on the coupling of benchmark substrate **2a** with a range of aldehydes. Aromatic aldehydes, particularly those with electron‐withdrawing groups (e.g., chloro **3**, fluoro **4–5**, and trifluoromethyl **6**), yielded the anticipated cross‐coupled products in satisfactory to excellent yields (62–84%). Additionally, we successfully employed aldehydes with electron‐rich (**7**, 52%) and neutral substituents (**8**, 58%). We also achieved effective functionalization of *α*‐*β* unsaturated (**11**, 55%) and heteroaromatic aldehydes (**9,10,12**, 41–70%).

Further exploration involved subjecting a diverse array of pyrazole substrates to our electrophotocatalytic conditions. Efficient functionalization of benzaldehyde (**1k**) with various electron‐neutral (**13**–**14**, 45–82%) and electron‐rich pyrazoles (**15**–**17**, 42–66%) yielded the desired compounds in commendable yields. Notably, halogenated pyrazoles were also successfully converted, although in moderate yields (**18**–**20**, 45–70%). Finally, functionalization of electron‐poor pyrazoles was observed, albeit in lower yields, likely due to their decreased nucleophilicity (**21–22**, 21–29%).

**Scheme 3:**
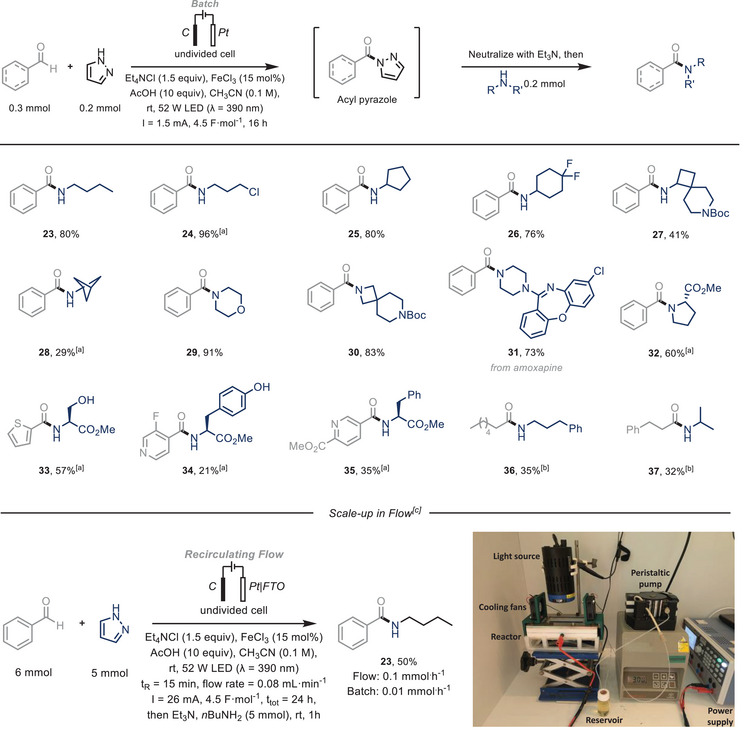
Scheme 3. Substrate scope for the electrophotocatalytic C(sp^2^)‒H coupling of aldehydes and amines. Aldehyde (0.3 mmol), pyrazole (0.2 mmol), FeCl_3_ (15 mol%), Et_4_NCl (1.5 equiv), AcOH (10 equiv) in CH_3_CN (0.1 M, 2 mL); solution sparged with N_2_ prior to irradiation. 52 W LED (λ = 390 nm), undivided cell: C anode/Pt cathode, I = 1.5 mA, *j* = 0.75 mA·cm^−2^, 4.5 F·mol^−1^, 16 hours. Then Et_3_N (10 equiv), Amine (0.2 mmol), 1 hour. Isolated yields are given. [a] for Amine·HCl salts, Et_3_N (11 equiv) was used [b] 1.0 mA, *j* = 0.5 mA·cm^−2^, 2.3 F·mol^−1^, 12 hours. [c] Recirculating flow setup (see Supporting Information): Benzaldehyde (6 mmol), pyrazole (5 mmol), FeCl_3_ (15 mol%), Et_4_NCl (1.5 equiv), AcOH (10 equiv) in CH_3_CN (0.25 M, 16 mL); V_R_ = 1.2 mL, flow rate = 0.08 mL·min^−1^, t_R_ = 15 minutes, I = 26 mA, *j* = 1.08 mA·cm^−2^, 4.5 F·mol^−1^, t_tot_ = 24 hours. Then Et_3_N (10 equiv), *n*‐butylamine (5 mmol), 1 hour.

While proving that a considerable number of aromatic aldehydes can be trapped with pyrazoles under a formal 2‐electron oxidation regime, other nitrogen‐containing five‐membered rings, such as imidazoles or triazoles could not be utilized under the current electrophotocatalytic conditions. While full conversion of the starting material is observed, the desired products could not be isolated due to their sensitivity to hydrolysis (Figure S16). In most of these cases, the *N*‐nucleophile, for example an imidazole, would act as a carbonyl‐activating agent, rendering the aldehyde core highly electrophilic and subsequently, prone to hydrolysis. However, the pyrazole‐coupled products could act themselves as activating agents, and be efficiently converted to the corresponding amides by reacting them with a series of amines (Scheme 3).^[^ ^21^ ^]^ Thus, at the end of the reaction between benzaldehyde (**1k**) and pyrazole (**2a**), without isolation of the acyl pyrazole intermediate, the pH was neutralized with Et_3_N (10 equiv), 0.2 mmol (1 equiv) of the corresponding amine was added, and the reaction was allowed to stir at room temperature for 1 hour. Efficient amide synthesis was achieved with both linear (**23–24**, 80–96%) and cyclic primary amines (**25–28**, 29–80%), while secondary amines yielded excellent results as well (**29–31**, 73–91%). Interestingly, coupling various aldehydes with amino acids also led to efficient amide formation (**32–35**, 21–60%). In general, aliphatic aldehydes react poorly under these conditions, leading to various decomposition products. However, by employing milder conditions (Table S3, Entry 4), we successfully obtained amides from the corresponding aliphatic aldehydes in moderate yields (**36–37**, 28–35%).

Finally, to boost the productivity of the electrophotocatalytic method, we translated the batch conditions to flow. The flow EPC reactor (*f*‐EPC, Figure S2) was fitted with a graphite anode and a platinum‐plated FTO (Fluorine‐doped Tin Oxide) glass electrode, as a more affordable option compared to a full Pt plate. After a quick reoptimization of the conditions (Table S6), we opted for a recirculating flow regime. By applying low current densities, we ensured a low cell potential (typically <1 V) to avoid overoxidation reactions. Following this modified procedure, the coupling of benzaldehyde (**1k**) and butylamine (**3a**), to obtain product **23,** was readily scaled up in flow (5 mmol, 50% isolated yield, Figure 3). This flow system enabled a reliable 10‐fold increase in productivity (0.1 mmol·h^−1^) compared to the batch system (0.01 mmol·h^−1^).

**Scheme 4:**
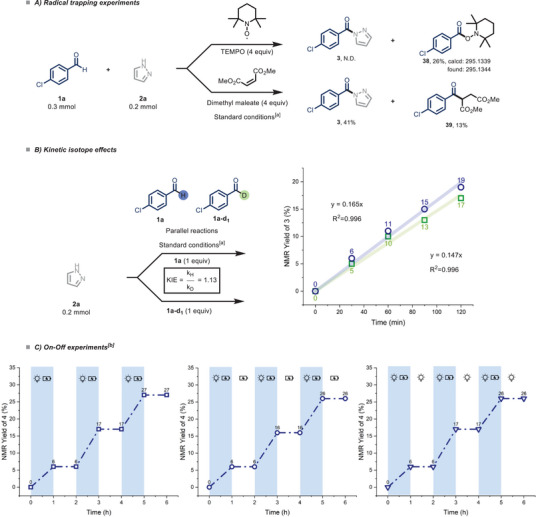
Mechanistic investigations into the electrophotocatalytic aldehyde amidation. A) Radical trapping experiments. B) Kinetic isotope effects (KIEs). C) On‐Off experiments. [a] 1a (0.3 mmol), 2a (0.2 mmol), FeCl_3_ (15 mol%), Et_4_NCl (1.5 equiv), AcOH (10 equiv) in CH_3_CN (0.1 M, 2 mL); solution sparged with N_2_ prior to irradiation. Yields determined by ^1^H NMR spectroscopy using CH_2_Br_2_ as external standard. 52 W LED (λ = 390 nm), undivided cell: C anode/Pt cathode, I = 1.5 mA, j = 0.75 mA^.^cm^−2^ [b] 1b (0.3 mmol), 2a (0.2 mmol), FeCl_3_ (15 mol%), Et_4_NCl (1.5 equiv), AcOH (10 equiv) in CH_3_CN (0.1 M, 2 mL); solution sparged with N_2_ prior to irradiation. 52 W LED (λ = 390 nm), undivided cell: C anode/Pt cathode, I = 1.5 mA, j = 0.75 mA^.^cm^−2^, On‐Off over 6 hours. Yields determined by ^19^F NMR spectroscopy using 1,4‐Difluorobenzene as internal standard.

We performed a series of experiments to further elucidate the reaction mechanism, with our findings presented in Scheme 4. Initially, we conducted the reaction under optimized conditions with the addition of TEMPO ((2,2,6,6‐Tetramethylpiperidin‐1‐yl)oxyl, 4 equivalents), a well‐known radical scavenger. Notably, the reaction was entirely suppressed, and adduct **38** (26% NMR yield, with NMR = nuclear magnetic resonance) was identified via HRMS (high‐resolution mass spectrometry), confirming the involvement of a radical species.^[^ ^22^ ^]^ In parallel, a reaction with an electron‐deficient olefin, dimethyl maleate,^[^ ^23^ ^]^ produced product **39** (13% NMR yield) along with product **3**, indicating the probable formation of an acyl radical via a chlorine‐mediated HAT step (Scheme 4A).

Kinetic Isotope Effect (KIE) evaluations, carried out using a parallel reaction approach, yielded a value close to 1 (KIE = 1.13), suggesting that the HAT is probably not the rate‐determining step (Scheme 4B).^[^ ^24^ ^]^ Lastly, the on‐off experiments explain the requirement for both light and electricity to drive the reaction, implying that the process follows an electrophotocatalytic mechanism (Scheme 4C).

**Scheme 5:**
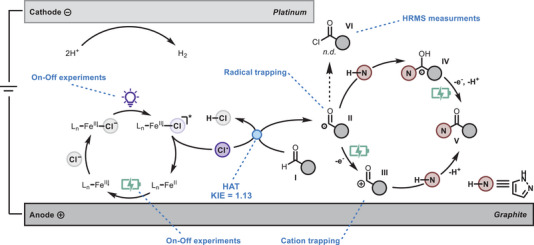
Proposed mechanism of the electrophotocatalytic aldehyde amidation.

A proposed reaction mechanism, depicted in Scheme 5, has been formulated based on these experimental observations. Initially, a chloride anion binds to the iron center, and upon exposure to purple light, the complex is excited to a higher energy state. This excitation leads to the homolytic cleavage of the Fe─Cl bond, producing a chlorine radical and a reduced Fe(II) species.^[^ ^5b^ ^]^ The reduced catalyst is then reoxidized at the anode, restoring the Fe(III) center and completing the catalytic cycle.^[^ ^8^ ^]^ While product formation still occurs in the absence of iron, it is accompanied by significant production of molecular chlorine, which triggers undesirable side reactions.

Interestingly, the presence of iron helps maintain a lower cell potential and prevents overoxidation. The chlorine radical then initiates C(sp^2^)─H bond cleavage from the aldehyde **I**, forming the acyl radical **II**. From this point, two potential pathways emerge. In the first, the acyl radical undergoes electrochemical oxidation, generating the stabilized oxocarbenium ion **III**,^[^ ^25^ ^]^ which is then attacked by a nucleophile, resulting in C─N bond formation. Alternatively, the radical may react directly with the nucleophile, forming the α‐hydroxy radical **IV** via amine‐assisted intermolecular proton transfer.^[^ ^26^ ^]^ This intermediate is subsequently oxidized at the anode to yield the final product **V**, yielding in both cases a radical‐polar crossover (RPC) mechanism. In theory, the acyl radical could also react with trace amounts of solubilized Cl_2_^[^ ^27^ ^]^—produced by anodic oxidation of chloride^[^ ^4d^ ^]^— to form the acyl chloride intermediate **VI**. However, this intermediate was not detected in our experiments, making this pathway unlikely for the formation of the amide product (Figures S8‐S11).

In conclusion, we have successfully developed an oxidant‐free electrophotocatalytic method for the direct coupling of aldehydes and *N*‐nucleophiles. This new synthetic approach to forming C(sp^2^)─N bonds is achieved by simultaneously harnessing photons and electrons in a batch reactor. The products are formed through an electrochemically induced RPC mechanism with HAT photocatalysis, operating under remarkably mild conditions. This method allows for the functionalization of a wide range of organic compounds, facilitating late‐stage functionalization in drug development. The electrophotocatalytic amide formation occurs at room temperature, requires no external oxidants, and its overall productivity can be further enhanced using flow systems. Ongoing studies in our lab are focused on expanding the application of this RPC‐HAT combination to advance synthetic EPC.

## Supporting Information

Additional references cited within the Supporting Information.^[^ ^8^, ^14^, ^15^, ^28^ ^]^

## Conflict of Interest

The authors declare no conflict of interest.

## Supporting information



Supporting Information

## Data Availability

The data that support the findings of this study are available in the supplementary material of this article.
