# Supplementary material for: Oxidant‐Free Amidation of Aldehydes Enabled by Electrophotocatalysis
Source: Chemistry. 2025 Aug 11;31(55):e02237. doi: 10.1002/chem.202502237 (PMC12498078; doi:10.1002/chem.202502237)

Supporting Information

Oxidant-Free Amidation of Aldehydes Enabled by Electrophotocatalysis

Dimitris I. Ioannou,[a,b] Elena Bombonato,[a,c] Jiri Sanramat,[a] Joost N. H. Reek,[b,*] and Timothy Noël[a,*]

[a] Flow Chemistry Group, Van 't Hoff Institute for Molecular Sciences (HIMS), University of Amsterdam, Amsterdam, The Netherlands.

[b] Supramolecular and Homogeneous Catalysis Group, van 't Hoff Institute for Molecular Sciences (HIMS), University of Amsterdam, Amsterdam, The Netherlands.

[c] Department of Chemistry "Giacomo Ciamician", Università di Bologna, Via Selmi, 2, Bologna 40126, Italy.

*Correspondence to: J.N.H.Reek@uva.nl, t.noel@uva.nl.

Table of Contents

Table of Contents 2
1. General Information 3
2. Electrophotochemical reactors 4
2.1 Batch reactor 4
2.2 Flow reactor 5
3. Chart of Starting materials 6
4. Reaction optimization 7
5. General procedure of electrophotocatalytic reactions in batch 10
6. General procedure of electrophotocatalytic reactions in flow 10
7.1 Radical trapping experiments 11
7.2 Kinetic isotope effects 13
7.3 Cation trapping experiments 14
7.4 On-Off experiments 18
7.5 Proposed mechanism 19
8. Limitations of the scope for the direct functionalization of aldehydes 20
9. Characterization Data 21
10. References 32
11. NMR Spectra 33

1. General Information

Materials. All reagents and solvents were used as received without further purification. Reagents and solvents were bought from Sigma Aldrich, TCI and Fluorochem. Technical solvents were bought from VWR International and used as received. Disposable syringes were purchased from Laboratory Glass Specialist. Product isolation was performed automatically, by a Biotage® Isolation Four, with Biotage® SNAP KP-Sil 10 g flash chromatography cartridges, or manually, using silica (P60, SILICYCLE). TLC analysis was performed using Silica on aluminum foils TLC plates (F254, SILICYCLE) with visualization under ultraviolet light (254 nm and 365 nm) or appropriate TLC staining (potassium permanganate). Organic solutions were concentrated under reduced pressure on a Büchi rotary evaporator (in vacuo at 40°C, ~5 mbar). Impervious graphite electrodes for batch and flow experiments were purchased from Gab Neumann (https://www.gabneumann.com). For batch experiments the Electrasyn 2.0 from IKA was used. For flow experiments a power supply (HMP4040, Rohde & Schwarz) and a peristaltic pump (Gilson MINIPULS 3) was used. All capillary tubing and microfluidic fittings were purchased from IDEX Health & Science.

NMR spectroscopy. ^{1}H (300 and 400 MHz), ^{13}C (101 and 128 MHz) and ^{19}F (282 MHz and 376 MHz) spectra were recorded at ambient temperature using Bruker AV 300-I and AV 400. ^{1}H NMR spectra are reported in parts per million (ppm) downfield relative to $CDCl_3$ (7.26 ppm) and all ^{13}C NMR spectra are reported in ppm relative to $CDCl_3$ (77.16 ppm) unless stated otherwise. The multiplicities of signals are designated by the following abbreviations: s (singlet), d (doublet), t (triplet), q (quartet), p (pentet), sext (sextet), m (multiplet), dd (doublet of doublets), dt (doublet of triplets), td (triplet of doublets), ddd (doublet of doublet of doublets). Coupling constants (J) are reported in hertz (Hz). NMR data was processed using the MestReNova 14 software package. Known products were characterized by comparing to the corresponding ^{1}H NMR, ^{13}C NMR and ^{19}F NMR with those available in the literature.

Gas chromatography/mass spectrometry. GC/MS data were collected on an Agilent 5977C GC/MSD system.

Mass spectrometry. High resolution mass spectra (HRMS) were collected on an AccuTOF GC v 4g, JMS-T100GCV Mass spectrometer (JEOL, Japan).

Determination of Regioisomeric Ratio. The regioisomeric ratios were determined by ^{1}H NMR analysis of the purified reaction mixture through integration of diagnostic signals.

2. Electrophotochemical reactors

2.1 Batch reactor

For the batch reactor setup, the ElectraSyn 2.0 from IKA was used combined with Kessil LEDs (390 nm, full intensity) positioned at 5 cm away from the reaction vial, cooled by fans.

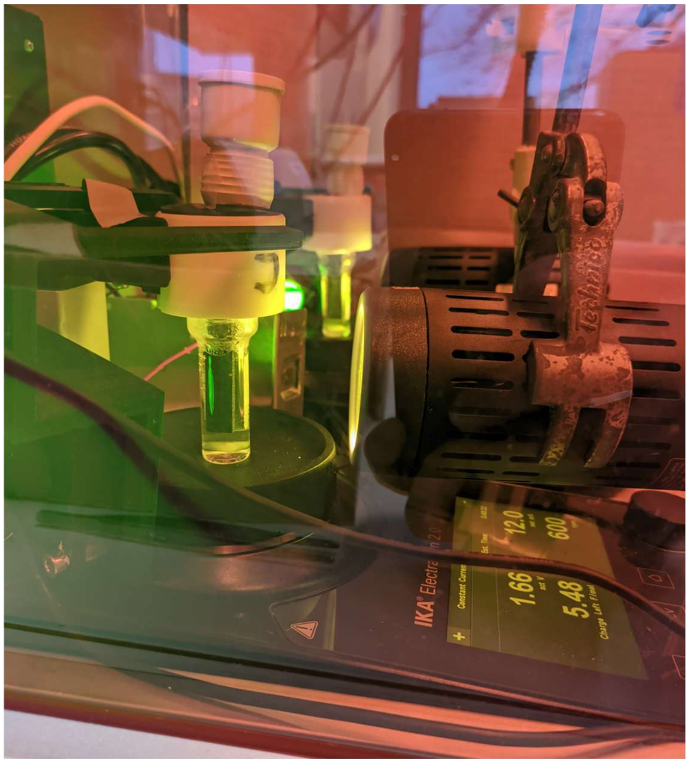

Figure S1: Batch reactor assembly: Orange UV shield, Electrasyn 2.0, Kessil 390 nm LEDs, positioned at 5 cm from the glass, cooling fans. Temperature measured on the glass: 35°C

2.2 Flow reactor

For the flow reactor setup, the *f*-EPC (Flow ElectroPhotoChemical) reactor[1] was combined with Kessil LEDs (390 nm, full intensity) positioned at 5 cm away, cooled by fans.

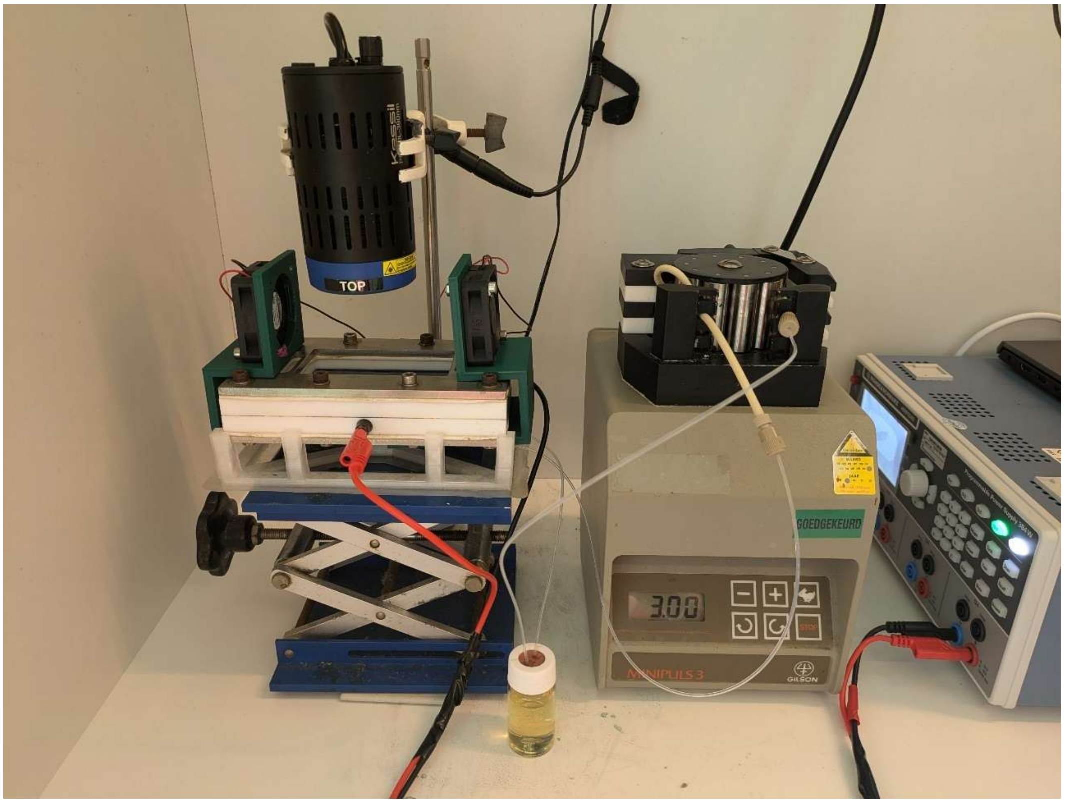

Figure S2: Flow reactor assembly: Kessil 390 nm LEDs, peristaltic pump (Gilson MINIPULS 3), power supply (HMP4040, Rohde & Schwarz). Kessil 390 nm LEDs, positioned at 10 cm from the glass, cooling fans. Temperature measured on the glass: 32˚C

3. Chart of Starting materials

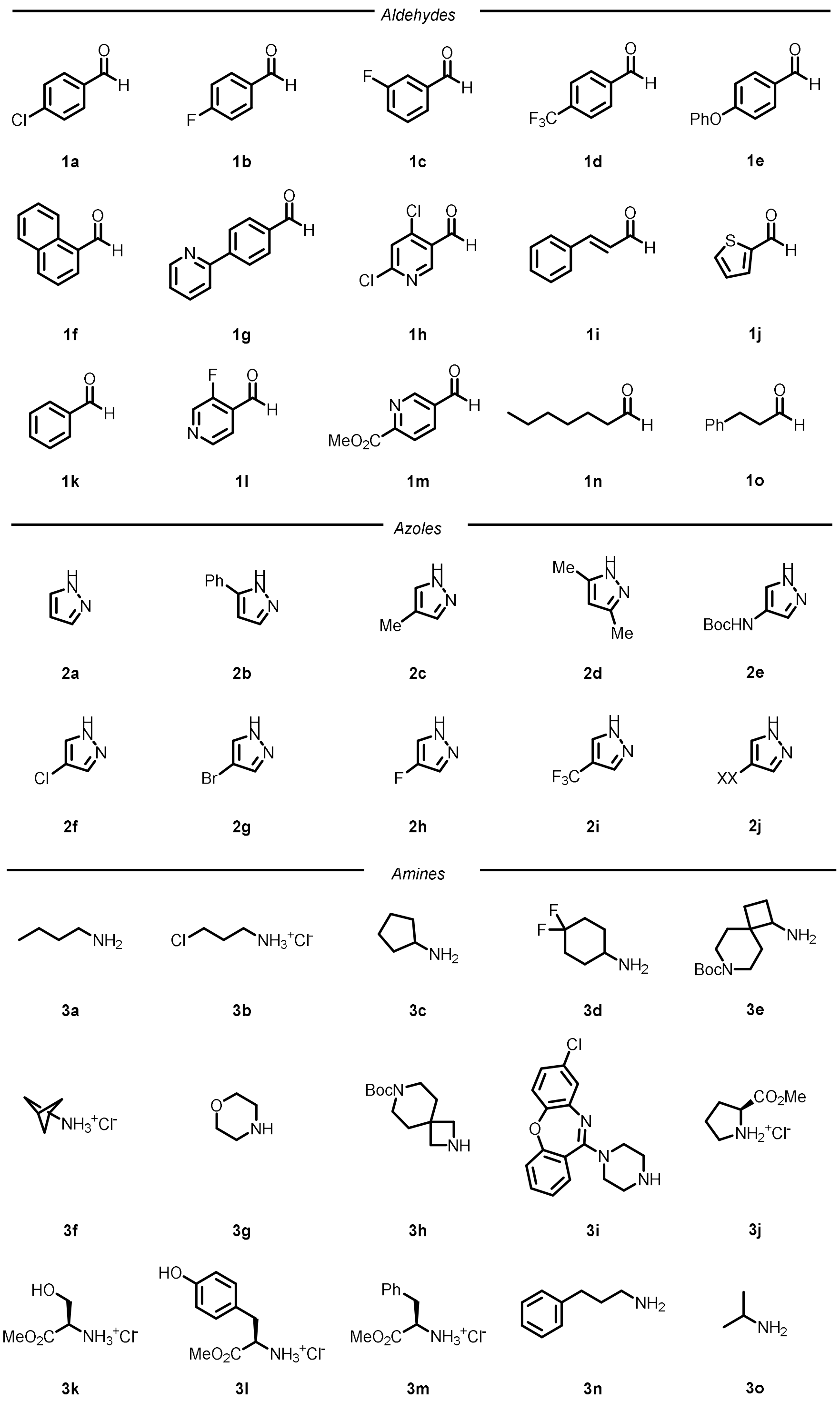

Figure S3: Chart of starting materials

4. Reaction optimization

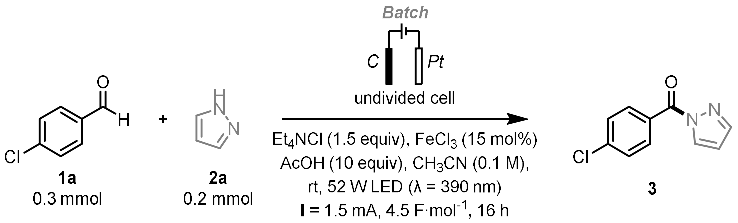

Table S1: Reaction optimization in batch – Control experiments[a]

Entry	Variation from conditions	Yield[b]
1	None	92 (75)
2	Without electricity	8
3	Without light irradiation	--
4	Without electricity or light irradiation	--
5	Without AcOH	<5
6	Without Et_4NCl	69
7	Without $FeCl_3$	34
8	**1a** 0.2 mmol, **2a** 0.6 mmol	90 (74)
9	Acetone as solvent	74
10	2,4,6-collidine, CH_2Cl_2 instead of AcOH, CH_3CN	18

[a] **1a** (0.3 mmol), **2a** (0.2 mmol), $FeCl_3$ (15 mol%), Et_4NCl (1.5 equiv), AcOH (10 equiv) in CH_3CN (0.1 M, 2 mL); solution sparged with N_2 prior to irradiation. 52 W LED (λ = 390 nm), undivided cell: C anode/Pt cathode, I = 1.5 mA, j = 0.75 mA·cm^{-2}, 4.5 F·mol^{-1}, 16 h. [b] Yields determined by ^{1}H NMR spectroscopy using CH_2Br_2 as external standard. Yield of the isolated product is given in parenthesis.

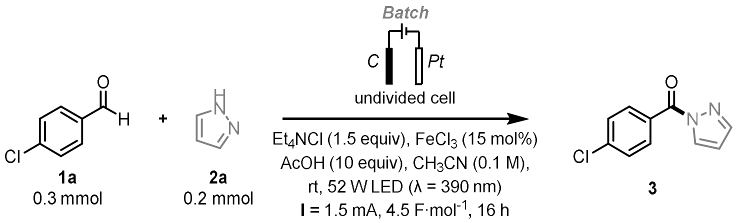

Table S2: Reaction optimization in batch – Photochemical/Electrochemical conditions screening[a]

Entry	Variation from conditions	Yield[b]
1	None	92 (75)
2	5 mA, 2.5 F·mol^{-1}, 2.7 h	48
3	1.5 mA, 2.5 F·mol^{-1}, 9 h	48
4	3 mA, 4.5 F·mol^{-1}, 8 h	66
5	2 mA, 4.5 F·mol^{-1}, 12 h	88
6	$FeCl_3$ (100 mol%) – without electricity	50

[a] **1a** (0.3 mmol), **2a** (0.2 mmol), $FeCl_3$ (15 mol%), Et_4NCl (1.5 equiv), AcOH (10 equiv) in CH_3CN (0.1 M, 2 mL); solution sparged with N_2 prior to irradiation. 52 W LED (λ = 390 nm), undivided cell: C anode/Pt cathode, I = 1.5 mA, j = 0.75 mA·cm^{-2}, 4.5 F·mol^{-1}, 16 h. [b] Yields determined by ^{1}H NMR spectroscopy using CH_2Br_2 as external standard. Yield of the isolated product is given in parenthesis.

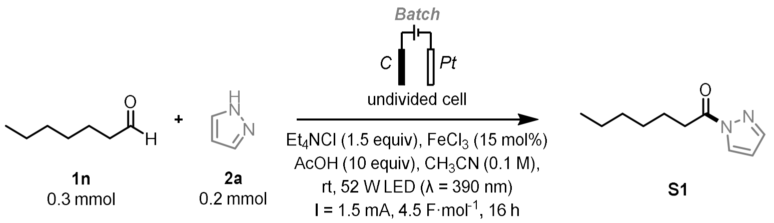

Table S3: Reaction optimization for Aliphatic aldehydes – Photochemical/Electrochemical conditions screening [a]

Entry	Variation from conditions	Yield[b]
1	None	20
2	3 mA, 4.5 F·mol^{-1}, 8 h	25
3	1.5 mA, 2.3 F·mol^{-1}, 8 h	33
4	1.0 mA, 2.3 F·mol^{-1}, 12 h	38
5	0.5 mA, 2.3 F·mol^{-1}, 24 h	24
6	6.0 mA, 2.3 F·mol^{-1}, 2 h	20

[a] **1n** (0.3 mmol), **2a** (0.2 mmol), $FeCl_3$ (15 mol%), Et_4NCl (1.5 equiv), AcOH (10 equiv) in CH_3CN (0.1 M, 2 mL); solution sparged with N_2 prior to irradiation. 52 W LED (λ = 390 nm), undivided cell: C anode/Pt cathode, I = 1.5 mA, *j* = 0.75 mA·cm^{-2}, 4.5 F·mol^{-1}, 16 h. [b] Yields determined by ^1H NMR spectroscopy using CH_2Br_2 as external standard.

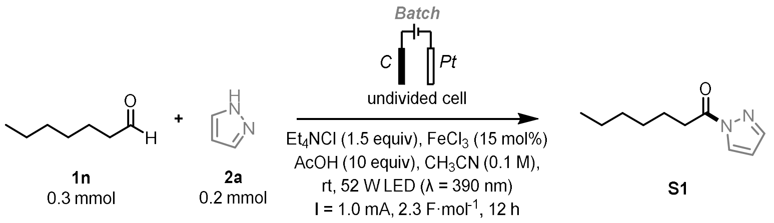

Table S4: Reaction optimization for Aliphatic aldehydes – Additives/Solvents screening[a]

Entry	Variation from conditions	Yield[b]
1	None	38
2	**1n** 0.6 mmol	28
3	**1n** 0.2 mmol, **2a** 0.6 mmol	29
4	25%-75% light intensity	15-25
5	0.5 mA, 2.3 F·mol^{-1}, 24 h	24
6	No AcOH	10
7	No AcOH, KF (2 equiv)	-
8	No AcOH, CH_3CN:HFIP 3:1	-
9	CH_3CN:DCM 1:1	34
10	CH_3CN:Acetone 1:1	35

[a] **1n** (0.3 mmol), **2a** (0.2 mmol), $FeCl_3$ (15 mol%), Et_4NCl (1.5 equiv), AcOH (10 equiv) in CH_3CN (0.1 M, 2 mL); solution sparged with N_2 prior to irradiation. 52 W LED (λ = 390 nm), undivided cell: C anode/Pt cathode, I = 1.0 mA, *j* = 0.5 mA·cm^{-2}, 2.3 F·mol^{-1}, 12 h. [b] Yields determined by ^1H NMR spectroscopy using CH_2Br_2 as external standard.

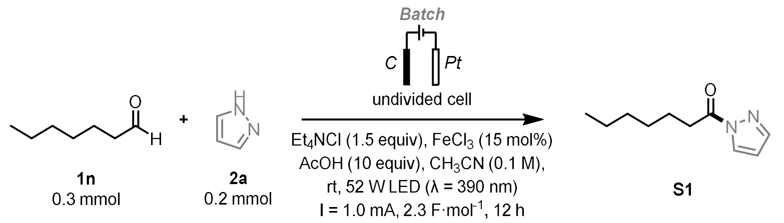

Table S5: Reaction optimization for Aliphatic aldehydes – Miscellaneous screening[a]

Entry	Variation from conditions	Yield[b]
1	None	38
2	$FeCl_3$ (30 mol%)	28
3	$FeCl_3$ (5 mol%)	20
4	$CeCl_3$ (5 mol%), CH_3CN:HFIP 3:1	-
5	$CuCl_2$ (15 mol%)	<5
6	$FeBr_3$ (15 mol%), Et_4NBr (1.5 equiv)	39
7	TBADT (2 mol%)	15
8	**1n**: 1 + 1 equiv (after 4 h)	15

[a] **1n** (0.3 mmol), **2a** (0.2 mmol), $FeCl_3$ (15 mol%), Et_4NCl (1.5 equiv), AcOH (10 equiv) in CH_3CN (0.1 M, 2 mL); solution sparged with N_2 prior to irradiation. 52 W LED (λ = 390 nm), undivided cell: C anode/Pt cathode, I = 1.0 mA, j = 0.5 $mA \cdot cm^{-2}$, 2.3 $F \cdot mol^{-1}$, 12 h. [b] Yields determined by ^1H NMR spectroscopy using CH_2Br_2 as external standard.

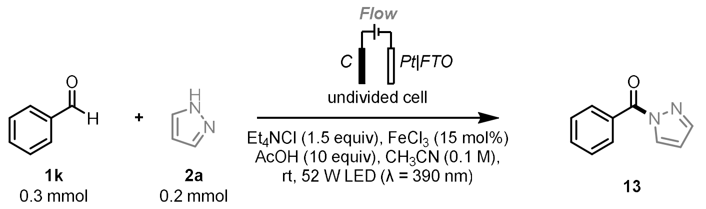

Table S6: Reaction optimization in flow[a]

Entry	Current (mA)	Current density ($mA \cdot cm^{-2}$)	Residence time (min)	Flow rate ($mL \cdot min^{-1}$)	Yield[b]	Throughput ($mmol \cdot h^{-1}$)
1	32 (2.5 $F \cdot mol^{-1}$)	1.33	15	0.08	<5	<0.02
2	44 (4.5 $F \cdot mol^{-1}$)	1.83	20	0.06	10	0.036
3	26 (8 $F \cdot mol^{-1}$)	1.08	60	0.02	9	0.01
4	5 (2.1 $F \cdot mol^{-1}$)	0.21	80	0.015	32	0.029
5 (recirculation)	3.6 (2.1 $F \cdot mol^{-1}$)	0.15	5 (16 h total)	0.24	40 (1 mmol)	0.025
6 (recirculation)	26 (4.5 $F \cdot mol^{-1}$)	1.08	5 (24 h total)	0.24	40 (5 mmol)[c]	0.083
**7 (recirculation)**	**26 (4.5 $F \cdot mol^{-1}$)**	**1.08**	**15 (24 h total)**	**0.08**	**50 (5 mmol)[d]**	**0.1**
8 (batch)	1.5 (4.5 $F \cdot mol^{-1}$)	0.75	16 h	-		0.01

[a] **1k** (0.3 mmol), **2a** (0.2 mmol), $FeCl_3$ (15 mol%), Et_4NCl (1.5 equiv), AcOH (10 equiv) in CH_3CN (0.1 M, 2 mL); solution sparged with N_2 prior to irradiation. 52 W LED (λ = 390 nm), Flow reactor undivided cell: C anode/Pt|FTO cathode. [b] Yields determined by ^1H NMR spectroscopy using CH_2Br_2 as external standard. [c] Isolated yield. [d] Isolated yield after reaction with *n*-butylamine to form product **23**.

5. General procedure of electrophotocatalytic reactions in batch

General Procedure 1 (**GP1**), batch conditions: A solution containing $FeCl_3$ (4.9 mg, 0.03 mmol, 15 mol%), azole (0.2 mmol, 1 equiv.), dry acetonitrile (2 mL), AcOH (115 µL, 2 mmol, 10 equiv.), Et_4NCl (49.7 mg, 0.3 mmol, 1.5 equiv.) and aldehyde (0.3 mmol, 1.5 equiv.) was prepared in a 5-mL vial equipped with an Electrasyn 2.0 cap with septum and stirring bar. On the cap, the graphite anode and the platinum foil cathode were fitted, and the solution was then sparged with nitrogen. The reaction was stirred, irradiated with 52 W 390 nm LEDs and electrolyzed at 1.5 mA for 16 h (0.75 $mA{\cdot}cm^{-2}$, 4.5 $F{\cdot}mol^{-1}$ total charge). After the end of the reaction, the solution was collected, the solvent was removed under reduced pressure and the residue was redissolved in ethyl acetate and washed with water (10 ml) to remove the residual iron catalyst. The organic phases were dried (over $MgSO_4$) and evaporated under reduced pressure, to afford the crude product.

General Procedure 2 (**GP2**), batch conditions: A solution containing $FeCl_3$ (4.9 mg, 0.03 mmol, 15 mol%), pyrazole (13.6 mg, 0.2 mmol, 1 equiv.), dry acetonitrile (2 mL), AcOH (115 µL, 2 mmol, 10 equiv.), Et_4NCl (49.7 mg, 0.3 mmol, 1.5 equiv.) and aldehyde (0.3 mmol, 1.5 equiv.) was prepared in a 5-mL vial equipped with an Electrasyn 2.0 cap with septum and stirring bar. On the cap, the graphite anode and the platinum foil cathode were fitted, and the solution was then sparged with nitrogen. The reaction was stirred, irradiated with 52 W 390 nm LEDs and electrolyzed at 1.5 mA for 16 h (0.75 $mA{\cdot}cm^{-2}$, 4.5 $F{\cdot}mol^{-1}$ total charge). After the end of the reaction, triethylamine (280 µL, 2 mmol, 10 equiv. or 306 µL, 2.2 mmol, 11 equiv. for amine·HCl salts) and the amine (0.2 mmol, 1 equiv.) were added to the reaction mixture. The solution was let to react for 1 hour at room temperature. Then, the solution was collected, the solvent was removed under reduced pressure and the residue was redissolved in ethyl acetate and washed with water (10 ml) to remove the residual iron catalyst. The organic phases were dried (over $MgSO_4$) and evaporated under reduced pressure, to afford the crude product.

6. General procedure of electrophotocatalytic reactions in flow

General Procedure 3 (**GP3**), recirculating-flow conditions: A solution containing $FeCl_3$ (122 mg, 0.75 mmol, 15 mol%), pyrazole (340.4 mg, 5 mmol, 1 equiv.), dry acetonitrile (15 mL), AcOH (2.9 mL, 50 mmol, 10 equiv.), Et_4NCl (1.24 g, 7.5 mmol, 1.5 equiv.) and benzaldehyde (0.77 mL, 7.5 mmol, 1.5 equiv.) was prepared in a 20-mL vial equipped with septum and stirring bar. The solution was then sparged with nitrogen. In the flow reactor, the graphite anode and the FTO/Platinum cathode were fitted. The flow rate was set to 0.08 $mL{\cdot}min^{-1}$ with the peristaltic pump, corresponding to a residence time of 15 min, completing a closed loop with the reactor and the reaction mixture vial. The reaction was then irradiated with 52 W 390 nm LEDs and electrolyzed at 26 mA (1.08 $mA{\cdot}cm^{-2}$, 4.5 $F{\cdot}mol^{-1}$ total charge, 24 h total reaction time). After the end of the reaction, triethylamine (7 mL, 50 mmol, 10 equiv.) and butylamine (0.74 mL, 5 mmol, 1 equiv.) were added to the reaction mixture. The solution was let to react for 1 hour at room temperature. Then, the solution was collected, the solvent was removed under reduced pressure and the residue was redissolved in ethyl acetate and washed with water (100 ml) to remove the residual iron catalyst. The organic phases were dried (over $MgSO_4$) and evaporated under reduced pressure, to afford the crude product.

7. Mechanistic investigations

7.1 Radical trapping experiments

Radical trapping experiments were performed adapting general Procedure 1 (GP1): To an oven-dried vial was added $FeCl_3$ (4.9 mg, 0.03 mmol, 15 mol%), **1a** (42.2 mg, 0.3 mmol, 1.5 equiv), AcOH (115 µL, 2 mmol, 10 equiv.), Et_4NCl (49.7 mg, 0.3 mmol, 1.5 equiv.), **2a** (13.6 mg, 0.2 mmol, 1 equiv), TEMPO (125 mg, 0.8 mmol, 4 equiv.) and dry acetonitrile (2 mL). The mixture was swirled until homogenous, sparged with nitrogen and taken to the reactor. The reaction was then irradiated with 52 W 390 nm LEDs and electrolyzed at 1.5 mA (0.75 $mA \cdot cm^{-2}$, 4.5 $F \cdot mol^{-1}$ total charge). After completion, the solvent was removed under reduced pressure and the residue was redissolved in ethyl acetate and washed with water (10 ml) to remove the residual iron catalyst. The organic phases were dried (over $MgSO_4$) and evaporated under reduced pressure, to afford the crude product. The yield was measured with ^1H NMR, with dibromomethane as external standard. Adduct **38** was observed and identified by HRMS and ^1H NMR indicating the existence of carbon-centered radicals in the solution. HRMS (FD) *m/z* calcd for $C_{16}H_{22}ClNO_2$: 295.1339; found: 295.1344. The spectroscopic data are consistent with those reported previously.[2]

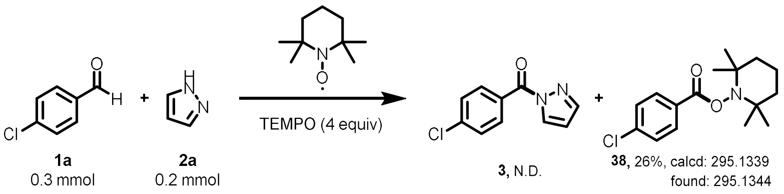

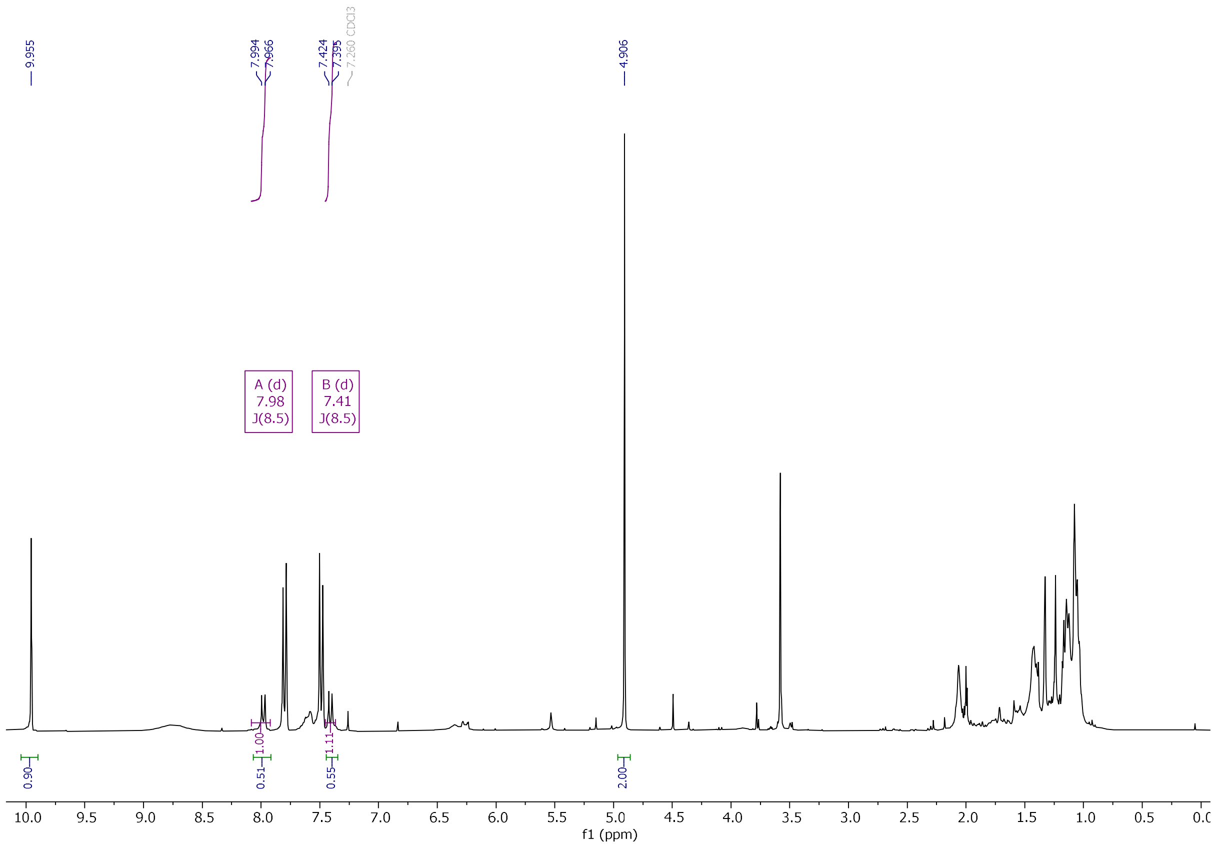

Figure S4: ^1H NMR spectrum of the crude reaction mixture with the TEMPO-adduct

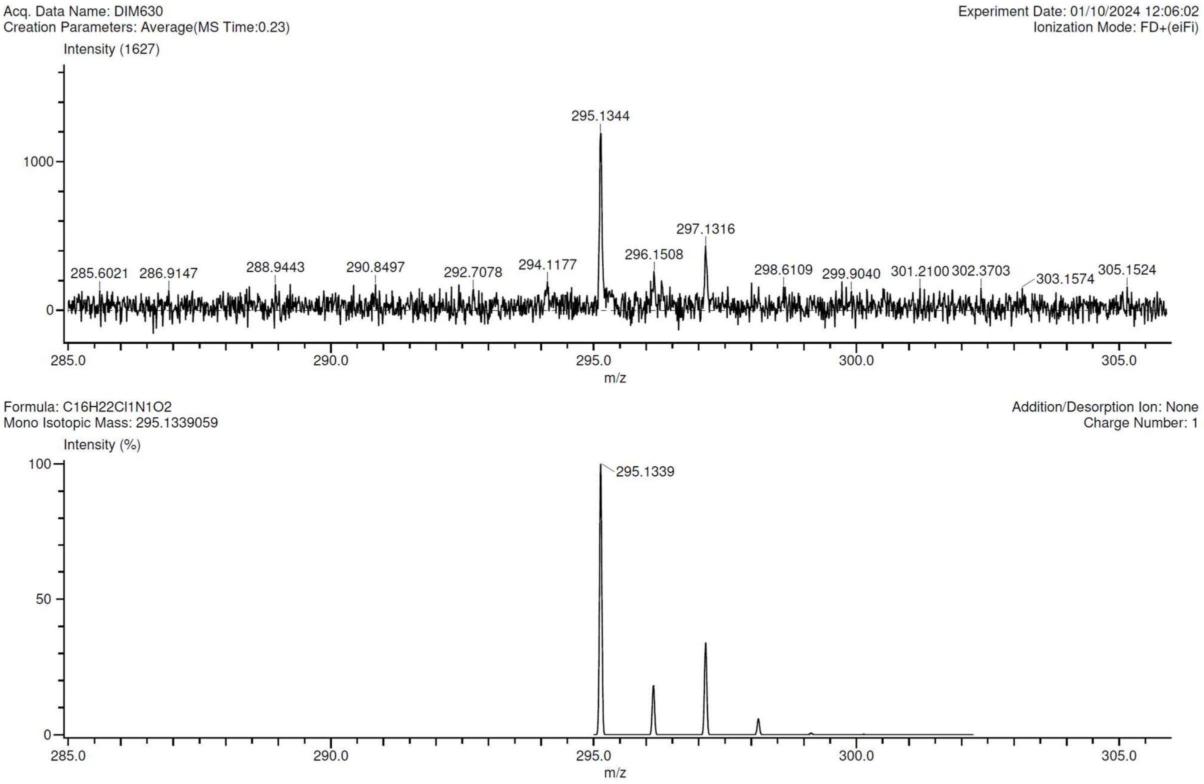

Figure S5: HRMS of the TEMPO-adduct **38**.

To an oven-dried vial was added $FeCl_3$ (4.9 mg, 0.03 mmol, 15 mol%), **1a** (42.2 mg, 0.3 mmol, 1.5 equiv), AcOH (115 µL, 2 mmol, 10 equiv.), Et_4NCl (49.7 mg, 0.3 mmol, 1.5 equiv.), **2a** (13.6 mg, 0.2 mmol, 1 equiv), dimethyl maleate (100 µL, 0.8 mmol, 4 equiv.) and dry acetonitrile (2 mL). The mixture was swirled until homogenous, sparged with nitrogen and taken to the reactor. The reaction was then irradiated with 52 W 390 nm LEDs and electrolyzed at 1.5 mA (0.75 $mA\cdot cm^{-2}$, 4.5 $F\cdot mol^{-1}$ total charge). After completion, the solvent was removed under reduced pressure and the residue was redissolved in ethyl acetate and washed with water (10 ml) to remove the residual iron catalyst. The organic phases were dried (over $MgSO_4$) and evaporated under reduced pressure, to afford the crude product. The yield was measured with ^1H NMR, with trichloroethylene as external standard. Adduct **39** was observed and identified by ^1H NMR indicating the existence of carbon-centered radicals in the solution. The spectroscopic data are consistent with those reported previously.[3]

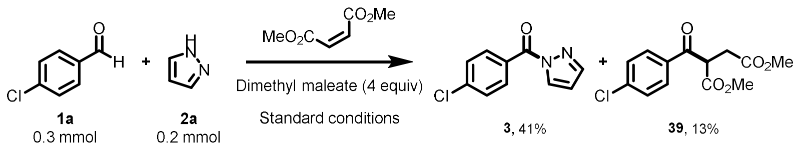

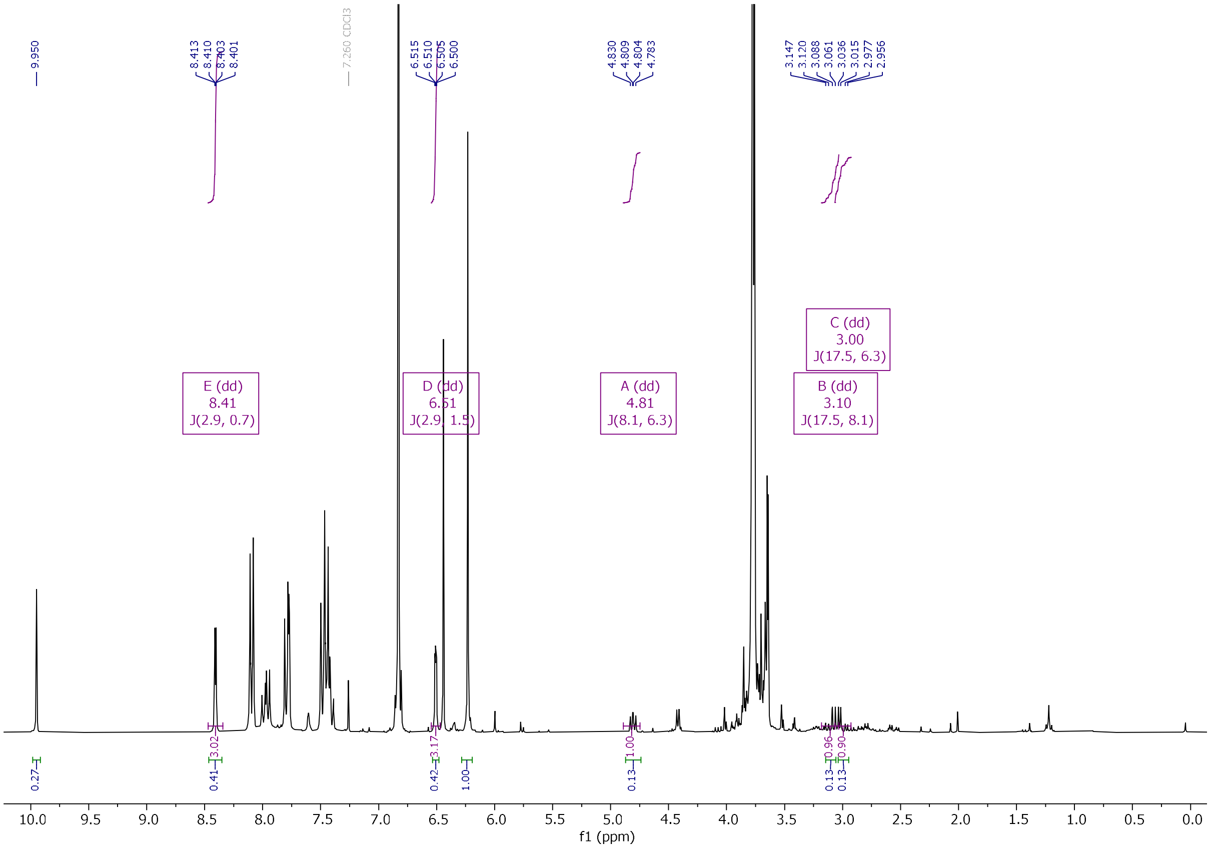

Figure S6: ^1H NMR spectrum of the crude reaction mixture with the dimethyl maleate-adduct

7.2 Kinetic isotope effects

Parallel experiment: The experiment was performed adapting general Procedure 1 (GP1) in batch conditions using **1a** (0.3 mmol, 1.5 equiv.) or 1a-d_1 (0.3 mmol, 1.5 equiv.). A KIE of 1.13 was measured, indicating that hydrogen atom transfer (HAT) is not the rate-determining step (Scheme S5).

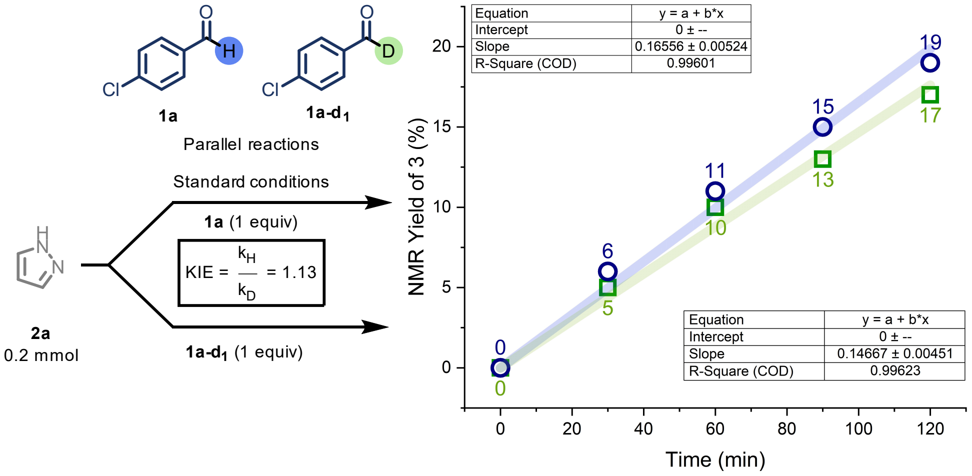

Figure S7: KIE parallel experiments

7.3 Cation trapping experiments

To gain better understanding reaction proceeds through an acyl chloride or a carbocation intermediate, **1a** was allowed to react with **2a** under the model reaction conditions. As expected, product **3** was detected with HRMS, as well as 4-chlorobenzoic acid **S4**. However, no acyl chloride was detected under these conditions. Similarly, in the absence of **2a**, **S4** was detected with HRMS, as well as the corresponding anhydride **S3**. While acetic acid is a weak nucleophile and will not attack the substrate in the presence of pyrazole, the envisioned carbocation would still be quenched by any other nucleophile when **2a** is not present. Notably, 4-chlorobenzyl chloride **S2** was never detected under these conditions (Figures S8-10)

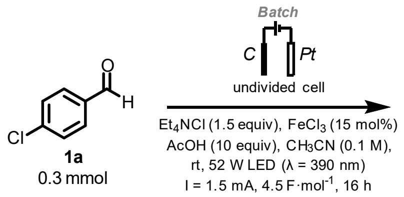

HRMS detection	3	S2	S3	S4
a) With 0.2 mmol 2a	YES	NO	NO	YES
b) Without 2a	NO	NO	YES	YES

Figure S8: Control reactions of **1a** with or without **2a**

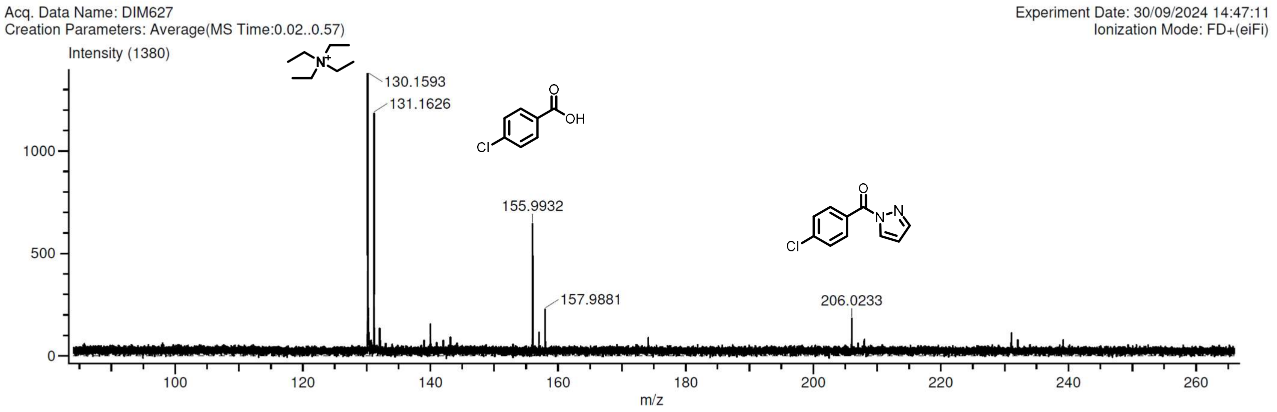

Figure S9: HRMS of the crude mixture of the reaction of **1a** with **2a**

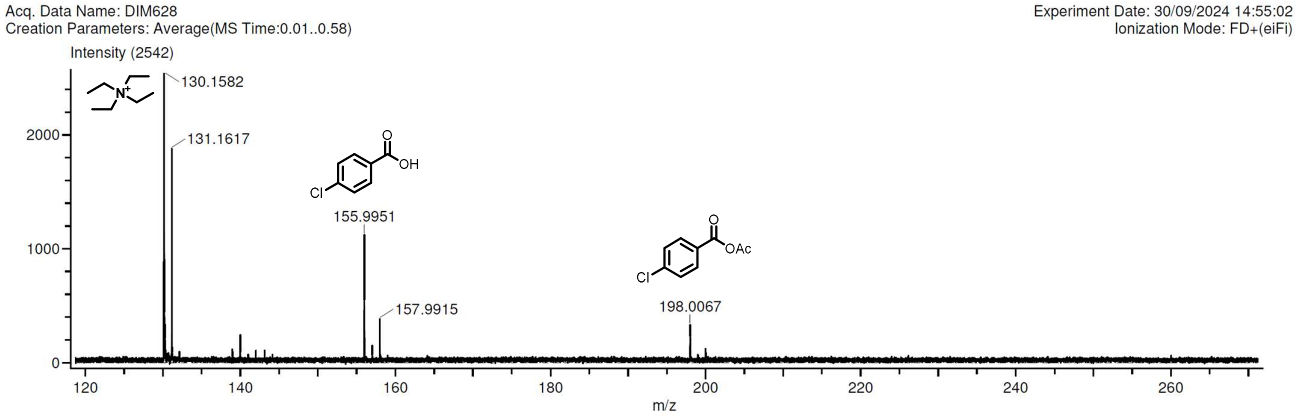

Figure S10: HRMS of the crude mixture of the reaction of **1a** without **2a**

To support the previous results and gain better understanding of the possible intermediates of the reaction, the acyl chloride **S2** was allowed to react with the pyrazole **2a** and at room temperature. Under the neutral or the acidic conditions of the model reaction (MR), the reaction took place successfully, although not to full conversion even after 4 hours. This indicates that, whether a chlorinated intermediate is formed, nucleophilic substitution would lead to the desired product. However, the acyl chloride **S2** could not be substituted by the acetic acid to yield the anhydride **S3**, under model reaction conditions (Figure S11)

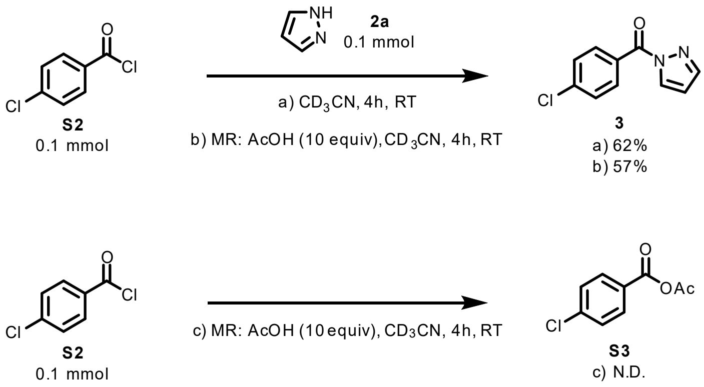

Figure S11: Control reactions of the acyl chloride **S2**

Finally, the benzaldehyde **1a** was allowed to react with methanol (10 equiv). Under conditions of the model reaction (MR), the reaction took place successfully, to yield 18% of the methyl ester **S5**. The spectroscopic data are consistent with those reported previously.[4] With this result, the potential acyl cation intermediate was trapped with 3 different nucleophiles (pyrazole, acetic acid and methanol), supporting the initial hypothesis of its existence.

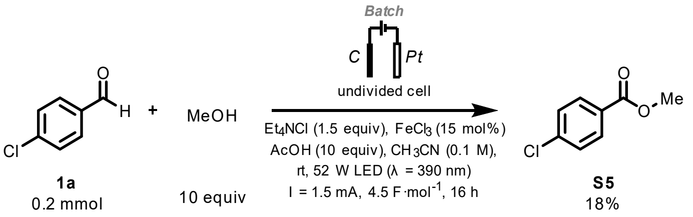

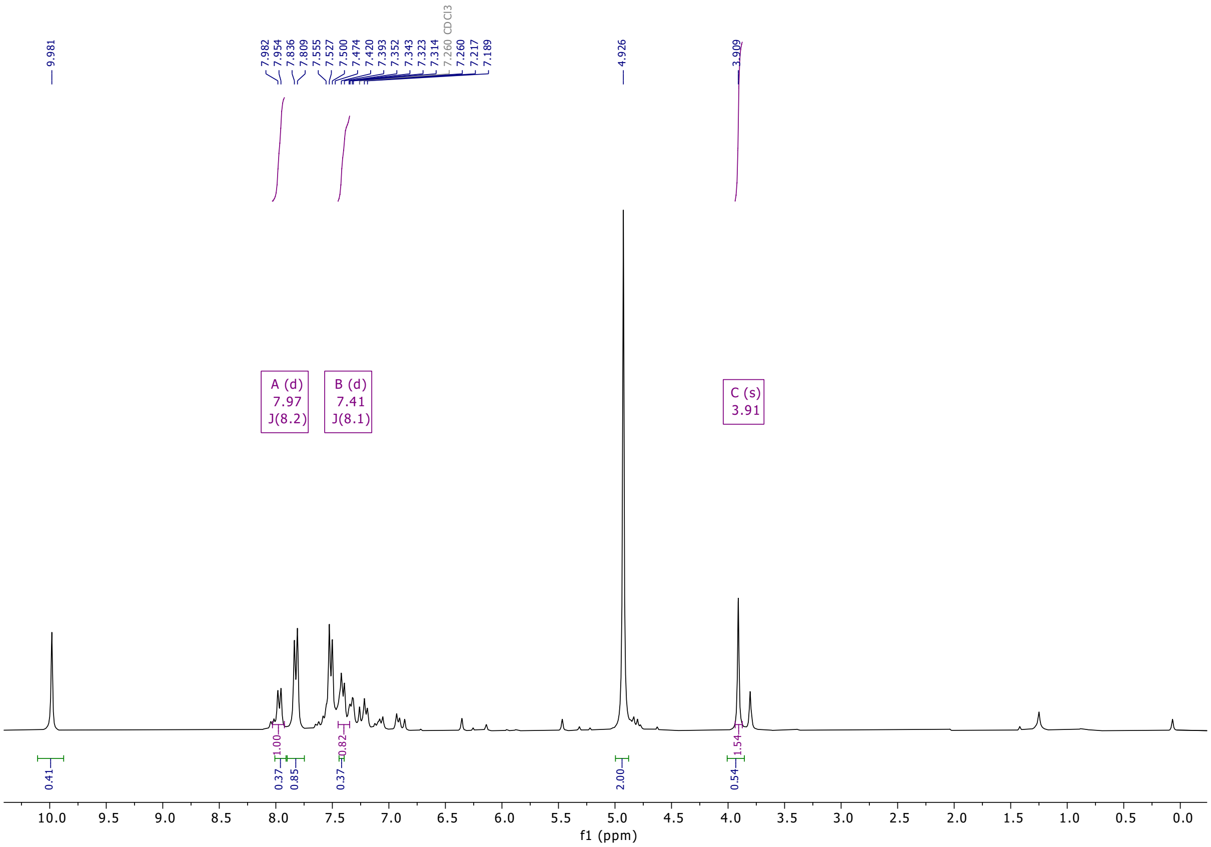

Figure S12: Control reaction of **1a** with methanol

**Batch Path**	D:\data\Dimitris		
**Analysis File Name**	Dimitris.uaf		
**Analyst Name**	Agilent MSD		
**Analysis Time**	15/10/2024 14:30:32		
**Data File Name**	DIM642-a.D	**Data Path Name**	D:\data\Dimitris
**Method Path**	F:\Unknowns methods\default_NIST_search.m	**Method Version**	
**Sample Name**	DIM642-a	**Sample Type**	Sample
**Acq Method File**	default_18min_60to300_fullscan	**Acq Method Path**	F:\Methods\
**Acq Time**	15/10/2024 13:01:59	**Operator**	
**Instrument Name**	AgilentGCMS	**Dilution**	1

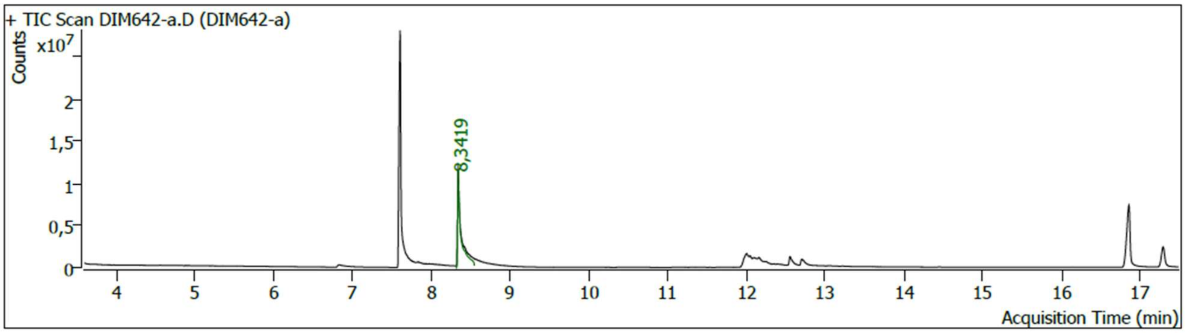

RT	Compound Name	CAS#	Formula	Area	MI	Match Score	Area%-T	Area%-M
8,3419	Benzoic acid, 4-chloro-, methyl ester	1126-46-1	C8H7ClO2	36326685		96,3	100,00	100,00

RT	Compound Name	CAS#	Formula	Area	MI	Match Score	Area%-T	Area%-M
8,3419	Benzoic acid, 4-chloro-, methyl ester	1126-46-1	C8H7ClO2	36326685		96,3	100,00	100,00

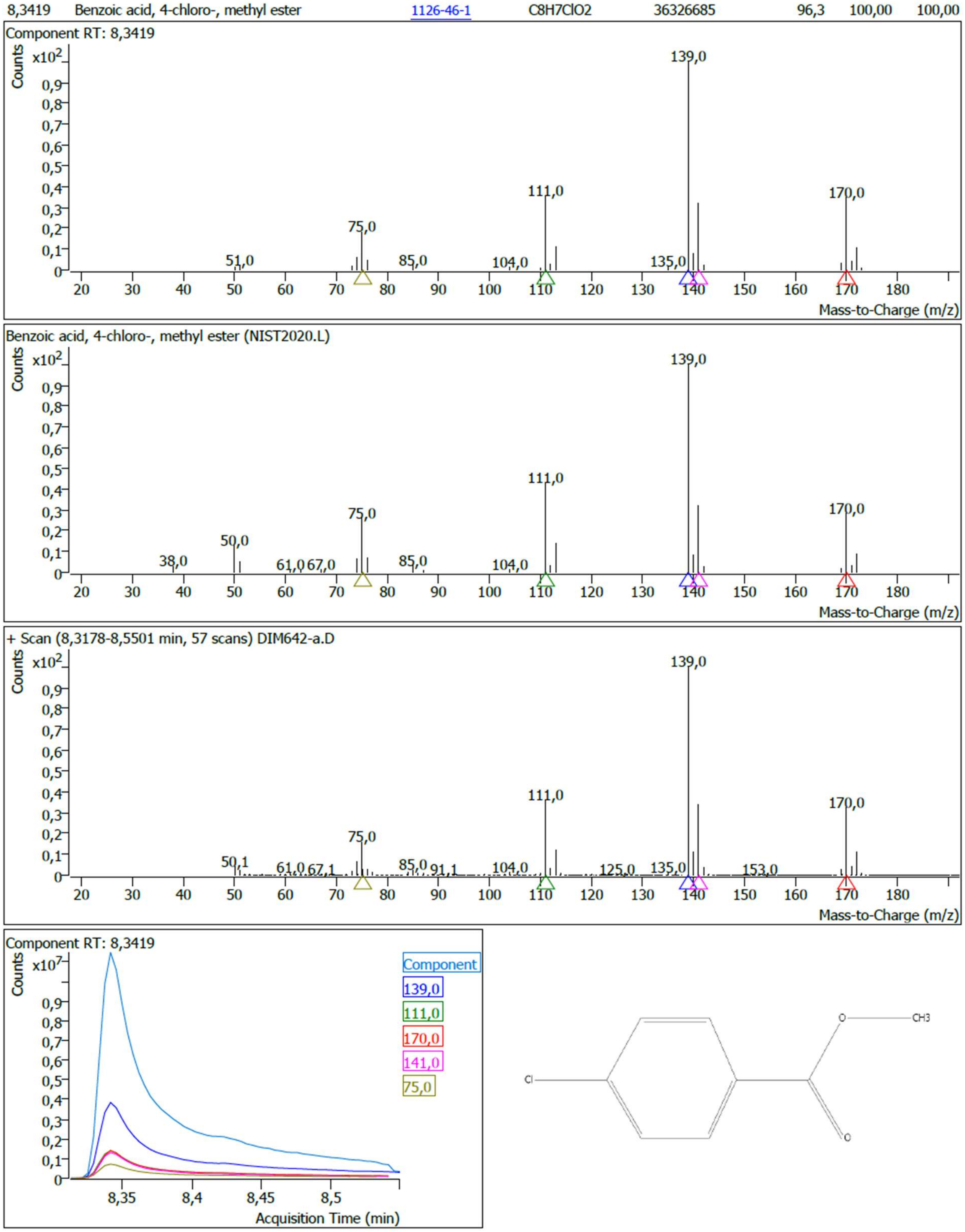

Figure S13: GCMS chromatogram of the crude reaction mixture

7.4 On-Off experiments

The On-Off experiments were performed adapting general Procedure 1 (GP1): To an oven-dried vial was added $FeCl_3$ (4.9 mg, 0.03 mmol, 15 mol%), 4-fluorobenzaldehyde **1b** (32 µL, 0.3 mmol, 1.5 equiv), AcOH (115 µL, 2 mmol, 10 equiv.), Et_4NCl (49.7 mg, 0.3 mmol, 1.5 equiv.), **2a** (13.6 mg, 0.2 mmol, 1 equiv), 1,4-difluorobenzene (20.5 µL, 0.2 mmol, 1 equiv.) and dry acetonitrile (3 mL). The mixture was swirled until homogenous, sparged with nitrogen and taken to the reactor. The reaction was then irradiated with 52 W 390 nm LEDs and electrolyzed at 1.5 mA (0.75 mA·cm^{-2}). 6 cycles of switching electricity and/or light were performed of which samples of 100 µL were taken every 60 min. The yield was measured with ^{19}F NMR, with 1,4-difluorobenzene as internal standard. In all experiments, when light or electricity is absent, the reaction yield remains the same, indicating the combining effect of both in the reaction.

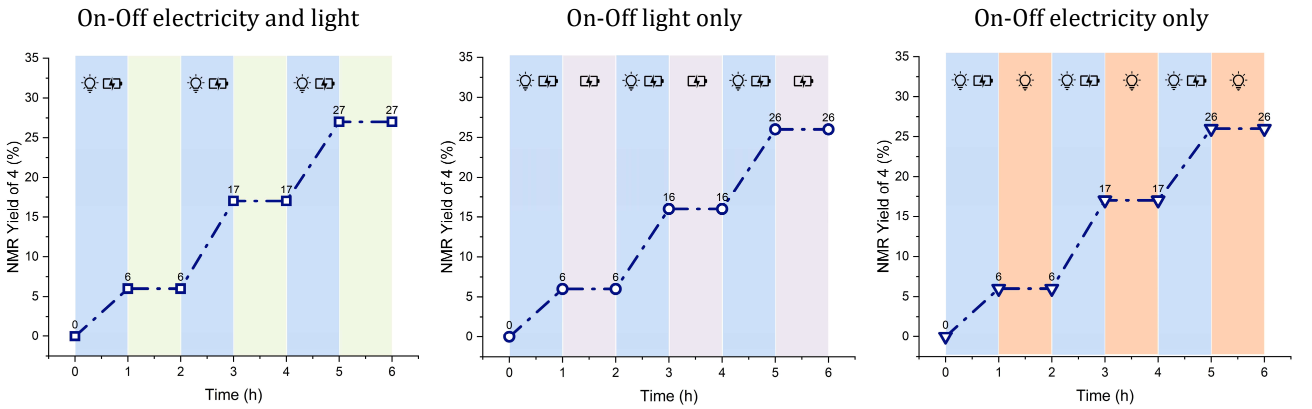

Figure S14: On-Off experiments

7.5 Proposed mechanism

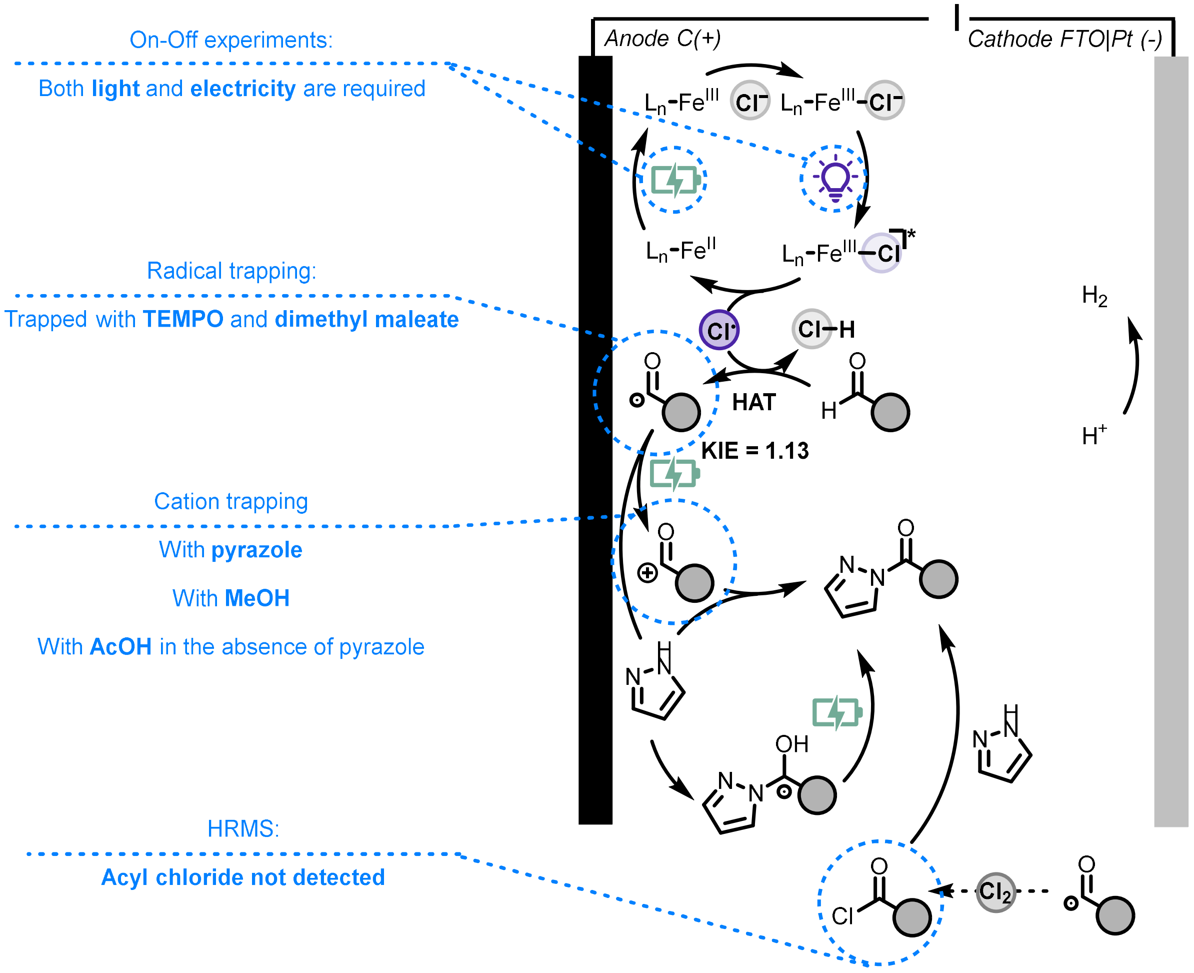

Figure S15: Proposed mechanism

8. Limitations of the scope for the direct functionalization of aldehydes

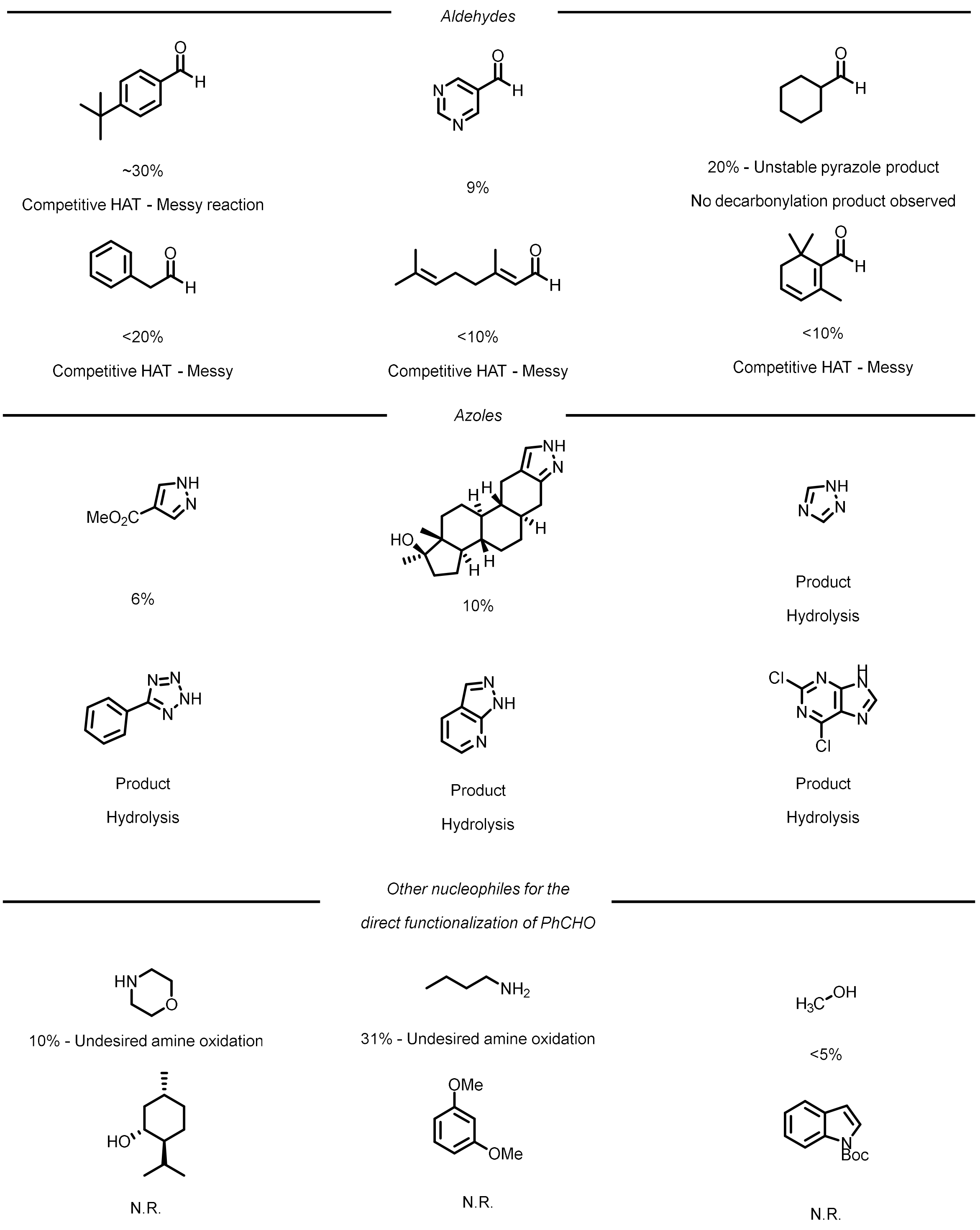

Figure S16: Failed or low-yielding entries of the scope

9. Characterization Data

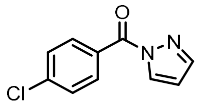

(4-chlorophenyl)(1H-pyrazol-1-yl)methanone (**3**)

Prepared according to GP1. R_f=0.3 (Pentane:Dichloromethane 3:1); purified by flash column chromatography on silica gel (Pentane:Dichloromethane 5:1) to afford the product as a white solid (31 mg, 75%).

^{1}H NMR (300 MHz, $CDCl_3$) δ 8.44 (dd, J = 2.9, 0.7 Hz, 1H), 8.17 – 8.08 (m, 2H), 7.80 (dd, J = 1.5, 0.7 Hz, 1H), 7.53 – 7.44 (m, 2H), 6.54 (dd, J = 2.9, 1.5 Hz, 1H).

^{13}C NMR (101 MHz, $CDCl_3$) δ 165.4, 144.8, 139.8, 133.2, 130.6, 129.9, 128.6, 109.8.

HRMS (FD) m/z calcd for $[M]^+$: 206.0247; found: 206.0250.

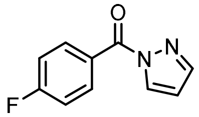

(4-fluorophenyl)(1H-pyrazol-1-yl)methanone (**4**)

Prepared according to GP1. R_f=0.3 (Pentane:Dichloromethane 3:1); purified by flash column chromatography on silica gel (Pentane:Dichloromethane 5:1) to afford the product as a white solid (31.2 mg, 82%).

^{1}H NMR (400 MHz, $CDCl_3$) δ 8.44 (d, J = 2.9 Hz, 1H), 8.23 (dd, J = 8.8, 5.5 Hz, 2H), 7.83 – 7.78 (m, 1H), 7.18 (t, J = 8.6 Hz, 2H), 6.53 (dd, J = 2.9, 1.5 Hz, 1H).

^{13}C NMR (101 MHz, $CDCl_3$) δ 165.8 (d, J = 255.4 Hz), 165.2, 144.7, 134.6 (d, J = 9.2 Hz), 130.7, 127.7 (d, J = 3.1 Hz), 115.5 (d, J = 21.9 Hz), 109.7.

^{19}F NMR (282 MHz, $CDCl_3$) δ -104.7.

The spectroscopic data are consistent with those reported previously.[5]

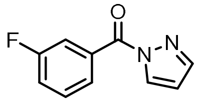

(3-fluorophenyl)(1H-pyrazol-1-yl)methanone (**5**)

Prepared according to GP1. R_f=0.3 (Pentane:Dichloromethane 3:1); purified by flash column chromatography on silica gel (Pentane:Dichloromethane 5:1) to afford the product as a colorless oil (23.6 mg, 62%).

^{1}H NMR (300 MHz, $CDCl_3$) δ 8.44 (dd, J = 2.9, 0.7 Hz, 1H), 7.95 (dt, J = 7.8, 1.3 Hz, 1H), 7.89 (ddd, J = 9.4, 2.6, 1.6 Hz, 1H), 7.85 – 7.78 (m, 1H), 7.49 (td, J = 8.0, 5.5 Hz, 1H), 7.32 (tdd, J = 8.3, 2.7, 1.0 Hz, 1H), 6.54 (dd, J = 2.9, 1.5 Hz, 1H).

^{13}C NMR (101 MHz, $CDCl_3$) δ 165.1 (d, J = 2.9 Hz), 162.2 (d, J = 247.1 Hz), 144.9, 133.5 (d, J = 7.6 Hz), 130.6, 129.9 (d, J = 7.7 Hz), 127.5 (d, J = 3.2 Hz), 120.2 (d, J = 21.2 Hz), 118.8 (d, J = 24.0 Hz), 109.9.

^{19}F NMR (376 MHz, $CDCl_3$) δ -112.1.

HRMS (FD) m/z calcd for $[M]^+$: 190.0542; found: 190.0547.

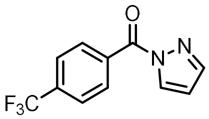

(1H-pyrazol-1-yl)(4-(trifluoromethyl)phenyl)methanone (**6**)

Prepared according to GP1. R_f=0.3 (Pentane:Dichloromethane 3:1); purified by flash column chromatography on silica gel (Pentane:Dichloromethane 5:1) to afford the product as a colorless oil (39.4 mg, 82%).

^{1}H NMR (400 MHz, $CDCl_3$) δ 8.46 (d, J = 2.9 Hz, 1H), 8.23 (d, J = 8.1 Hz, 2H), 7.88 – 7.71 (m, 3H), 6.57 (dd, J = 2.9, 1.5 Hz, 1H).

^{13}C NMR (101 MHz, $CDCl_3$) δ 165.5, 145.2, 135.0, 134.4 (q, J = 32.8 Hz), 131.9, 130.5, 125.2 (q, J = 3.7 Hz), 123.7 (q, J = 272.8 Hz), 110.2.

^{19}F NMR (282 MHz, $CDCl_3$) δ -74.6.

The spectroscopic data are consistent with those reported previously.[5]

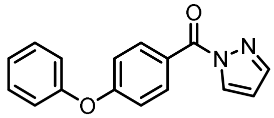

(4-phenoxyphenyl)(1H-pyrazol-1-yl)methanone (**7**)

Prepared according to GP1. R_f=0.3 (Pentane:Dichloromethane 3:1); purified by flash column chromatography on silica gel (Pentane:Dichloromethane 5:1) to afford the product as a colorless oil (27.5 mg, 52%).

^{1}H NMR (300 MHz, $CDCl_3$) δ 8.44 (dd, J = 2.9, 0.7 Hz, 1H), 8.26 – 8.16 (m, 2H), 7.80 (dd, J = 1.4, 0.7 Hz, 1H), 7.46 – 7.35 (m, 2H), 7.25 – 7.17 (m, 1H), 7.14 – 7.08 (m, 2H), 7.08 – 7.01 (m, 2H), 6.51 (dd, J = 2.9, 1.5 Hz, 1H).

^{13}C NMR (75 MHz, $CDCl_3$) δ 165.5, 162.3, 155.4, 144.4, 134.3, 130.6, 130.2, 125.4, 124.9, 120.5, 117.0, 109.3.

HRMS (FD) m/z calcd for $[M]^+$: 264.0899; found: 264.0892.

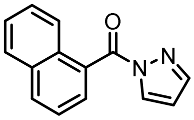

naphthalen-1-yl(1H-pyrazol-1-yl)methanone (**8**)

Prepared according to GP1. R_f=0.3 (Pentane:Dichloromethane 3:1); purified by flash column chromatography on silica gel (Pentane:Dichloromethane 5:1) to afford the product as a yellow solid (25.8 mg, 58%).

^{1}H NMR (400 MHz, $CDCl_3$) δ 8.48 (d, J = 2.8 Hz, 1H), 8.05 (d, J = 8.2 Hz, 1H), 8.00 (dd, J = 6.4, 3.4 Hz, 1H), 7.92 (dd, J = 6.3, 3.3 Hz, 1H), 7.82 (dd, J = 7.2, 1.2 Hz, 1H), 7.76 (d, J = 1.4 Hz, 1H), 7.61 – 7.50 (m, 3H), 6.56 (dd, J = 2.9, 1.4 Hz, 1H).

^{13}C NMR (101 MHz, $CDCl_3$) δ 167.5, 145.0, 133.6, 132.3, 131.0, 130.2, 129.9, 129.0, 128.7, 127.7, 126.6, 125.1, 124.4, 110.1.

The spectroscopic data are consistent with those reported previously.[5]

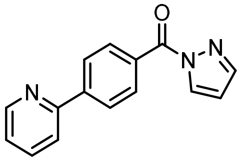

(1H-pyrazol-1-yl)(4-(pyridin-2-yl)phenyl)methanone (**9**)

Prepared according to GP1. R_f=0.3 (Pentane:Ethyl Acetate 9:1); purified by flash column chromatography on silica gel (Pentane:Ethyl Acetate 8:2) to afford the product as a colorless oil (20.5 mg, 41%).

^{1}H NMR (400 MHz, $CDCl_3$) δ 8.76 (d, J = 4.7 Hz, 1H), 8.47 (d, J = 2.8 Hz, 1H), 8.27 (d, J = 8.1 Hz, 2H), 8.16 (d, J = 8.2 Hz, 2H), 7.83 (d, J = 4.2 Hz, 3H), 7.33 (q, J = 4.6 Hz, 1H), 6.55 (dd, J = 2.9, 1.5 Hz, 1H).

^{13}C NMR (101 MHz, $CDCl_3$) δ 166.2, 156.0, 149.8, 144.7, 143.2, 137.5, 132.3, 131.9, 130.6, 126.8, 123.2, 121.4, 109.7.

HRMS (ESI) m/z calcd for $[M+H]^+$: 250.0980; found: 250.0981.

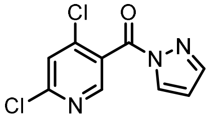

(4,6-dichloropyridin-3-yl)(1H-pyrazol-1-yl)methanone (**10**)

Prepared according to GP1. R_f=0.3 (Pentane:Ethyl Acetate 9:1); purified by flash column chromatography on silica gel (Pentane:Ethyl Acetate 8:2) to afford the product as a white solid (26.6 mg, 55%).

^{1}H NMR (300 MHz, $CDCl_3$) δ 8.55 (s, 1H), 8.41 (dd, J = 2.9, 0.7 Hz, 1H), 7.77 (dd, J = 1.5, 0.7 Hz, 1H), 7.52 (d, J = 0.5 Hz, 1H), 6.59 (dd, J = 2.9, 1.5 Hz, 1H).

^{13}C NMR (101 MHz, $CDCl_3$) δ 163.0, 154.2, 150.2, 145.8, 144.4, 129.3, 128.5, 125.2, 111.3.

HRMS (ESI) m/z calcd for $[M+H]^+$: 241.9888; found: 241.9898.

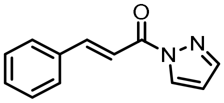

(E)-3-phenyl-1-(1H-pyrazol-1-yl)prop-2-en-1-one (**11**)

Prepared according to GP1. R_f=0.3 (Pentane:Ethyl Acetate 9:1); purified by flash column chromatography on silica gel (Pentane:Ethyl Acetate 8:2) to afford the product as a yellow solid (21.8 mg, 55%).

^{1}H NMR (400 MHz, $CDCl_3$) δ 8.40 (d, J = 2.9 Hz, 1H), 8.04 (d, J = 15.9 Hz, 1H), 7.92 (dd, J = 15.9, 1.3 Hz, 1H), 7.79 (d, J = 1.6 Hz, 1H), 7.74 – 7.67 (m, 2H), 7.47 – 7.40 (m, 3H), 6.50 (dd, J = 2.8, 1.5 Hz, 1H).

^{13}C NMR (101 MHz, $CDCl_3$) δ 163.8, 148.0, 144.0, 134.6, 131.2, 129.1, 129.0, 128.9, 115.9, 110.0.

The spectroscopic data are consistent with those reported previously.[6]

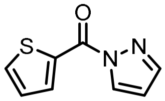

(1H-pyrazol-1-yl)(thiophen-2-yl)methanone (**12**)

Prepared according to GP1. R_f=0.3 (Pentane:Ethyl Acetate 9:1); purified by flash column chromatography on silica gel (Pentane:Ethyl Acetate 8:2) to afford the product as a white solid (25 mg, 70%).

^{1}H NMR (400 MHz, $CDCl_3$) δ 8.47 – 8.37 (m, 2H), 7.85 – 7.77 (m, 2H), 7.19 (dd, *J* = 5.0, 3.9 Hz, 1H), 6.51 (dd, *J* = 2.9, 1.5 Hz, 1H).

^{13}C NMR (101 MHz, $CDCl_3$) δ 159.0, 144.2, 138.7, 137.5, 132.6, 129.9, 127.5, 109.8.

The spectroscopic data are consistent with those reported previously.[7]

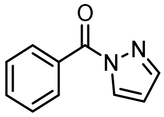

phenyl(1H-pyrazol-1-yl)methanone (**13**)

Prepared according to GP1. R_f=0.3 (Pentane:Dichloromethane 3:1); purified by flash column chromatography on silica gel (Pentane:Dichloromethane 5:1) to afford the product as a colorless oil (28.2 mg, 82%).

^1H NMR (300 MHz, $CDCl_3$) δ 8.44 (d, J = 2.9 Hz, 1H), 8.12 (dt, J = 7.2, 1.4 Hz, 2H), 7.81 (d, J = 1.5 Hz, 1H), 7.68 – 7.56 (m, 1H), 7.51 (dd, J = 8.3, 6.8 Hz, 2H), 6.53 (dd, J = 2.9, 1.5 Hz, 1H).

^{13}C NMR (75 MHz, $CDCl_3$) δ 166.6, 144.6, 133.1, 131.6, 131.6, 130.6, 128.2, 109.6.

The spectroscopic data are consistent with those reported previously.[5]

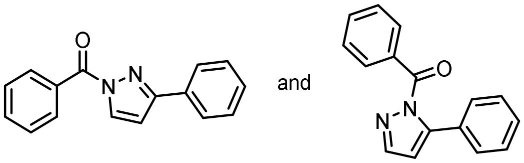

phenyl(3-phenyl-1H-pyrazol-1-yl)methanone (**14**) and phenyl(5-phenyl-1H-pyrazol-1-yl)methanone (**14'**)

Prepared according to GP1. R_f=0.3 (Pentane:Dichloromethane 3:1); purified by flash column chromatography on silica gel (Pentane:Dichloromethane 5:1) to afford the product as a colorless oil as an inseparable mixture of regioisomers (22.3 mg, 45%).

^1H NMR (300 MHz, $CDCl_3$) δ 9.23 (s, 0.1H), 8.48 (d, J = 2.9 Hz, 1H), 8.30 – 8.20 (m, 2H), 7.89 (dd, J = 8.0, 1.6 Hz, 2.2H), 7.69 – 7.60 (m, 1H), 7.57 – 7.49 (m, 2H), 7.48 – 7.35 (m, 3.4H), 6.87 (d, J = 2.9 Hz, 1H), 6.78 (d, J = 2.9 Hz, 0.1H).

^{13}C NMR (101 MHz, $CDCl_3$) δ 166.3, 156.1, 133.1, 132.0, 132.0, 131.9, 131.7, 129.3, 128.9, 128.2, 126.5, 107.3.

Overlapping of peaks avoids the unambiguous ^1H and ^{13}C NMR characterization of the minor isomer.

The spectroscopic data are consistent with those reported previously.[8]

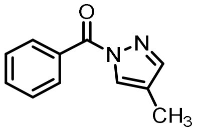

(4-methyl-1H-pyrazol-1-yl)(phenyl)methanone (**15**)

Prepared according to GP1. R_f=0.3 (Pentane:Dichloromethane 3:1); purified by flash column chromatography on silica gel (Pentane:Dichloromethane 5:1) to afford the product as a colorless oil (24.6 mg, 66%).

^1H NMR (300 MHz, $CDCl_3$) δ 8.18 (t, J = 1.0 Hz, 1H), 8.13 – 8.02 (m, 2H), 7.67 – 7.56 (m, 2H), 7.56 – 7.43 (m, 2H), 2.16 (d, J = 1.1 Hz, 3H).

^{13}C NMR (75 MHz, $CDCl_3$) δ 166.4, 146.3, 132.9, 131.9, 131.4, 128.3, 128.2, 120.5, 9.1.

The spectroscopic data are consistent with those reported previously.[8]

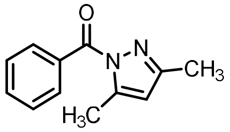

(3,5-dimethyl-1H-pyrazol-1-yl)(phenyl)methanone (**16**)

Prepared according to GP1. R_f=0.3 (Pentane:Dichloromethane 3:1); purified by flash column chromatography on silica gel (Pentane:Dichloromethane 5:1) to afford the product as a colorless oil (23.6 mg, 59%).

^{1}H NMR (400 MHz, $CDCl_3$) δ 8.02 – 7.95 (m, 2H), 7.60 – 7.52 (m, 1H), 7.46 (dd, J = 8.4, 7.0 Hz, 2H), 6.06 (s, 1H), 2.64 (s, 3H), 2.25 (s, 3H).

^{13}C NMR (101 MHz, $CDCl_3$) δ 168.6, 152.3, 145.2, 133.5, 132.6, 131.5, 128.0, 111.2, 14.5, 14.0.

The spectroscopic data are consistent with those reported previously.[9]

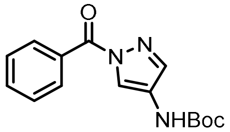

tert-butyl (1-benzoyl-1H-pyrazol-4-yl)carbamate (**17**)

Prepared according to GP1. R_f=0.3 (Pentane:Dichloromethane 3:1); purified by flash column chromatography on silica gel (Pentane:Dichloromethane 5:1) to afford the product as a colorless oil (24.1 mg, 42%).

^{1}H NMR (300 MHz, $CDCl_3$) δ 8.45 (s, 1H), 8.21 – 8.00 (m, 2H), 7.82 (s, 1H), 7.67 – 7.54 (m, 1H), 7.54 – 7.41 (m, 2H), 6.49 (s, 1H), 1.53 (s, 9H).

^{13}C NMR (101 MHz, $CDCl_3$) δ 166.3, 152.6, 137.4, 132.9, 131.6, 131.4, 128.2, 125.2, 118.0, 81.4, 28.4.

HRMS (ESI) m/z calcd for $[M+H]^+$: 288.1343; found: 288.1347.

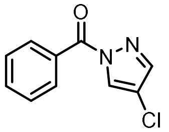

(4-chloro-1H-pyrazol-1-yl)(phenyl)methanone (**18**)

Prepared according to GP1. R_f=0.3 (Pentane:Dichloromethane 3:1); purified by flash column chromatography on silica gel (Pentane:Dichloromethane 5:1) to afford the product as a colorless oil (28.9 mg, 70%).

^{1}H NMR (300 MHz, $CDCl_3$) δ 8.40 (d, J = 0.8 Hz, 1H), 8.17 – 8.02 (m, 2H), 7.72 (d, J = 0.8 Hz, 1H), 7.68 – 7.59 (m, 1H), 7.57 – 7.46 (m, 2H).

^{13}C NMR (75 MHz, $CDCl_3$) δ 165.6, 143.2, 133.5, 131.7, 130.6, 128.3, 128.0, 115.6.

The spectroscopic data are consistent with those reported previously.[8]

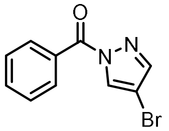

(4-bromo-1H-pyrazol-1-yl)(phenyl)methanone (**19**)

Prepared according to GP1. R_f=0.3 (Pentane:Dichloromethane 3:1); purified by flash column chromatography on silica gel (Pentane:Dichloromethane 5:1) to afford the product as a colorless oil (27.1 mg, 54%).

^{1}H NMR (300 MHz, $CDCl_3$) δ 8.45 (d, *J* = 0.8 Hz, 1H), 8.13 – 8.04 (m, 2H), 7.74 (d, *J* = 0.7 Hz, 1H), 7.67 – 7.60 (m, 1H), 7.55 – 7.47 (m, 2H).

^{13}C NMR (101 MHz, $CDCl_3$) δ 165.4, 145.0, 133.5, 131.7, 130.6, 130.4, 130.4, 128.3, 99.5.

The spectroscopic data are consistent with those reported previously.[8]

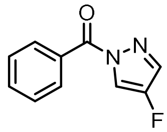

(4-fluoro-1H-pyrazol-1-yl)(phenyl)methanone (**20**)

Prepared according to GP1. R_f=0.3 (Pentane:Dichloromethane 3:1); purified by flash column chromatography on silica gel (Pentane:Dichloromethane 5:1) to afford the product as a white solid (17.1 mg, 45%).

^{1}H NMR (400 MHz, $CDCl_3$) δ 8.26 (d, J = 4.8 Hz, 1H), 8.10 – 8.03 (m, 2H), 7.70 (d, J = 4.4 Hz, 1H), 7.67 – 7.58 (m, 1H), 7.51 (t, J = 7.8 Hz, 2H).

^{13}C NMR (101 MHz, $CDCl_3$) δ 166.4, 151.7 (d, J = 256.4 Hz), 133.9 (d, J = 16.4 Hz), 133.3, 131.7, 130.7, 128.3, 115.3 (d, J = 28.0 Hz).

^{19}F NMR (282 MHz, $CDCl_3$) δ -171.29.

HRMS (FD) m/z calcd for $[M]^+$: 190.0542; found: 190.1549.

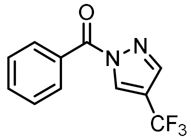

phenyl(4-(trifluoromethyl)-1H-pyrazol-1-yl)methanone (**21**)

Prepared according to GP1. R_f=0.3 (Pentane:Dichloromethane 3:1); purified by flash column chromatography on silica gel (Pentane:Dichloromethane 5:1) to afford the product as a white solid (13.9 mg, 29%).

^{1}H NMR (300 MHz, $CDCl_3$) δ 8.78 – 8.68 (m, 1H), 8.23 – 8.10 (m, 2H), 7.96 (s, 1H), 7.73 – 7.62 (m, 1H), 7.62 – 7.48 (m, 2H).

^{13}C NMR (101 MHz, $CDCl_3$) δ 165.9, 140.6 (d, *J* = 2.5 Hz), 134.0, 131.9, 130.3, 130.3, 128.5, 122.0 (q, *J* = 267.4 Hz), 117.2 (q, *J* = 38.8 Hz).

^{19}F NMR (282 MHz, $CDCl_3$) δ -57.74.

HRMS (FD) m/z calcd for $[M]^+$: 240.0510; found: 240.0512.

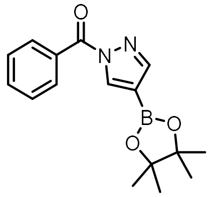

phenyl(4-(4,4,5,5-tetramethyl-1,3,2-dioxaborolan-2-yl)-1H-pyrazol-1-yl)methanone (**22**)

Prepared according to GP1. R_f=0.3 (Pentane:Dichloromethane 3:1); purified by flash column chromatography on silica gel (Pentane:Dichloromethane 5:1) to afford the product as a colorless oil (12.5 mg, 21%).

^{1}H NMR (300 MHz, $CDCl_3$) δ 8.71 (d, *J* = 0.7 Hz, 1H), 8.13 – 8.08 (m, 2H), 8.00 (d, *J* = 0.7 Hz, 1H), 7.65 – 7.58 (m, 1H), 7.54 – 7.47 (m, 2H).

^{11}B NMR (96 MHz, $CDCl_3$) δ 29.54.

^{13}C NMR (75 MHz, $CDCl_3$) δ 166.3, 149.2, 137.9, 133.3, 131.6, 131.6, 128.3, 109.6, 84.1, 24.9.

HRMS (FD) m/z calcd for $[M]^+$: 298.1489; found: 298.1492.

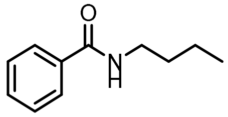

N-butylbenzamide (**23**)

Prepared according to GP2. R_f=0.3 (Pentane:Ethyl Acetate 4:1); purified by flash column chromatography on silica gel (Pentane:Ethyl Acetate 3:1) to afford the product as a colorless oil (28.3 mg, 80%).

^1H NMR (300 MHz, $CDCl_3$) δ 7.84 – 7.69 (m, 2H), 7.52 – 7.36 (m, 3H), 6.28 (s, 1H), 3.44 (td, J = 7.1, 5.7 Hz, 2H), 1.69 – 1.52 (m, 2H), 1.50 – 1.32 (m, 2H), 0.94 (t, J = 7.3 Hz, 3H).

^{13}C NMR (75 MHz, $CDCl_3$) δ 167.7, 135.0, 131.4, 128.6, 127.0, 39.9, 31.8, 20.3, 13.9.

The spectroscopic data are consistent with those reported previously.[10]

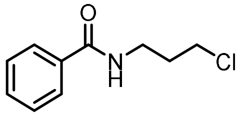

N-(3-chloropropyl)benzamide (**24**)

Prepared according to GP2. R_f=0.3 (Pentane:Ethyl Acetate 4:1); purified by flash column chromatography on silica gel (Pentane:Ethyl Acetate 3:1) to afford the product as a colorless oil (37.7 mg, 96%).

^1H NMR (300 MHz, $CDCl_3$) δ 7.89 – 7.66 (m, 1H), 7.58 – 7.34 (m, 2H), 6.50 (s, 1H), 3.82 – 3.31 (m, 2H), 2.11 (p, J = 6.5 Hz, 1H).

^{13}C NMR (75 MHz, $CDCl_3$) δ 167.9, 134.5, 131.7, 128.7, 127.0, 42.9, 37.8, 32.1.

HRMS (ESI) m/z calcd for $[M+H]^+$: 198.0686; found: 198.0687.

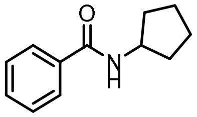

N-cyclopentylbenzamide (**25**)

Prepared according to GP2. R_f=0.3 (Pentane:Ethyl Acetate 4:1); purified by flash column chromatography on silica gel (Pentane:Ethyl Acetate 3:1) to afford the product as a colorless oil (30.3 mg, 80%).

^1H NMR (300 MHz, $CDCl_3$) δ 7.79 – 7.69 (m, 2H), 7.51 – 7.32 (m, 3H), 6.25 (s, 1H), 4.38 (h, J = 7.0 Hz, 1H), 2.14 – 1.97 (m, 2H), 1.80 – 1.53 (m, 4H), 1.57 – 1.39 (m, 2H).

^{13}C NMR (75 MHz, $CDCl_3$) δ 167.3, 135.0, 131.3, 128.6, 126.9, 51.8, 33.3, 23.9.

The spectroscopic data are consistent with those reported previously.[11]

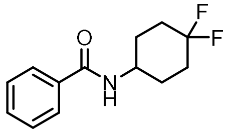

N-(4,4-difluorocyclohexyl)benzamide (**26**)

Prepared according to GP2. R_f=0.3 (Pentane:Ethyl Acetate 4:1); purified by flash column chromatography on silica gel (Pentane:Ethyl Acetate 3:1) to afford the product as a white solid (36.4 mg, 76%).

^{1}H NMR (300 MHz, $CDCl_3$) δ 7.87 – 7.68 (m, 2H), 7.57 – 7.36 (m, 3H), 6.05 (d, J = 8.0 Hz, 1H), 4.11 (td, J = 11.4, 7.5 Hz, 1H), 2.26 – 2.04 (m, 2H), 2.04 – 1.75 (m, 1H), 1.63 (qd, J = 13.0, 4.2 Hz, 1H).

^{13}C NMR (75 MHz, $CDCl_3$) δ 167.2, 134.6, 131.8, 128.8, 127.0, 122.6 (t, J = 241 Hz), 46.9, 32.4 (t, J = 24.8 Hz), 28.9 (d, J = 9.6 Hz).

^{19}F NMR (282 MHz, $CDCl_3$) δ -94.73 (d, J = 237.7 Hz), -101.47 (d, J = 237.7 Hz).

The spectroscopic data are consistent with those reported previously.[12]

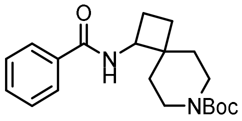

tert-butyl 1-benzamido-7-azaspiro[3.5]nonane-7-carboxylate (**27**)

Prepared according to GP2. R_f=0.3 (Pentane:Ethyl Acetate 4:1); purified by flash column chromatography on silica gel (Pentane:Ethyl Acetate 3:1) to afford the product as a colorless oil (28.2 mg, 41%).

^{1}H NMR (300 MHz, $CDCl_3$) δ 7.81 – 7.67 (m, 2H), 7.57 – 7.31 (m, 3H), 6.29 (d, J = 8.2 Hz, 1H), 4.37 (q, J = 8.6 Hz, 1H), 3.87 – 3.72 (m, 2H), 3.08 – 2.76 (m, 2H), 2.37 (dtd, J = 11.1, 8.5, 2.6 Hz, 1H), 2.02 – 1.70 (m, 3H), 1.69 – 1.51 (m, 4H), 1.42 (s, 9H).

^{13}C NMR (101 MHz, $CDCl_3$) δ 167.3, 154.9, 134.5, 131.6, 128.7, 127.0, 79.5, 51.9, 44.3, 40.3, 37.8, 30.0, 28.6, 25.9, 24.3.

HRMS (ESI) m/z calcd for $[M+H]^+$: 345.2178; found: 345.2182.

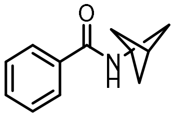

N-(bicyclo[1.1.1]pentan-1-yl)benzamide (**28**)

Prepared according to GP2. R_f=0.3 (Pentane:Ethyl Acetate 4:1); purified by flash column chromatography on silica gel (Pentane:Ethyl Acetate 3:1) to afford the product as a white solid (10.9 mg, 29%).

^{1}H NMR (300 MHz, $CDCl_3$) δ 7.78 – 7.70 (m, 2H), 7.52 – 7.36 (m, 3H), 6.57 (s, 1H), 2.50 (s, 1H), 2.19 (s, 6H).

^{13}C NMR (75 MHz, $CDCl_3$) δ 167.6, 134.6, 131.6, 128.7, 127.0, 53.0, 49.2, 25.1.

The spectroscopic data are consistent with those reported previously.[13]

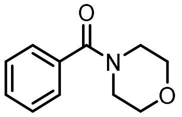

morpholino(phenyl)methanone (**29**)

Prepared according to GP2. R_f=0.3 (Pentane:Ethyl Acetate 4:1); purified by flash column chromatography on silica gel (Pentane:Ethyl Acetate 3:1) to afford the product as a yellow oil (34.4 mg, 90%).[10]

^{1}H NMR (300 MHz, $CDCl_3$) δ 7.47 – 7.33 (m, 5H), 3.94 – 3.35 (m, 8H).

^{13}C NMR (75 MHz, $CDCl_3$) δ 170.5, 135.4, 130.0, 128.6, 127.2, 67.0, 48.1, 42.7.

The spectroscopic data are consistent with those reported previously.

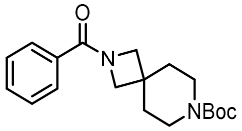

tert-butyl 2-benzoyl-2,7-diazaspiro[3.5]nonane-7-carboxylate (**30**)

Prepared according to GP2. R_f=0.3 (Pentane:Ethyl Acetate 4:1); purified by flash column chromatography on silica gel (Pentane:Ethyl Acetate 3:1) to afford the product as a white solid (54.9 mg, 83%).

^{1}H NMR (300 MHz, $CDCl_3$) δ 7.71 – 7.58 (m, 2H), 7.51 – 7.33 (m, 3H), 4.04 – 3.87 (m, 4H), 3.35 (t, J = 5.7 Hz, 4H), 1.73 (q, J = 5.7 Hz, 4H), 1.44 (s, 9H).

^{13}C NMR (101 MHz, $CDCl_3$) δ 170.6, 154.8, 133.2, 131.2, 128.5, 128.0, 79.9, 63.2, 58.4, 40.8, 35.3, 34.4, 28.5.

The spectroscopic data are consistent with those reported previously.[14]

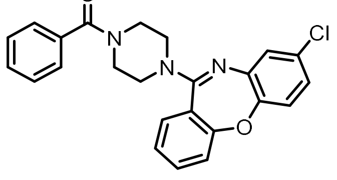

(4-(8-chlorodibenzo[b,f][1,4]oxazepin-11-yl)piperazin-1-yl)(phenyl)methanone (**31**)

Prepared according to GP2. R_f=0.3 (Pentane:Ethyl Acetate 4:1); purified by flash column chromatography on silica gel (Pentane:Ethyl Acetate 3:1) to afford the product as a colorless oil (61 mg, 73%).

^{1}H NMR (400 MHz, $CDCl_3$) δ 7.46 – 7.36 (m, 6H), 7.32 (d, J = 2.6 Hz, 1H), 7.19 (dd, J = 8.5, 1.2 Hz, 1H), 7.17 – 7.05 (m, 3H), 7.01 (td, J = 7.5, 1.9 Hz, 1H), 3.89 (s, 2H), 3.56 (s, 6H).

^{13}C NMR (101 MHz, $CDCl_3$) δ 170.7, 159.5, 158.8, 151.9, 139.8, 135.6, 132.9, 130.6, 130.0, 128.9, 128.7, 127.2, 127.2, 126.0, 125.1, 124.9, 123.0, 120.3, 47.8, 42.1.

HRMS (ESI) m/z calcd for $[M+H]^+$: 418.1322; found: 418.1317.

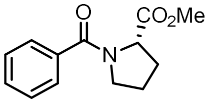

methyl benzoyl-L-prolinate (**32**)

Prepared according to GP2. R_f=0.3 (Pentane:Ethyl Acetate 4:1); purified by flash column chromatography on silica gel (Pentane:Ethyl Acetate 3:1) to afford the product as a white solid (28 mg, 60%).

^{1}H NMR (300 MHz, $CDCl_3$) δ 7.61 – 7.52 (m, 2H), 7.47 – 7.32 (m, 3H), 4.67 (dd, J = 8.4, 5.2 Hz, 1H), 3.77 (s, 3H), 3.65 (dt, J = 10.1, 6.9 Hz, 1H), 3.53 (dq, J = 10.4, 2.4 Hz, 1H), 2.31 (dtd, J = 11.2, 6.8, 2.1 Hz, 1H), 2.03 (dddd, J = 14.4, 11.9, 6.3, 2.2 Hz, 2H), 1.89 (tdd, J = 11.6, 7.0, 3.5 Hz, 1H).

^{13}C NMR (75 MHz, $CDCl_3$) δ 172.9, 169.8, 136.3, 130.3, 128.3, 127.4, 59.2, 52.4, 50.0, 29.5, 25.5.

The spectroscopic data are consistent with those reported previously.[15]

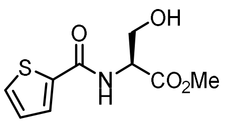

methyl (thiophene-2-carbonyl)-L-serinate (**33**)

Prepared according to GP2. R_f=0.3 (Pentane:Ethyl Acetate 1:2); purified by flash column chromatography on silica gel (Pentane:Ethyl Acetate 1:1) to afford the product as a white solid (26.1 mg, 57%).

^{1}H NMR (400 MHz, $CDCl_3$) δ 7.59 (d, J = 3.5 Hz, 1H), 7.51 (dt, J = 5.0, 1.4 Hz, 1H), 7.08 (dd, J = 5.1, 3.7 Hz, 1H), 7.03 (d, J = 7.2 Hz, 1H), 4.83 (dt, J = 7.1, 3.5 Hz, 1H), 4.05 (qd, J = 11.3, 3.4 Hz, 2H), 3.81 (d, J = 1.7 Hz, 3H).

^{13}C NMR (101 MHz, $CDCl_3$) δ 171.1, 162.3, 138.0, 131.0, 129.0, 127.9, 63.5, 55.2, 53.1.

The spectroscopic data are consistent with those reported previously.[16]

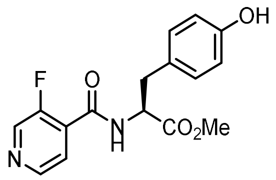

methyl (3-fluoroisonicotinoyl)-L-tyrosinate (**34**)

Prepared according to GP2. R_f=0.3 (Pentane:Ethyl Acetate 1:2); purified by flash column chromatography on silica gel (Pentane:Ethyl Acetate 1:1) to afford the product as a colorless oil (13.4 mg, 21%).

^{1}H NMR (400 MHz, $CDCl_3$) δ 8.61 – 8.52 (m, 2H), 7.90 (t, J = 5.7 Hz, 1H), 7.15 (t, J = 9.1 Hz, 1H), 6.99 (d, J = 8.2 Hz, 2H), 6.75 (d, J = 8.4 Hz, 2H), 5.07 – 4.92 (m, 1H), 3.78 (s, 3H), 3.18 (qd, J = 14.1, 5.7 Hz, 2H).

^{13}C NMR (75 MHz, $CDCl_3$) δ 171.6, 161.0, 155.6, 146.7, 139.4 (d, J = 27.2 Hz), 130.5, 127.6 (d, J = 10.1 Hz), 126.9, 124.7, 115.9, 115.7, 54.3, 52.8, 37.0.

^{19}F NMR (282 MHz, $CDCl_3$) δ -127.95.

HRMS (ESI) m/z calcd for $[M+H]^+$: 319.1094; found: 319.1099.

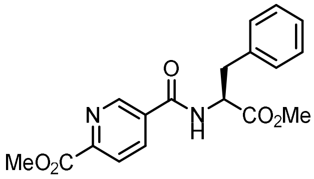

methyl (S)-5-((1-methoxy-1-oxo-3-phenylpropan-2-yl)carbamoyl)picolinate (**35**)

Prepared according to GP1. R_f=0.3 (Pentane:Ethyl Acetate 2:1); purified by flash column chromatography on silica gel (Pentane:Ethyl Acetate 1:1) to afford the product as yellow oil (24 mg, 35%).

^{11}H NMR (300 MHz, $CDCl_3$) δ 8.98 (dd, J = 2.0, 1.1 Hz, 1H), 8.37 – 8.06 (m, 2H), 7.34 – 7.21 (m, 4H), 7.12 (dd, J = 7.6, 1.9 Hz, 2H), 6.64 (d, J = 7.6 Hz, 1H), 5.08 (dt, J = 7.6, 5.7 Hz, 1H), 4.02 (s, 3H), 3.80 (s, 3H), 3.41 – 3.15 (m, 2H).

^{13}C NMR (75 MHz, $CDCl_3$) δ 171.8, 165.0, 164.3, 150.3, 148.2, 136.4, 135.5, 132.4, 129.4, 129.0, 127.6, 125.1, 53.7, 53.3, 52.8, 37.8.

HRMS (ESI) m/z calcd for $[M+H]^+$: 343.1294; found: 343.1299.

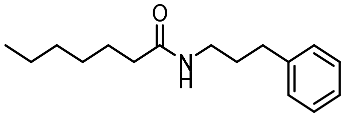

N-(3-phenylpropyl)heptanamide (**36**)

Prepared according to GP2. R_f=0.3 (Pentane:Ethyl Acetate 3:1); purified by flash column chromatography on silica gel (Pentane:Ethyl Acetate 2:1) to afford the product as a colorless oil (17.3 mg, 35%).

^{1}H NMR (300 MHz, $CDCl_3$) δ 7.35 – 7.24 (m, 2H), 7.22 – 7.15 (m, 3H), 5.36 (s, 1H), 3.29 (td, J = 7.2, 5.9 Hz, 2H), 2.65 (t, J = 7.6 Hz, 2H), 2.25 – 2.02 (m, 2H), 1.94 – 1.76 (m, 2H), 1.60 (d, J = 7.2 Hz, 2H), 1.28 (ddt, J = 7.5, 5.5, 2.4 Hz, 6H), 1.03 – 0.78 (m, 3H).

^{13}C NMR (101 MHz, $CDCl_3$) δ 173.3, 141.6, 128.6, 128.5, 126.1, 39.3, 37.0, 33.5, 31.7, 31.4, 29.1, 25.9, 22.6, 14.1.

HRMS (ESI) m/z calcd for $[M+H]^+$: 248.2009; found: 248.2016.

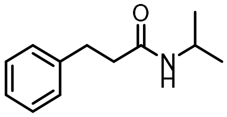

N-isopropyl-3-phenylpropanamide (**37**)

Prepared according to GP2. R_f=0.3 (Pentane:Ethyl Acetate 3:1); purified by flash column chromatography on silica gel (Pentane:Ethyl Acetate 2:1) to afford the product as a white solid (12.2 mg, 32%).

^{1}H NMR (300 MHz, $CDCl_3$) δ 7.33 – 7.24 (m, 2H), 7.22 – 7.15 (m, 3H), 5.25 (s, 1H), 4.03 (dp, *J* = 8.0, 6.6 Hz, 1H), 2.94 (dd, *J* = 8.4, 7.0 Hz, 2H), 2.42 (dd, *J* = 8.4, 7.0 Hz, 2H), 1.06 (d, *J* = 6.6 Hz, 6H).

^{13}C NMR (101 MHz, $CDCl_3$) δ 171.3, 141.0, 128.6, 128.5, 126.3, 41.4, 38.8, 32.0, 22.8, 22.8.

The spectroscopic data are consistent with those reported previously.[17]

10. References

[1] D. I. Ioannou, L. Capaldo, J. Sanramat, J. N. H. Reek, T. Noël, *Angew. Chem. Int. Ed.* **2023**, *62*, e202315881.

[2] Y. Man, B. Xu, *Org. Lett.* **2024**, *26*, 2456-2461.

[3] M. A. Maskeri, M. L. Schrader, K. A. Scheidt, *Chem. Eur. J.* **2020**, *26*, 5794-5798.

[4] H. Zhai, Z. Wei, X. Jing, C. Duan, *Inorg. Chem.* **2024**, *63*, 14375-14382.

[5] F. Politano, A. León Sandoval, M. L. Witko, K. E. Doherty, C. M. Schroeder, N. E. Leadbeater, *Eur. J. Org. Chem.* **2022**, *2022*, e202101239.

[6] J. M. Ovian, C. B. Kelly, V. A. Pistritto, N. E. Leadbeater, *Org. Lett.* **2017**, *19*, 1286-1289.

[7] X. Wang, S. Gao, J. Yang, Y. Gao, L. Wang, X. Tang, *Nat. Prod. Res.* **2016**, *30*, 682-688.

[8] J. Luo, Q. Zhou, Z. Xu, K. N. Houk, K. Zheng, *J. Am. Chem. Soc.* **2024**, *146*, 21389-21400.

[9] Y.-F. Liu, K. Li, H.-Y. Lian, X.-J. Chen, X.-L. Zhang, G.-P. Yang, *Inorg. Chem.* **2022**, *61*, 20358-20364.

[10] J. M. L. Elwood, M. C. Henry, J. D. Lopez-Fernandez, J. M. Mowat, M. Boyle, B. Buist, K. Livingstone, C. Jamieson, *Org. Lett.* **2022**, *24*, 9491-9496.

[11] H. Gao, L. Guo, Y. Zhu, C. Yang, W. Xia, *Chem. Commun.* **2023**, *59*, 2771-2774.

[12] S. Holovach, K. P. Melnykov, A. Skreminskiy, M. Herasymchuk, O. Tavlui, D. Aloshyn, P. Borysko, A. B. Rozhenko, S. V. Ryabukhin, D. M. Volochnyuk, O. O. Grygorenko, *Chem. Eur. J.* **2022**, *28*, e202200331.

[13] D. S. Toops, M. R. Barbachyn, *J. Org. Chem.* **1993**, *58*, 6505-6508.

[14] A. Wang, Y. Li, K. Lv, R. Gao, A. Wang, H. Yan, X. Qin, S. Xu, C. Ma, J. Jiang, Z. Wei, K. Zhang, M. Liu, *Eur. J. Med. Chem.* **2021**, *222*, 113591.

[15] N. Decha, J. Thonglam, J. Meesane, S. Pornsuwan, C. Tansakul, *Org. Biomol. Chem.* **2024**, *22*, 1254-1268.

[16] G. E. Evenson, W. C. Powell, A. B. Hinds, M. A. Walczak, *J. Org. Chem.* **2023**, *88*, 6192-6202.

[17] X. Yang, X. Tian, N. Sun, B. Hu, Z. Shen, X. Hu, L. Jin, *Organometallics* **2023**, *42*, 38-47.

11. NMR Spectra

^{1}H NMR (300 MHz, Chloroform-*d*)

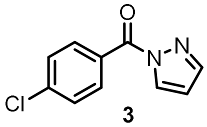

3

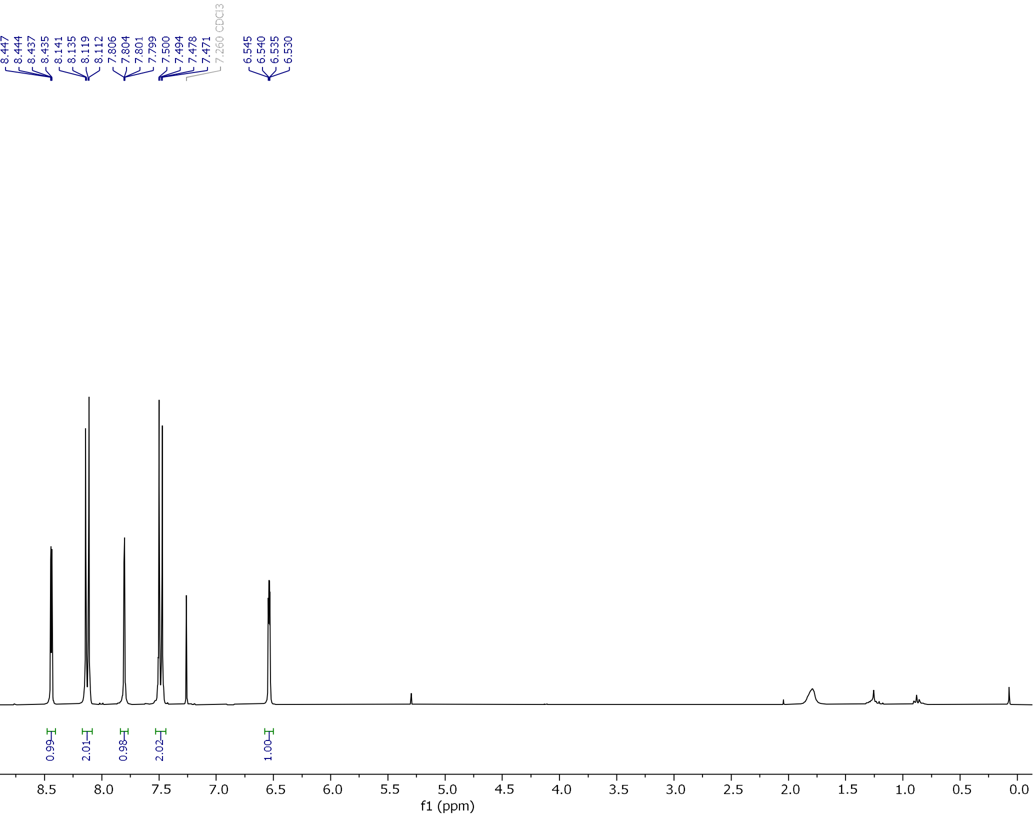

^{13}C NMR (101 MHz, Chloroform-*d*)

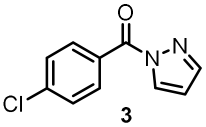

3

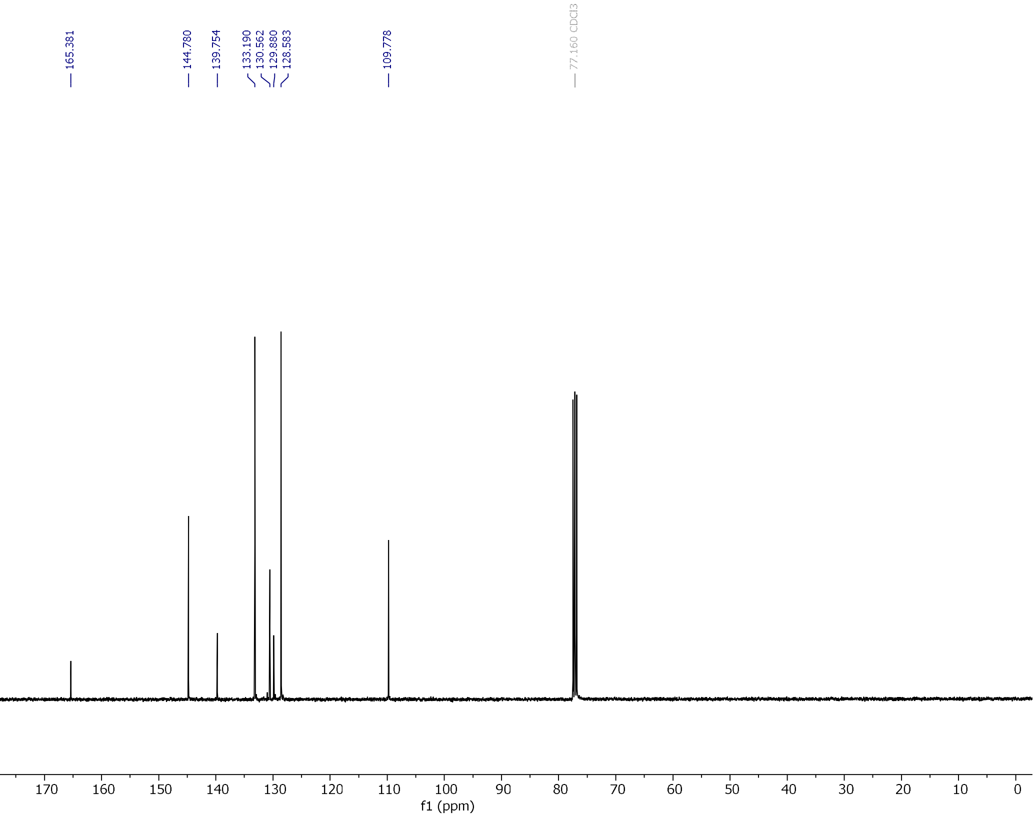

^{1}H NMR (400 MHz, Chloroform-*d*)

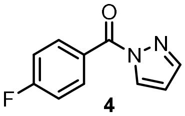

4

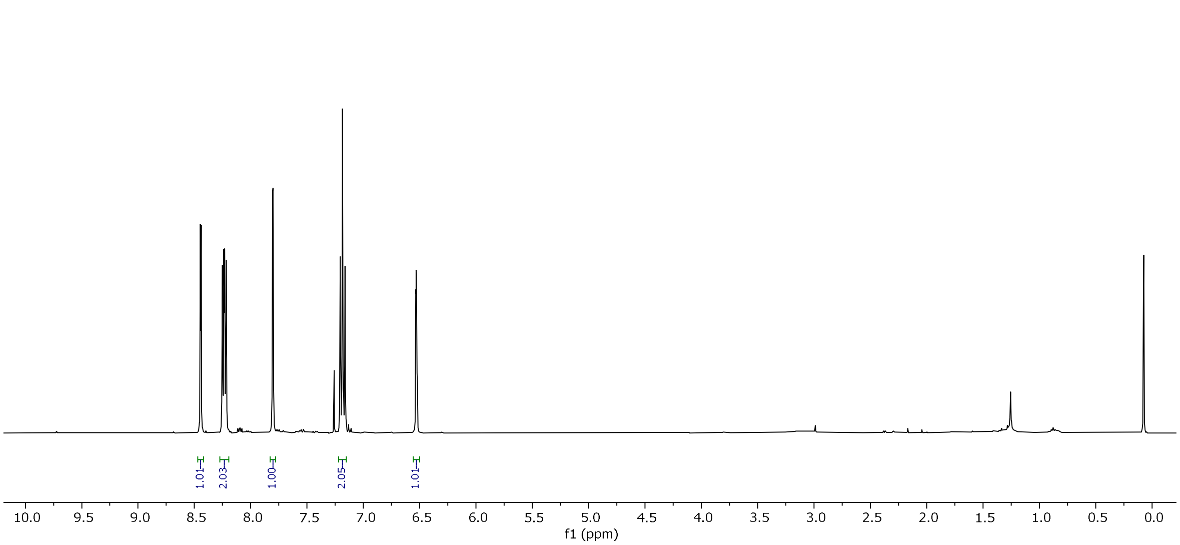

^{13}C NMR (101 MHz, Chloroform-*d*)

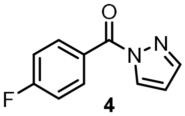

4

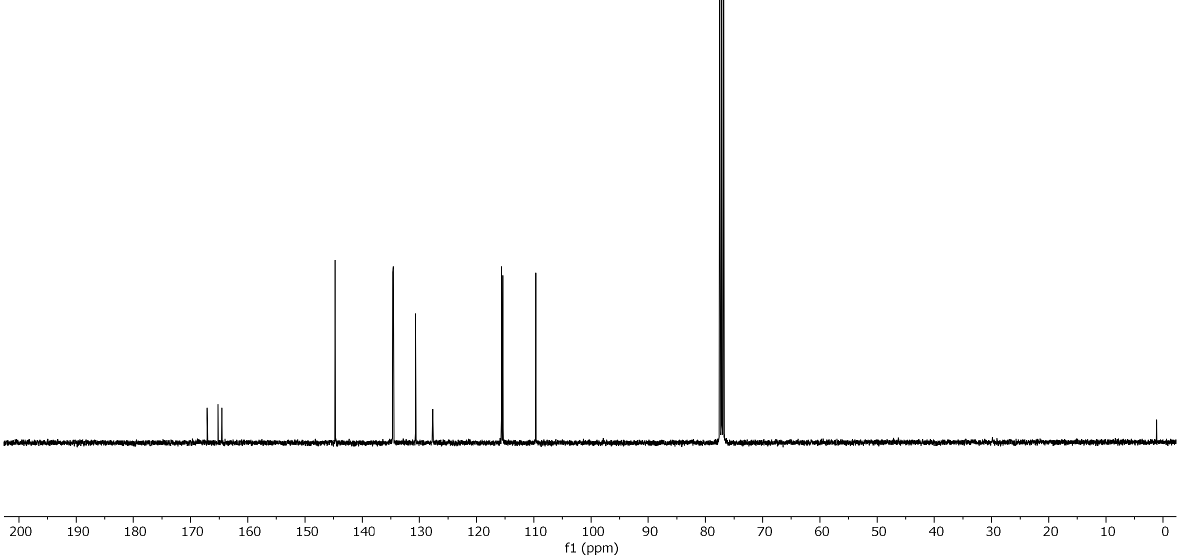

^{19}F NMR (282 MHz, Chloroform-*d*)

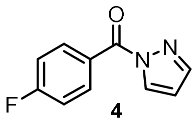

4

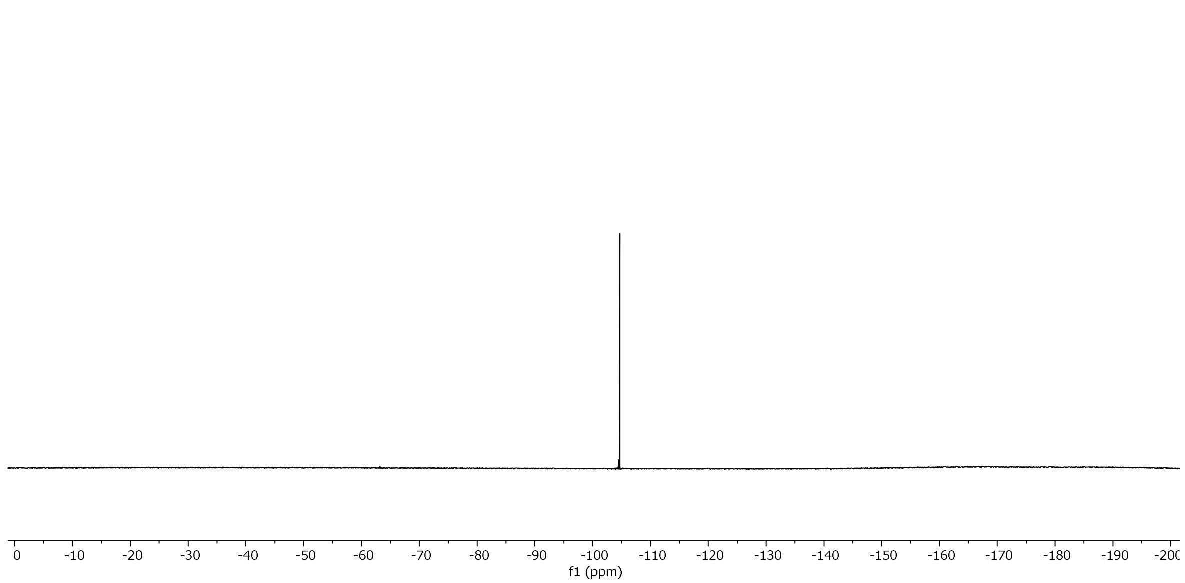

^{1}H NMR (300 MHz, Chloroform-*d*)

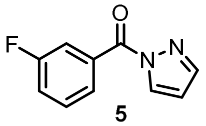

5

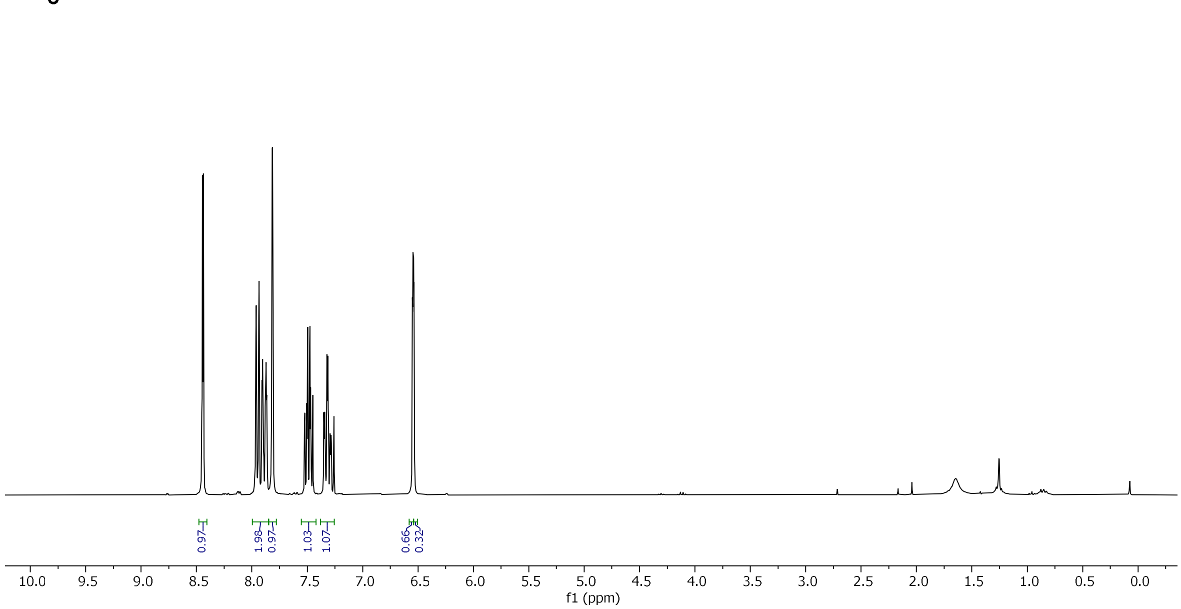

^{13}C NMR (101 MHz, Chloroform-*d*)

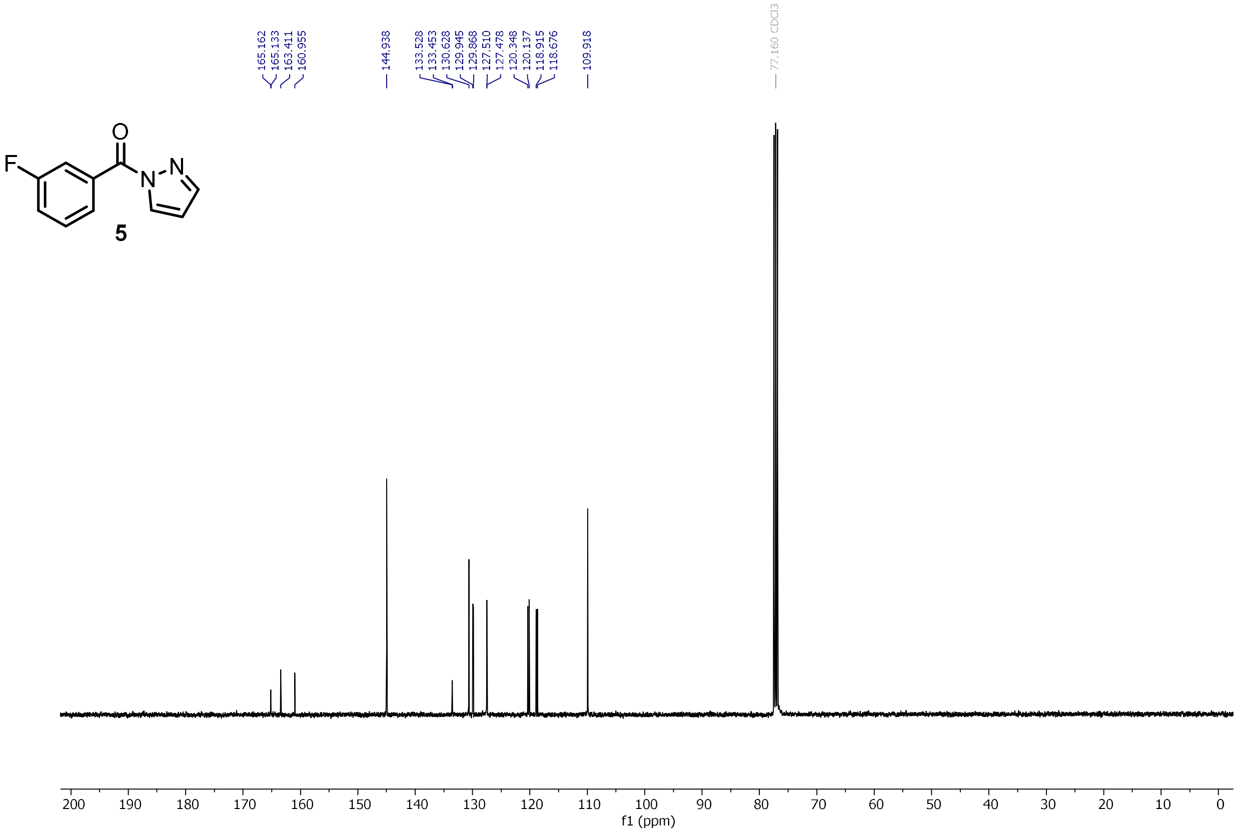

^{19}F NMR (376 MHz, Chloroform-*d*)

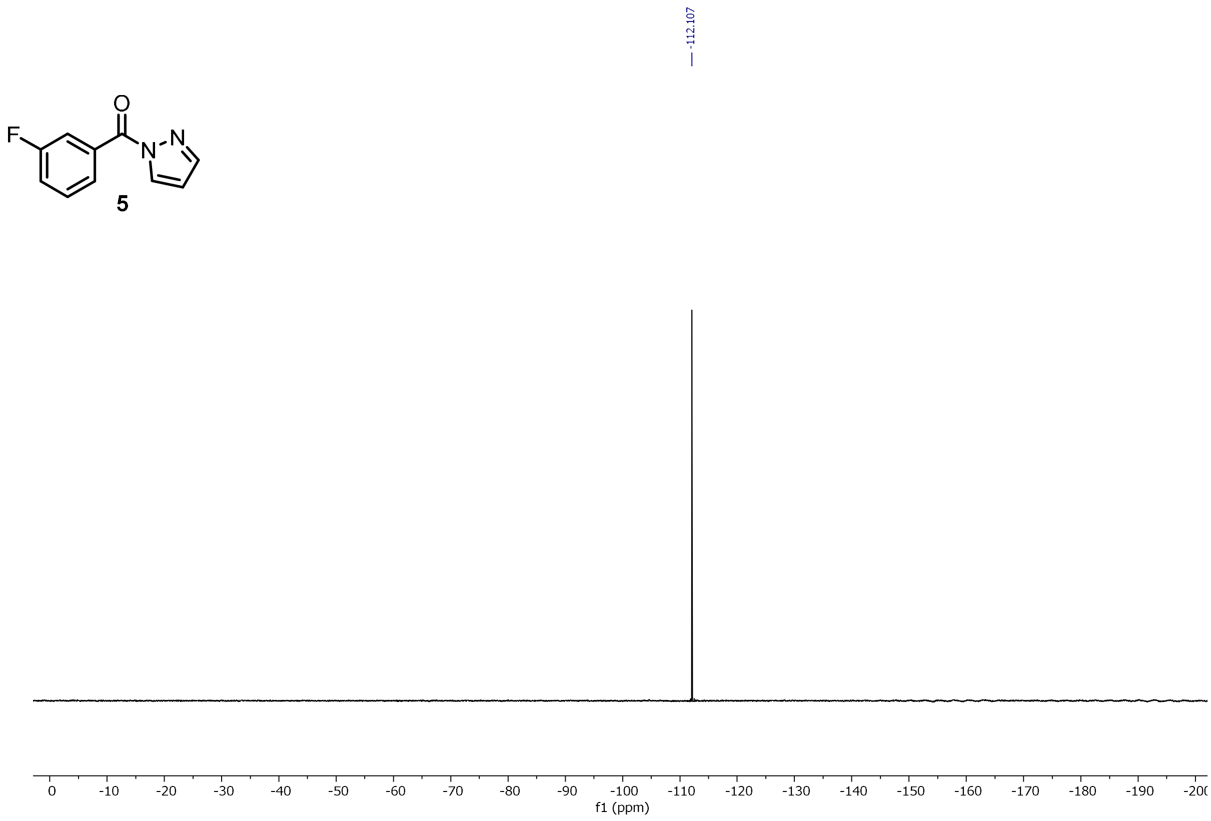

^{1}H NMR (400 MHz, Chloroform-*d*)

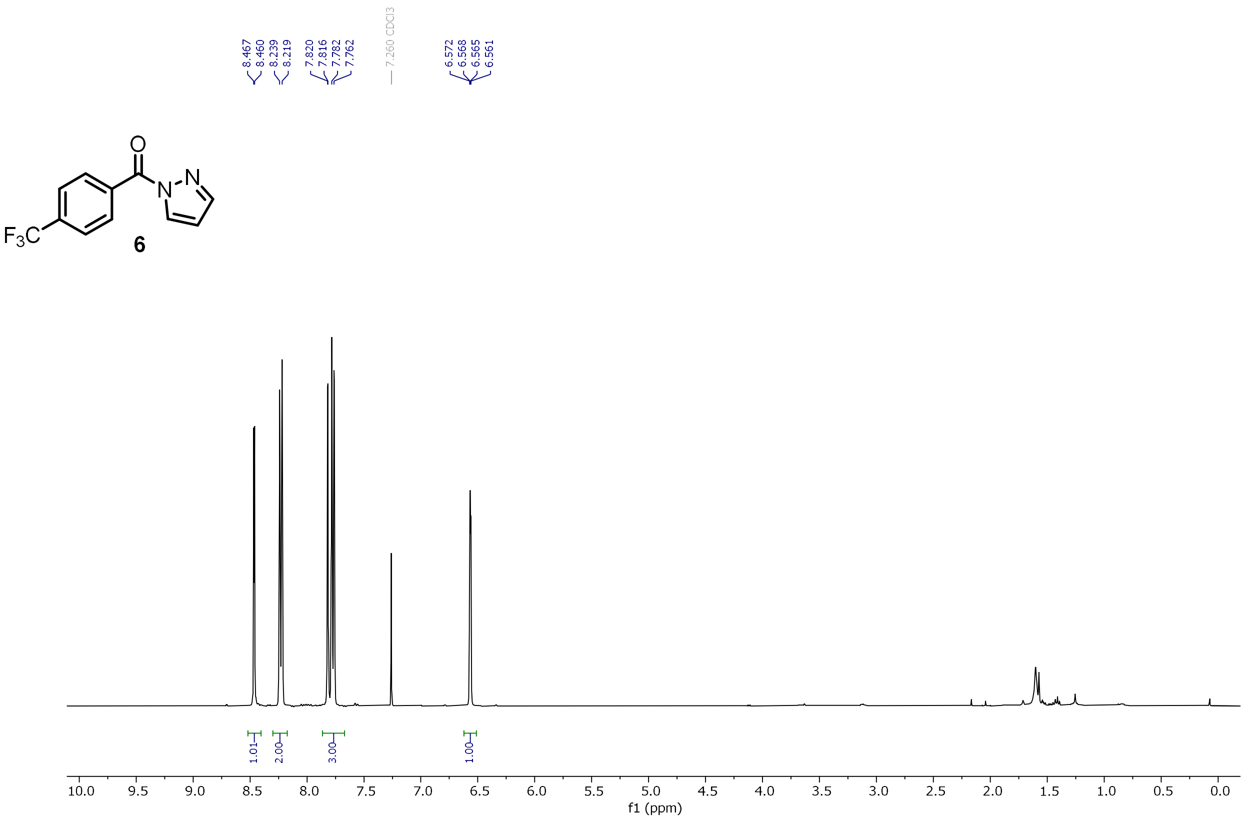

^{13}C NMR (101 MHz, Chloroform-*d*)

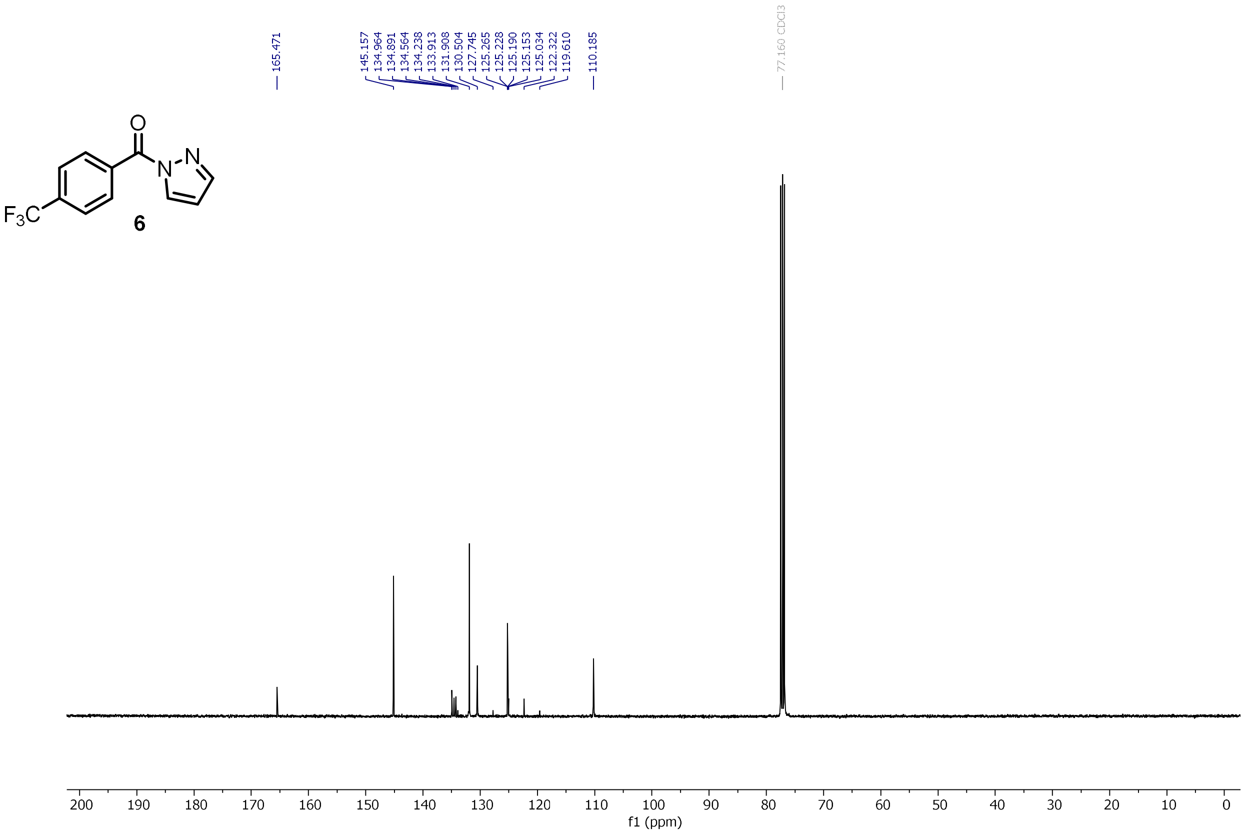

^{19}F NMR (282 MHz, Chloroform-*d*)

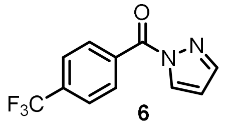

6

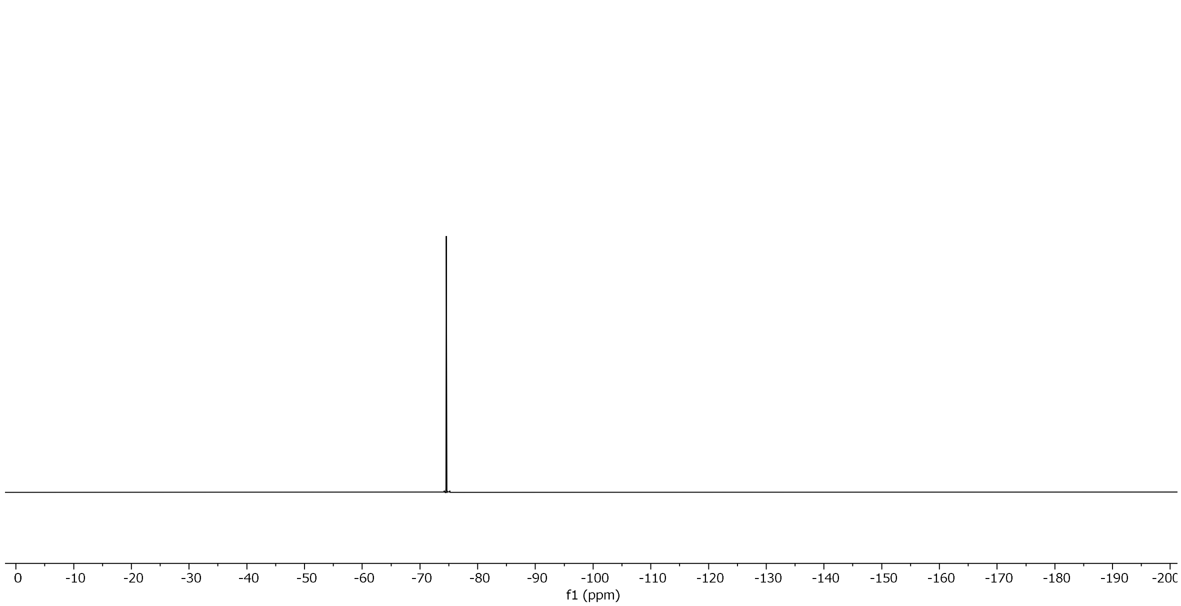

^{1}H NMR (300 MHz, Chloroform-*d*)

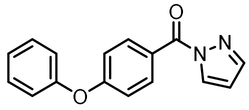

7

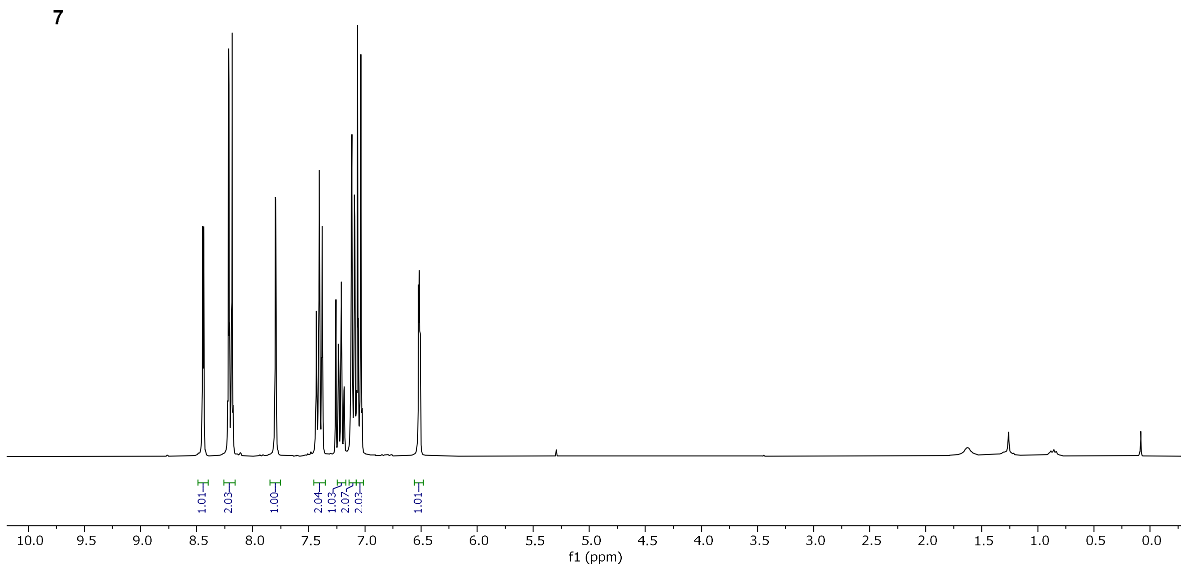

^{13}C NMR (75 MHz, Chloroform-*d*)

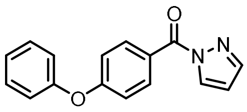

7

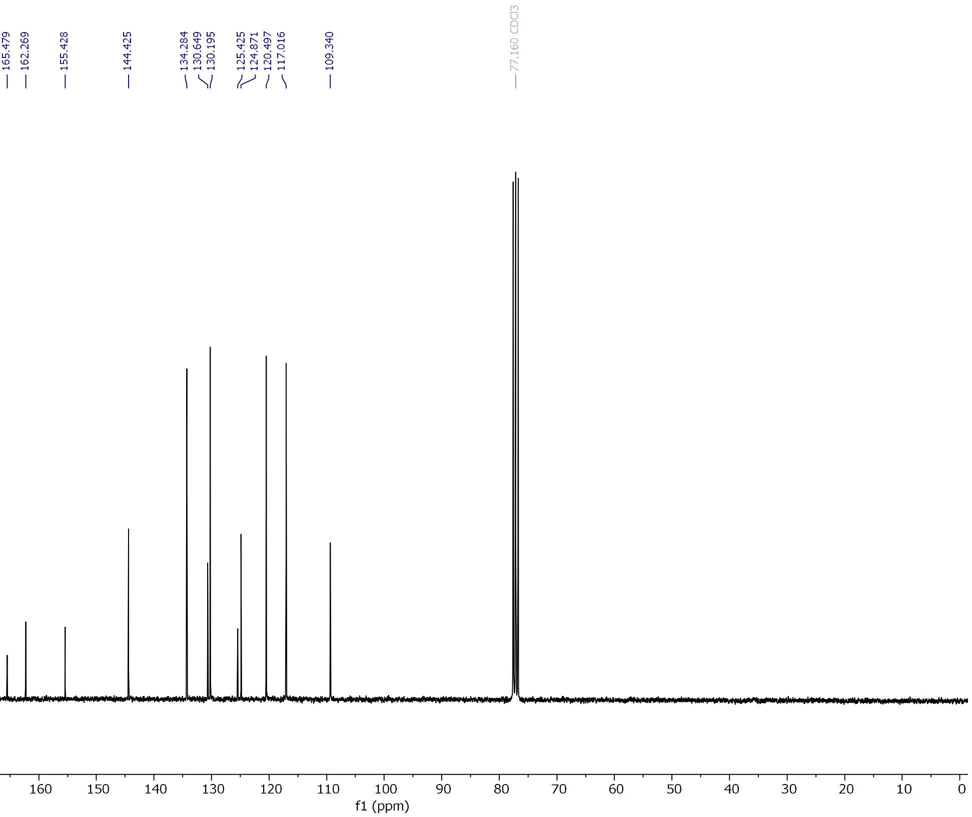

^{1}H NMR (400 MHz, Chloroform-*d*)

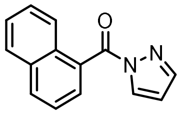

8

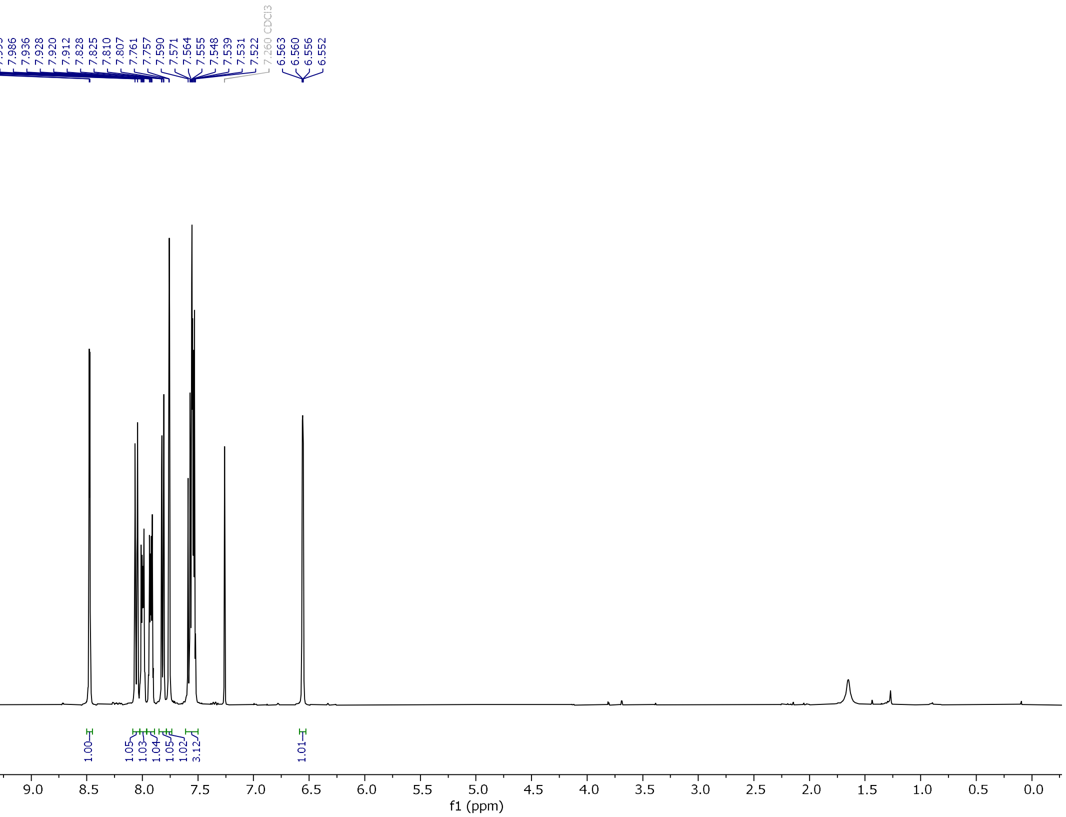

^{13}C NMR (101 MHz, Chloroform-*d*)

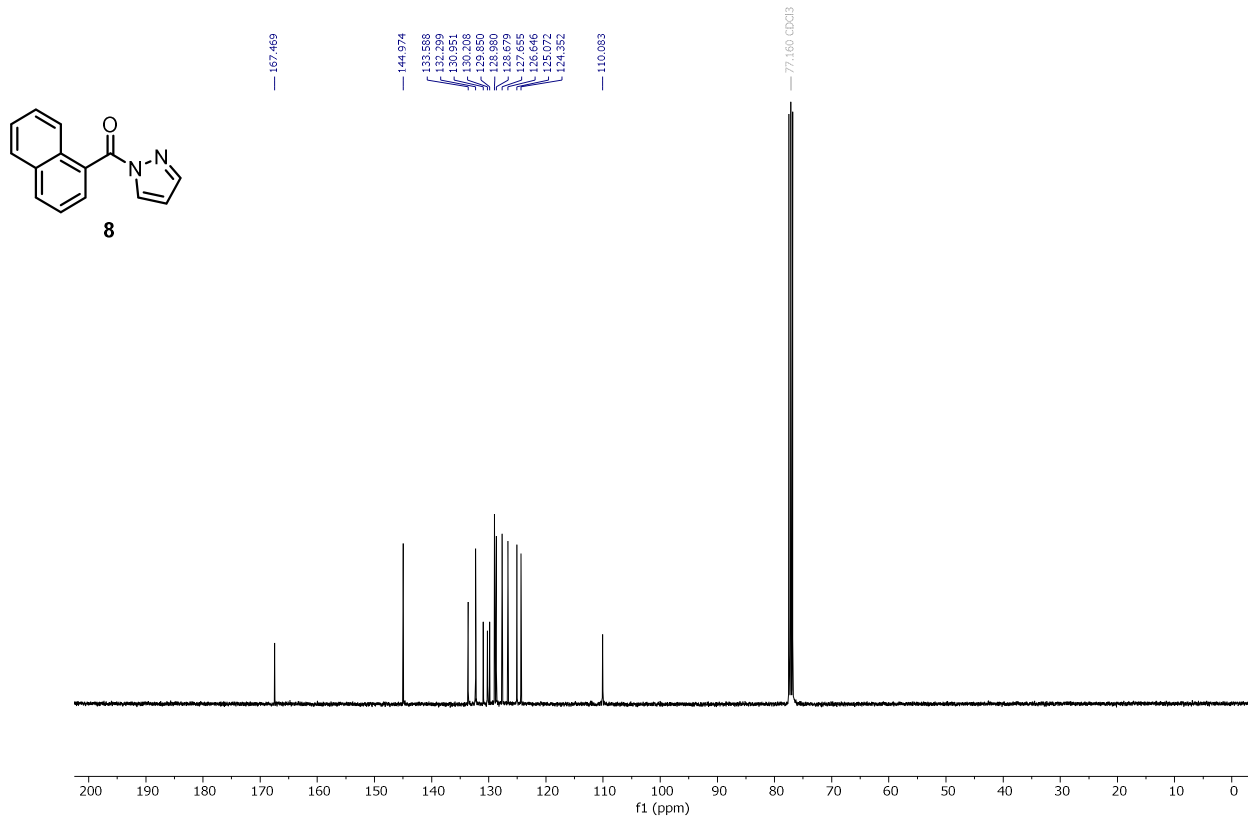

8

^{1}H NMR (400 MHz, Chloroform-*d*)

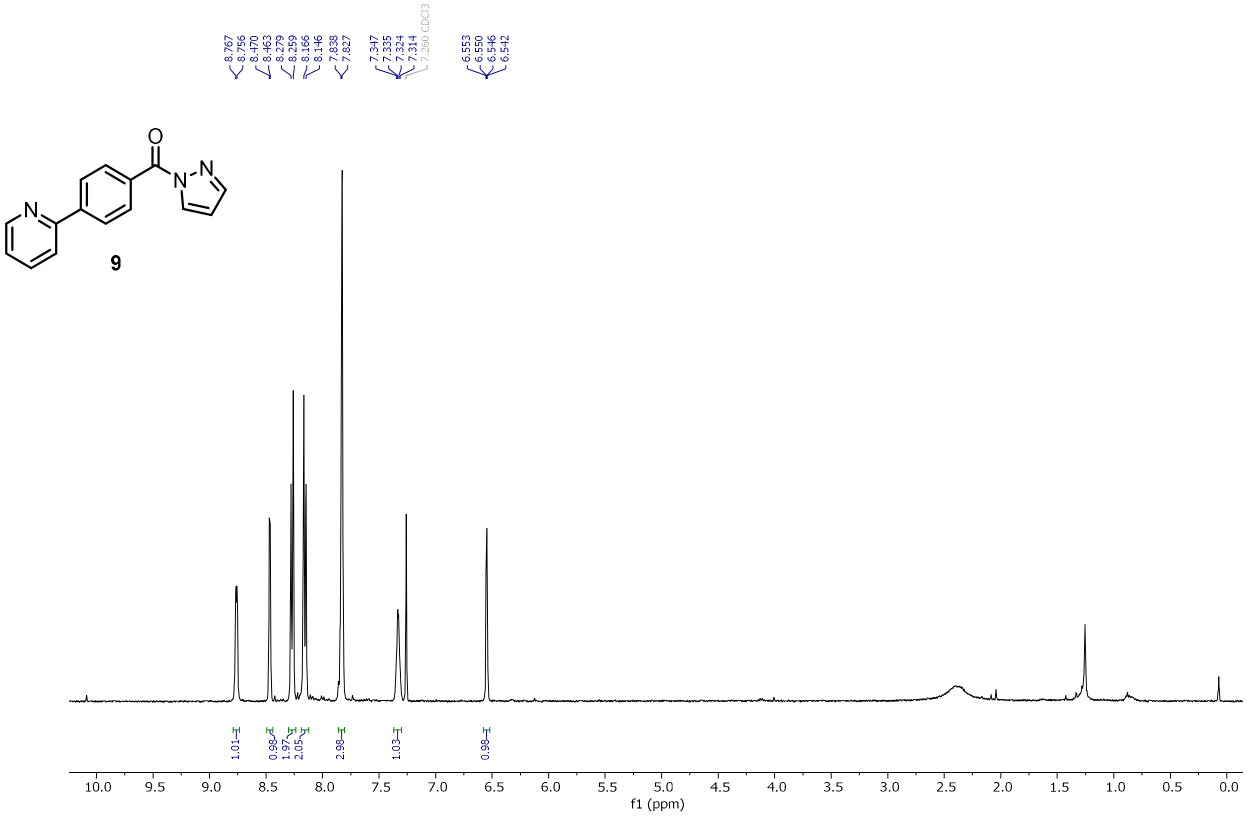

9

^{13}C NMR (101 MHz, Chloroform-*d*)

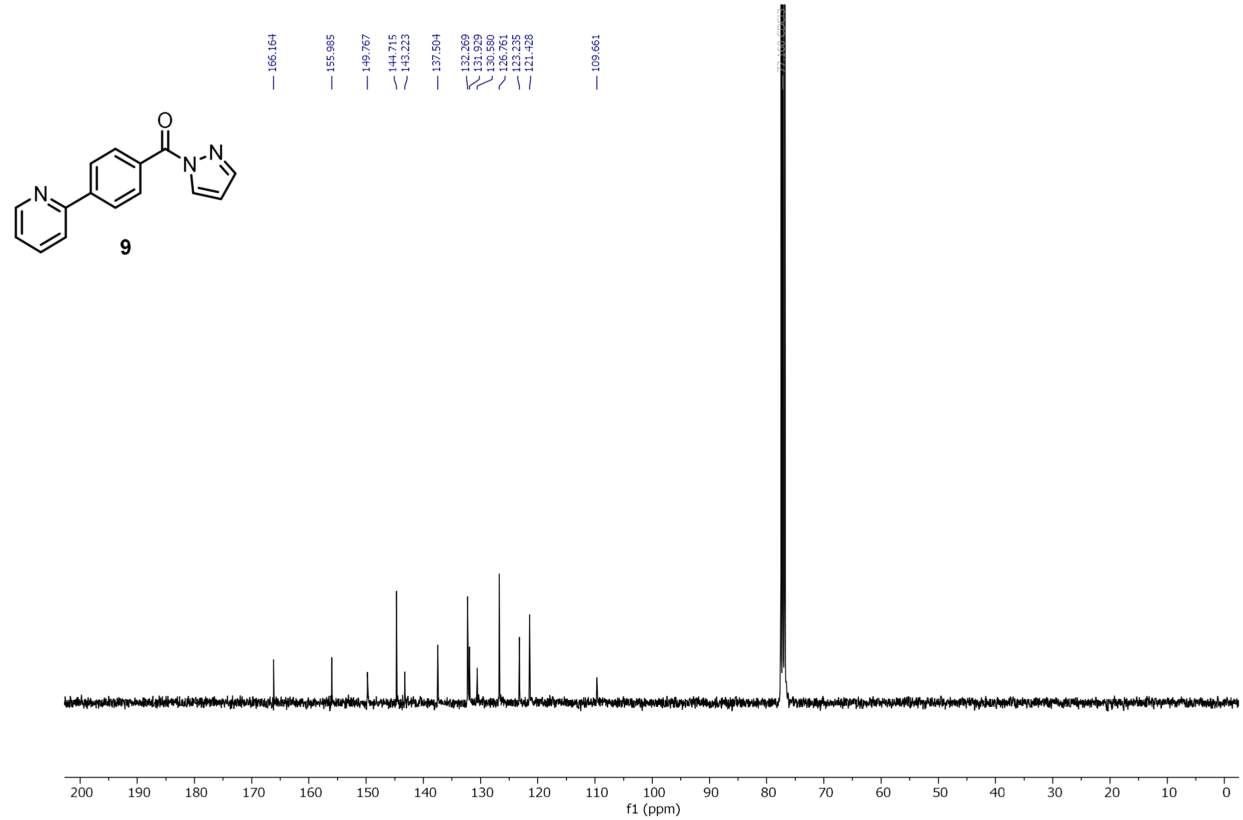

^{1}H NMR (300 MHz, Chloroform-*d*)

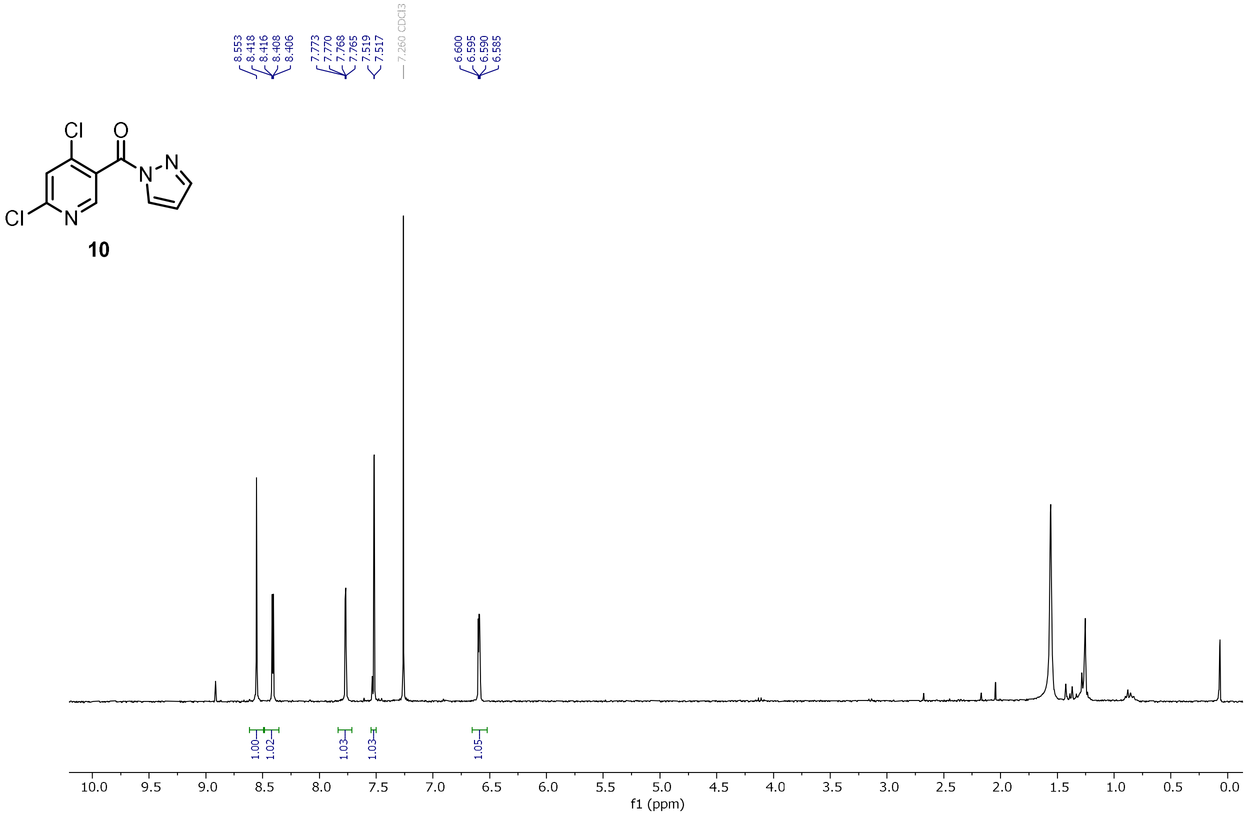

^{13}C NMR (101 MHz, Chloroform-d)

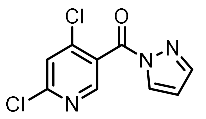

10

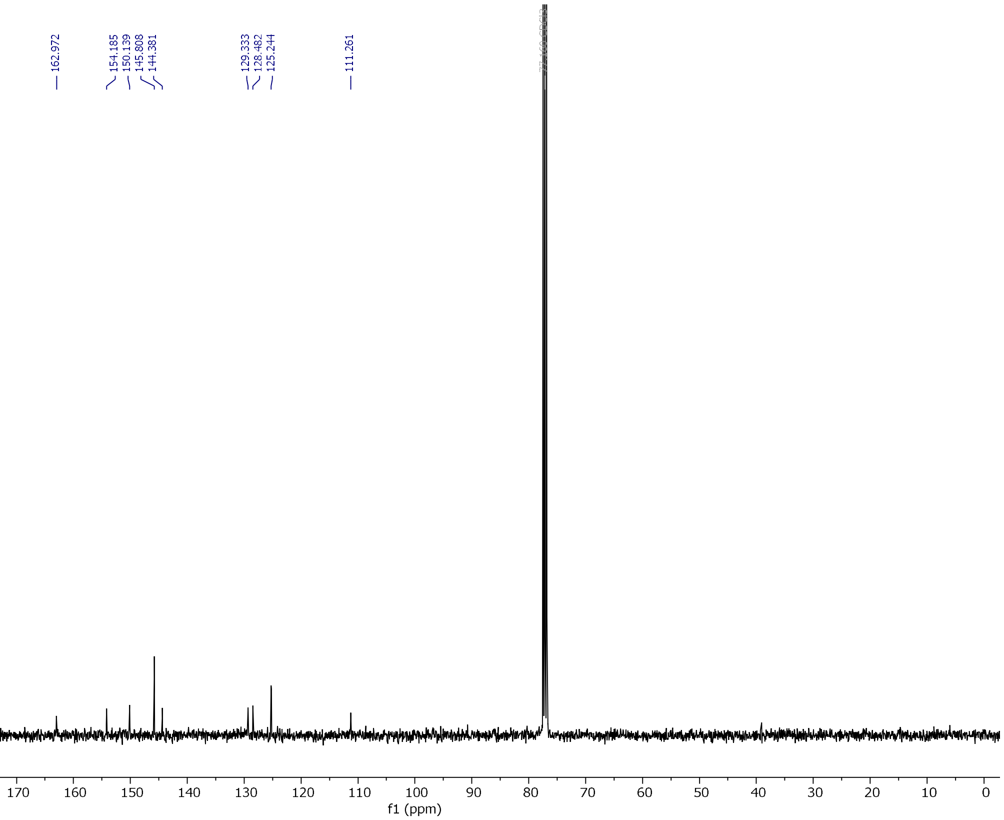

^{1}H NMR (400 MHz, Chloroform-d)

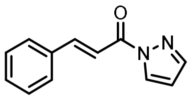

11

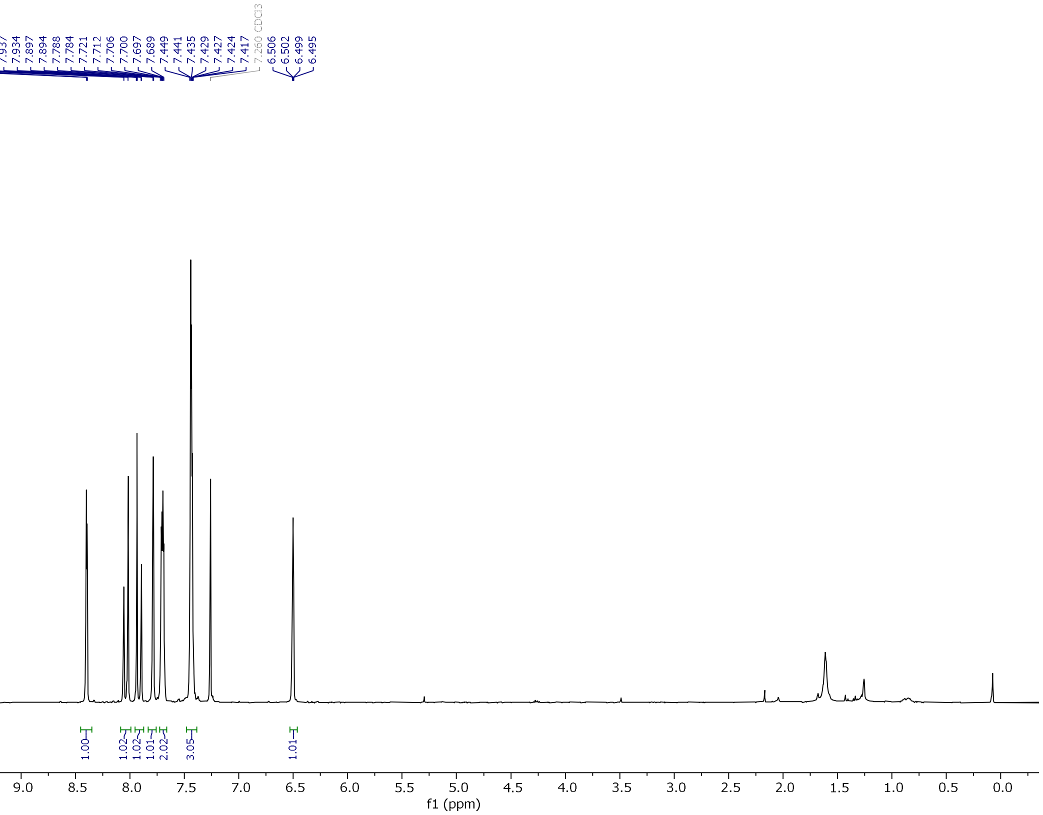

^{13}C NMR (101 MHz, Chloroform-*d*)

11

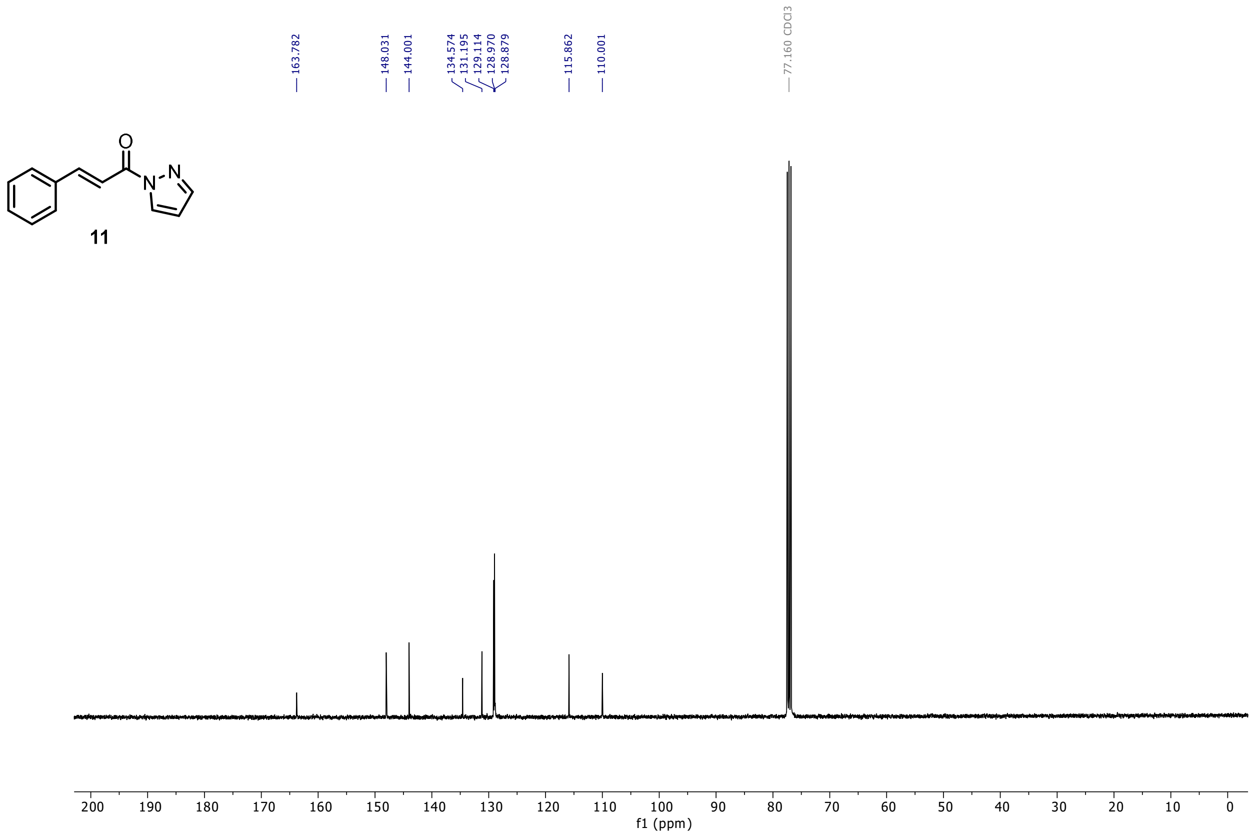

^{1}H NMR (400 MHz, Chloroform-*d*)

12

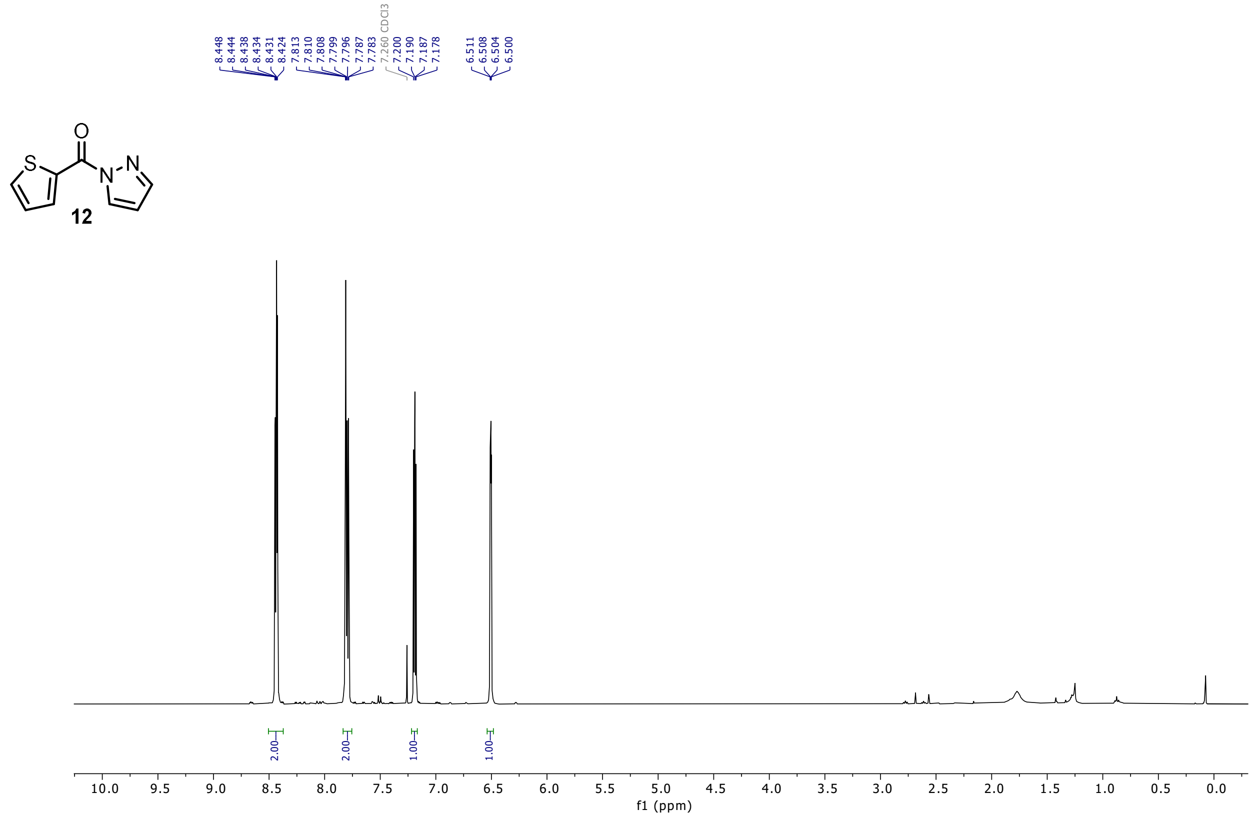

13C NMR (101 MHz, Chloroform-*d*)

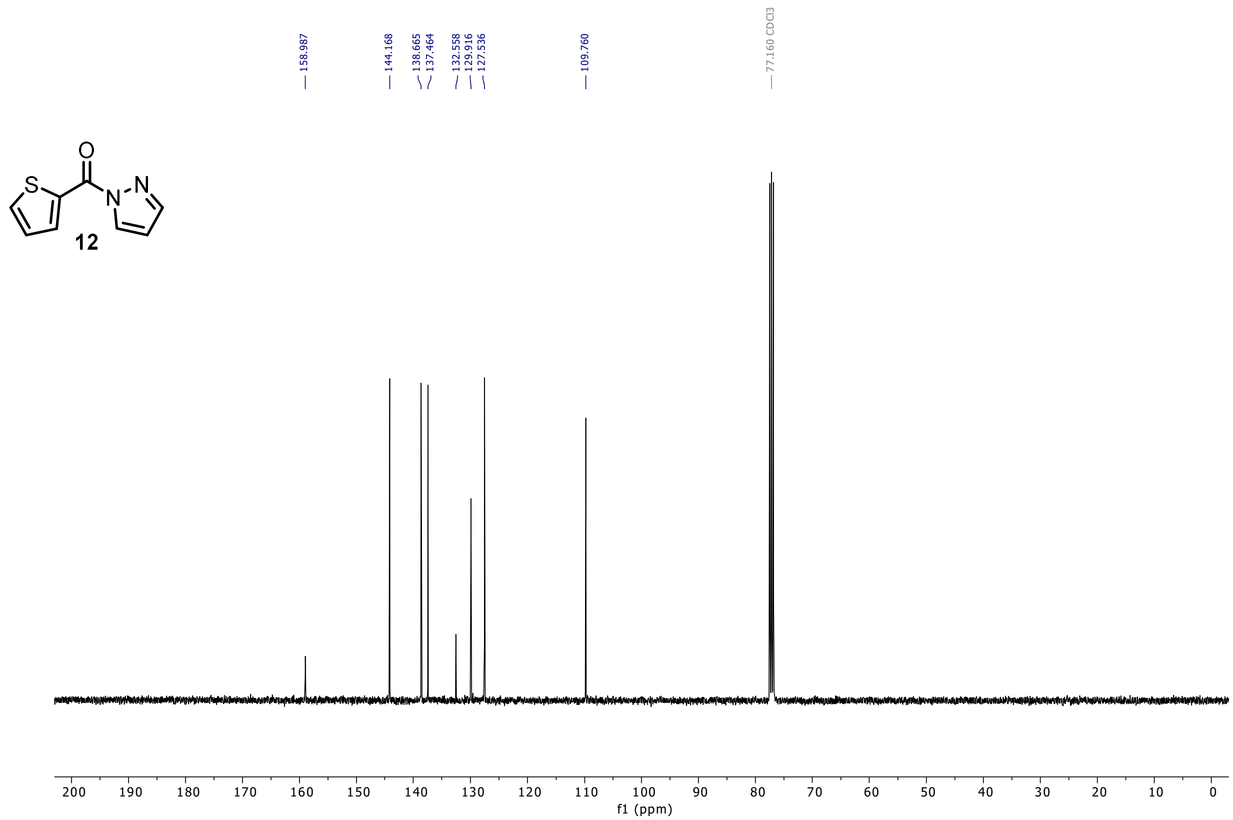

1H NMR (300 MHz, Chloroform-*d*)

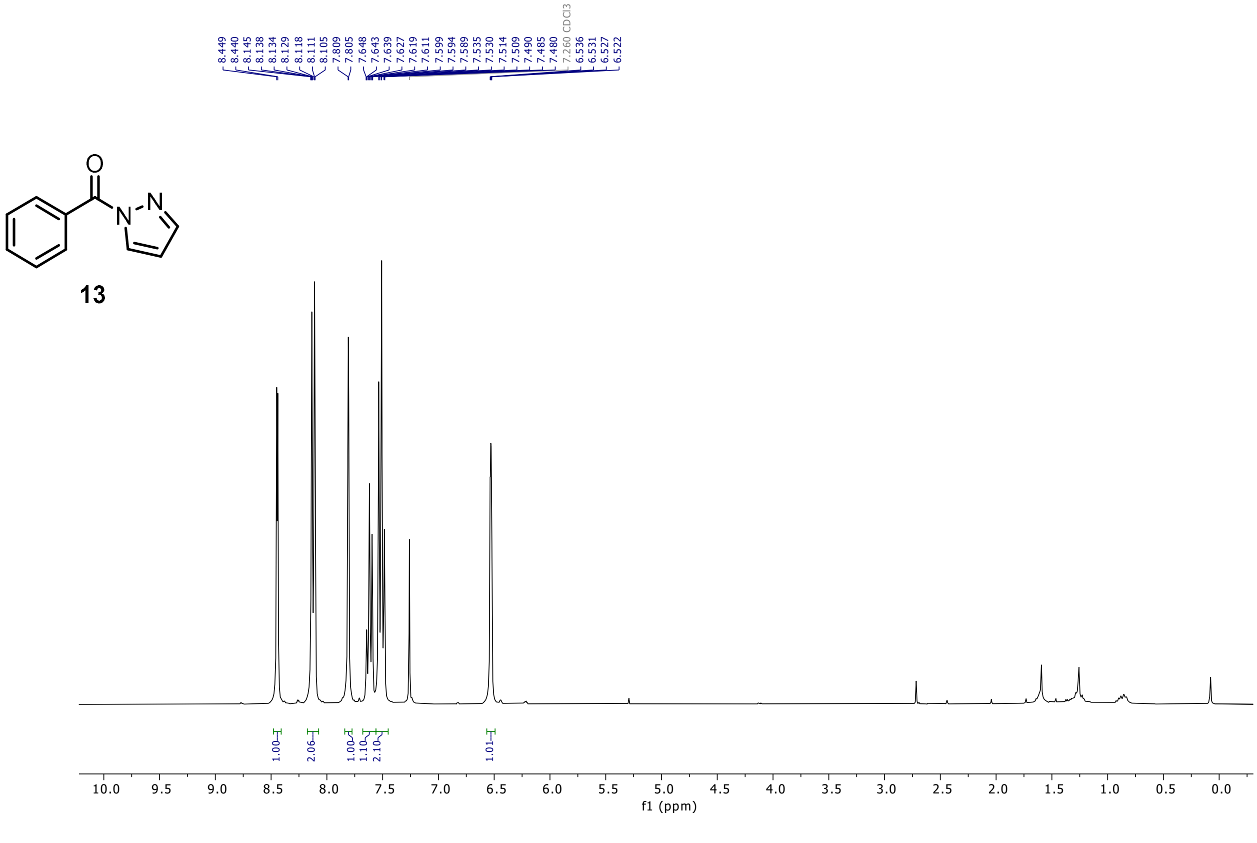

^{13}C NMR (75 MHz, Chloroform-*d*)

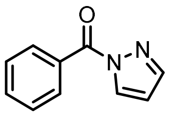

13

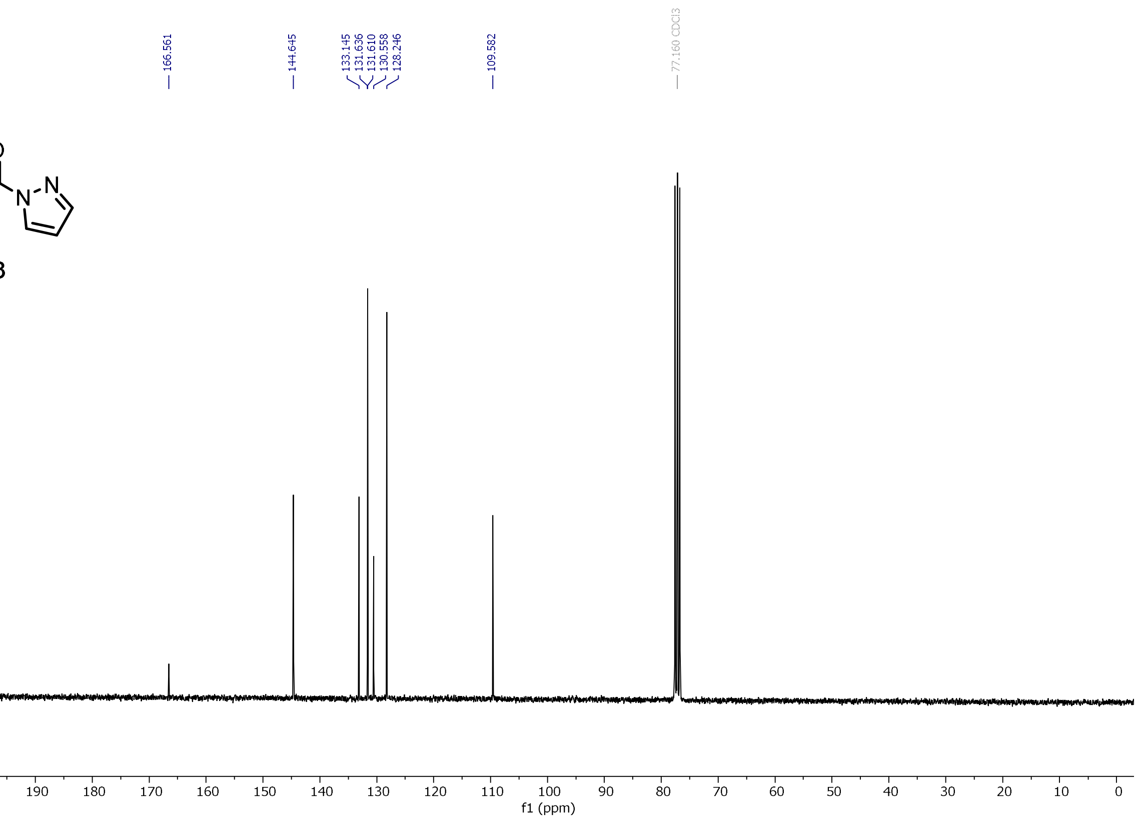

^{1}H NMR (300 MHz, Chloroform-*d*)

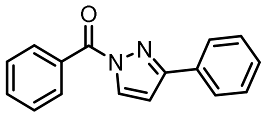

14

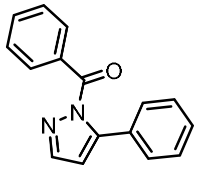

14'

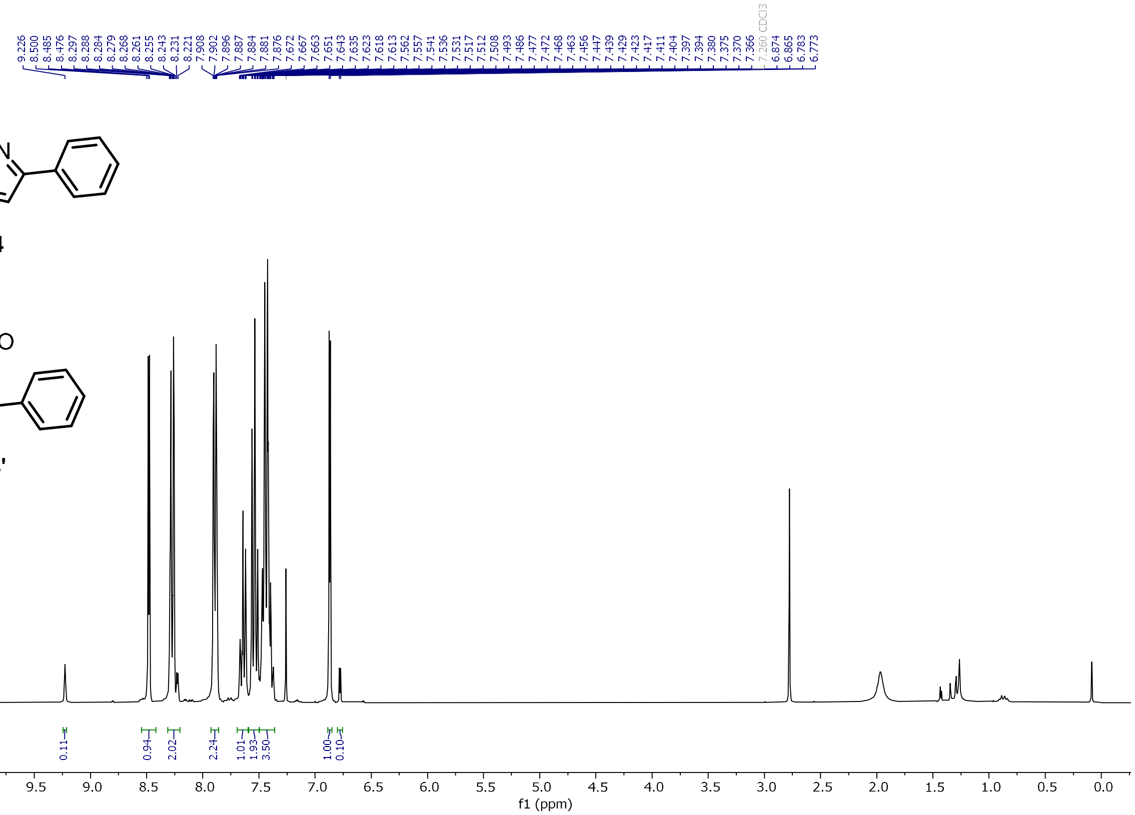

^{13}C NMR (101 MHz, Chloroform-*d*)

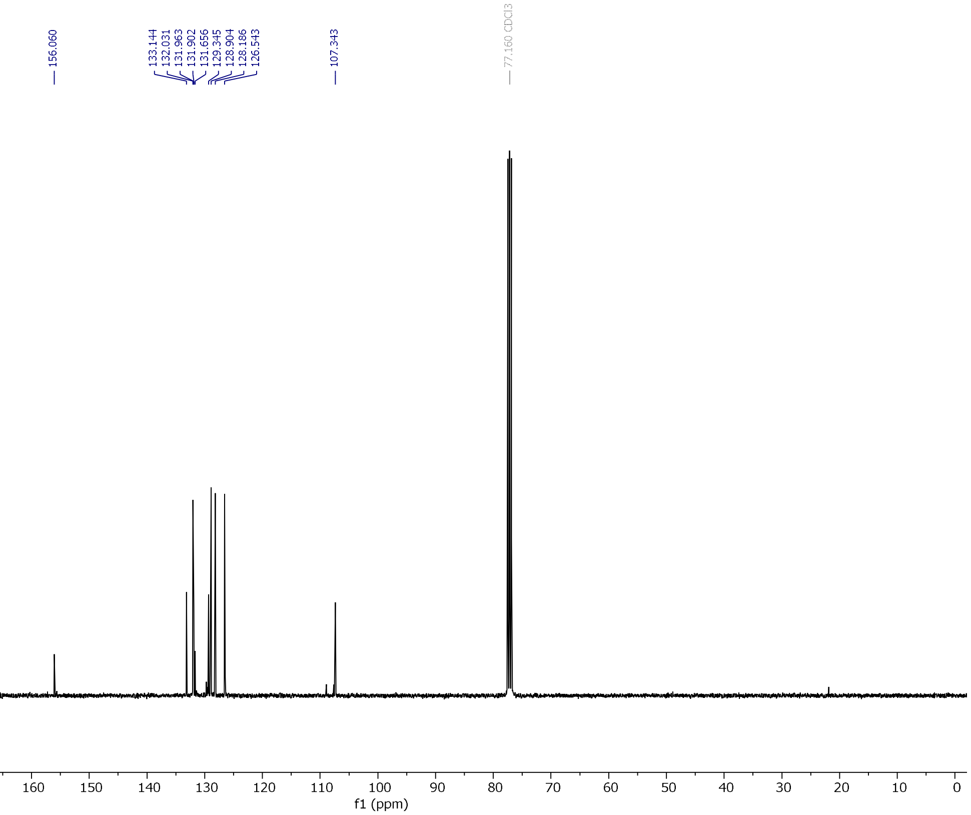

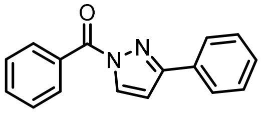

14

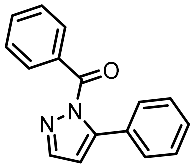

14'

^{1}H NMR (300 MHz, Chloroform-*d*)

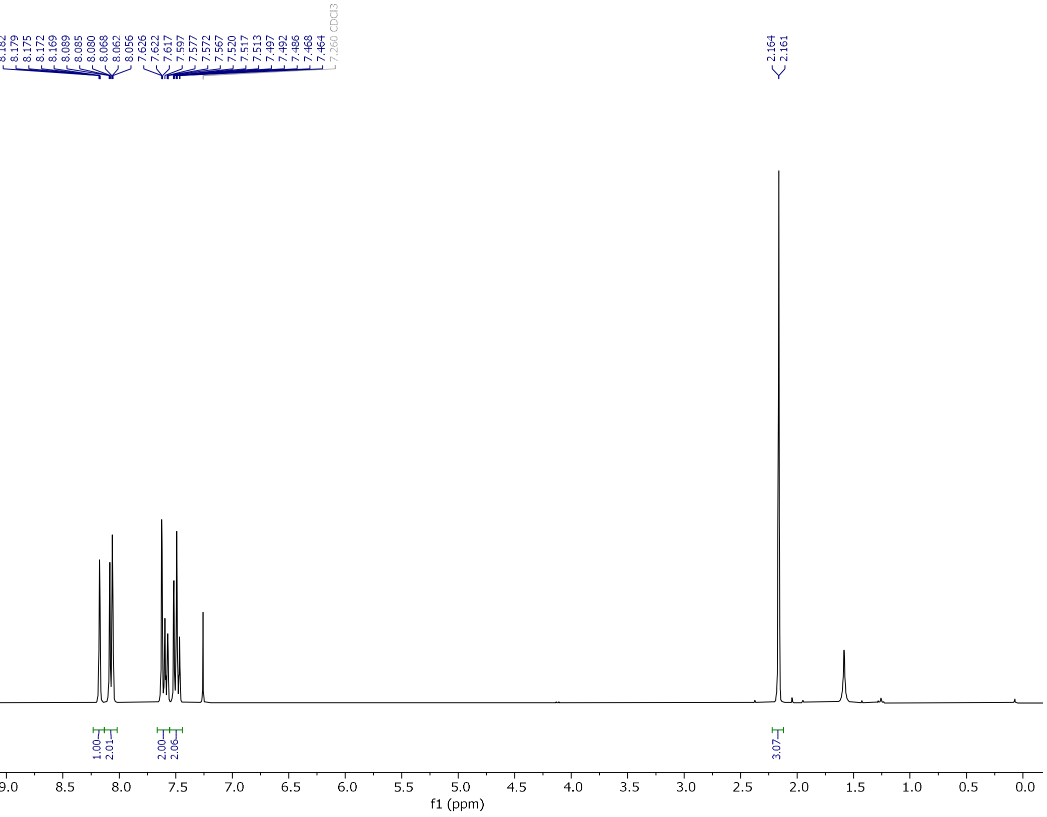

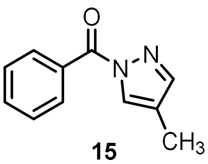

15

^{13}C NMR (75 MHz, Chloroform-*d*)

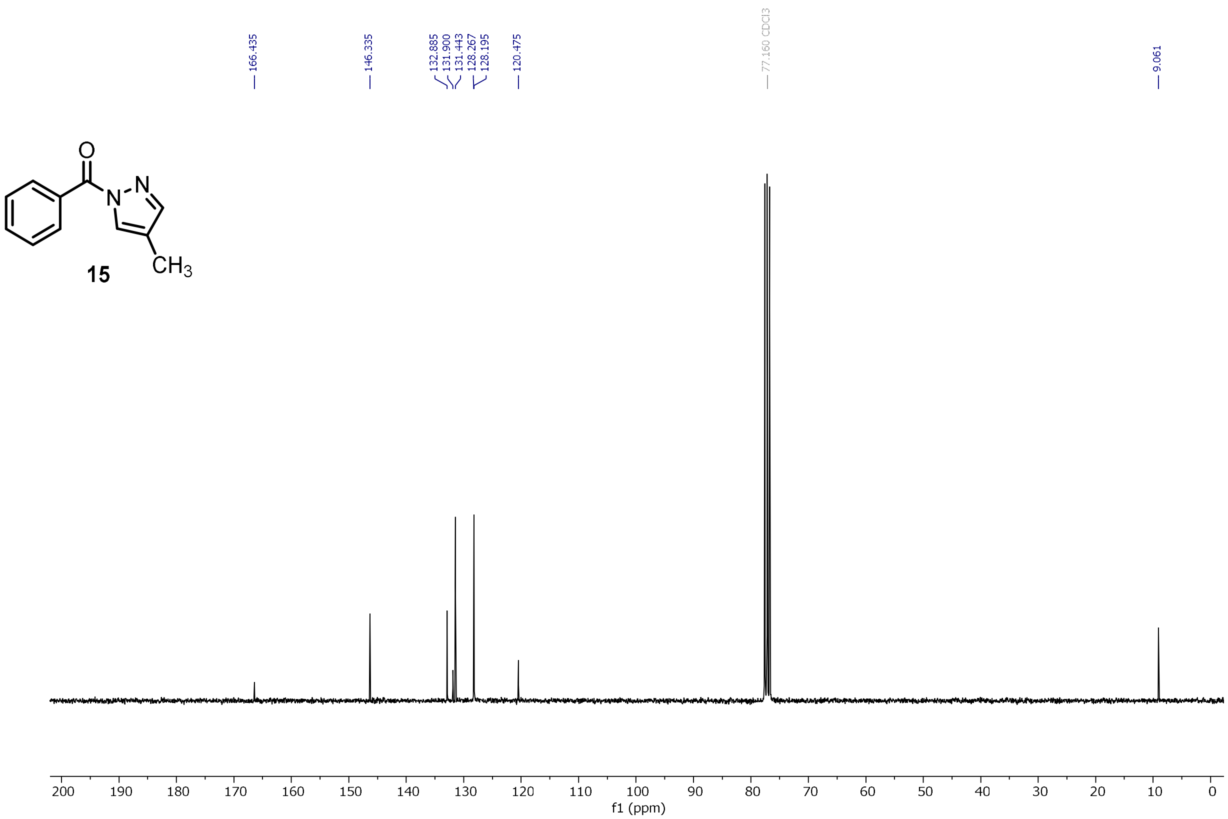

15

^{1}H NMR (400 MHz, Chloroform-*d*)

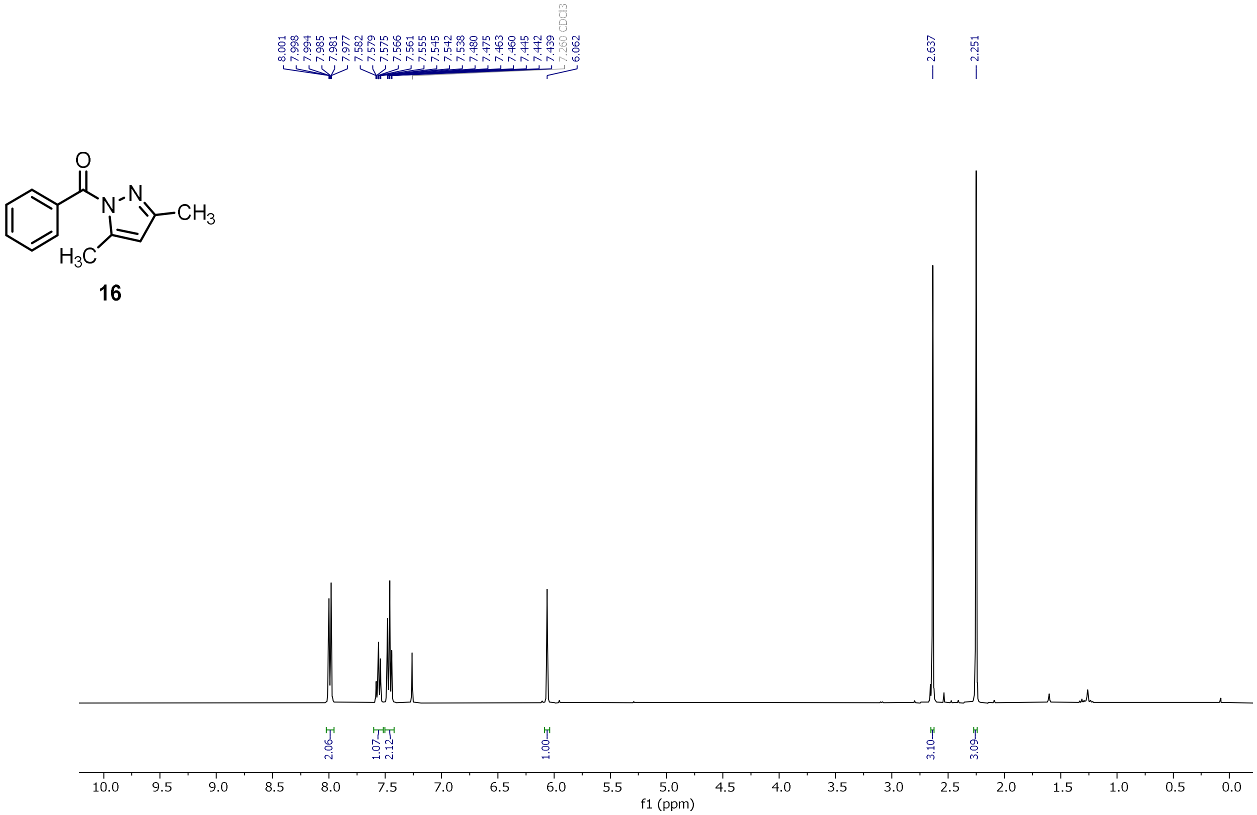

16

^{13}C NMR (101 MHz, Chloroform-*d*)

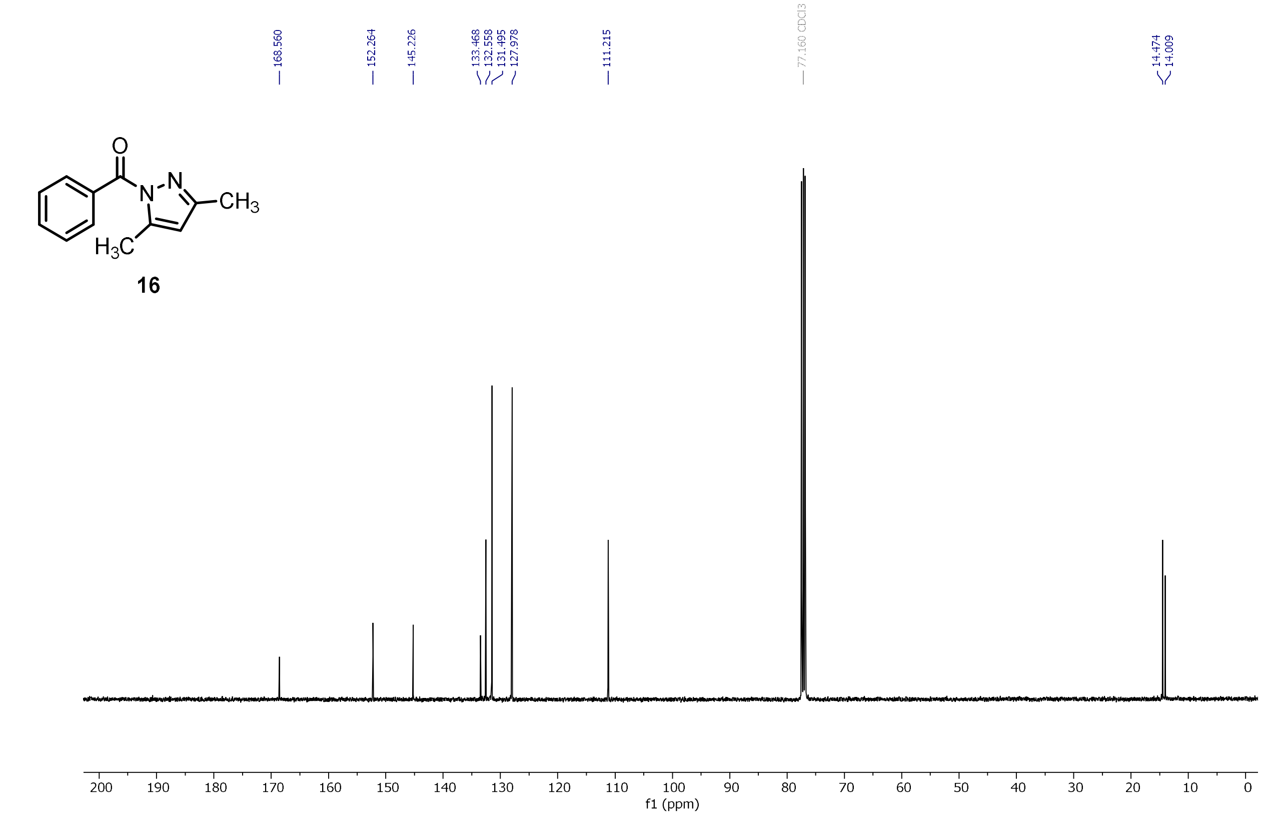

16

^{1}H NMR (300 MHz, Chloroform-*d*)

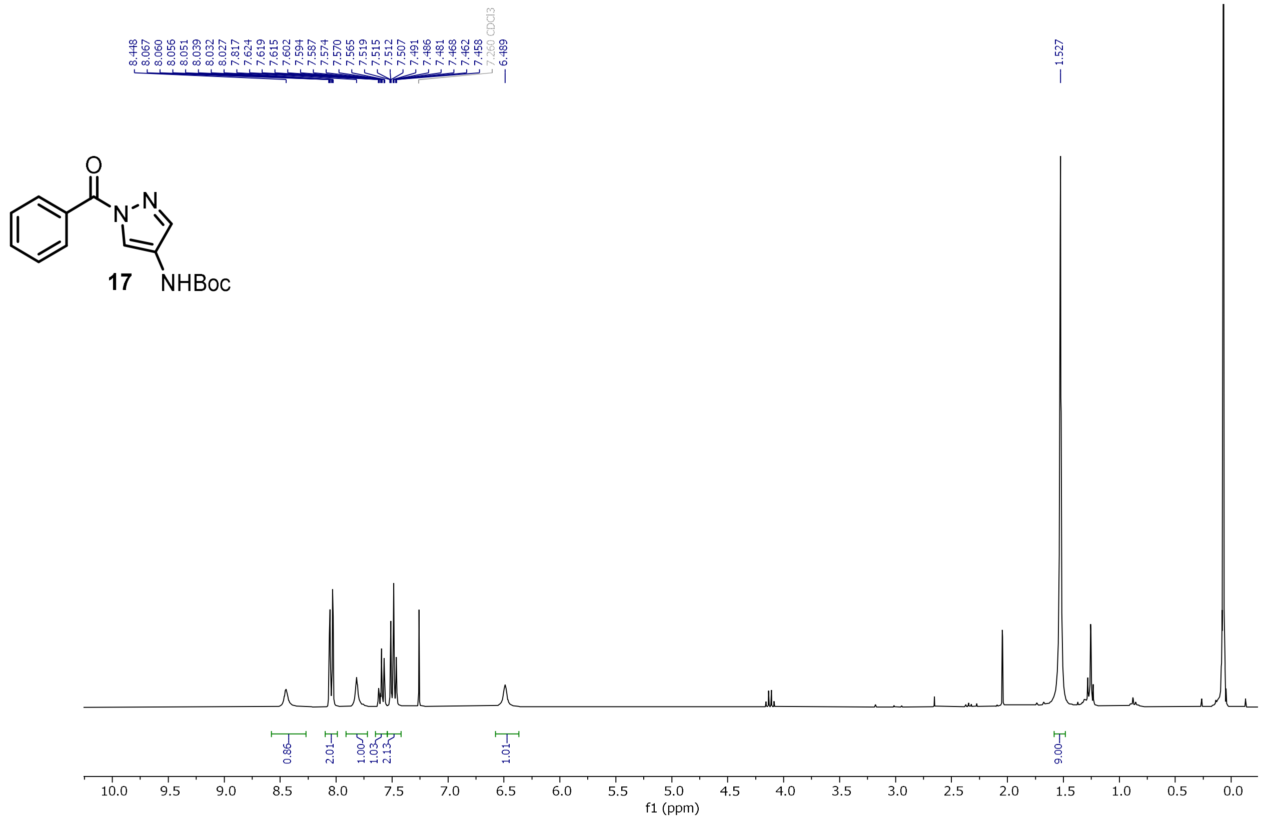

17

^{13}C NMR (101 MHz, Chloroform-*d*)

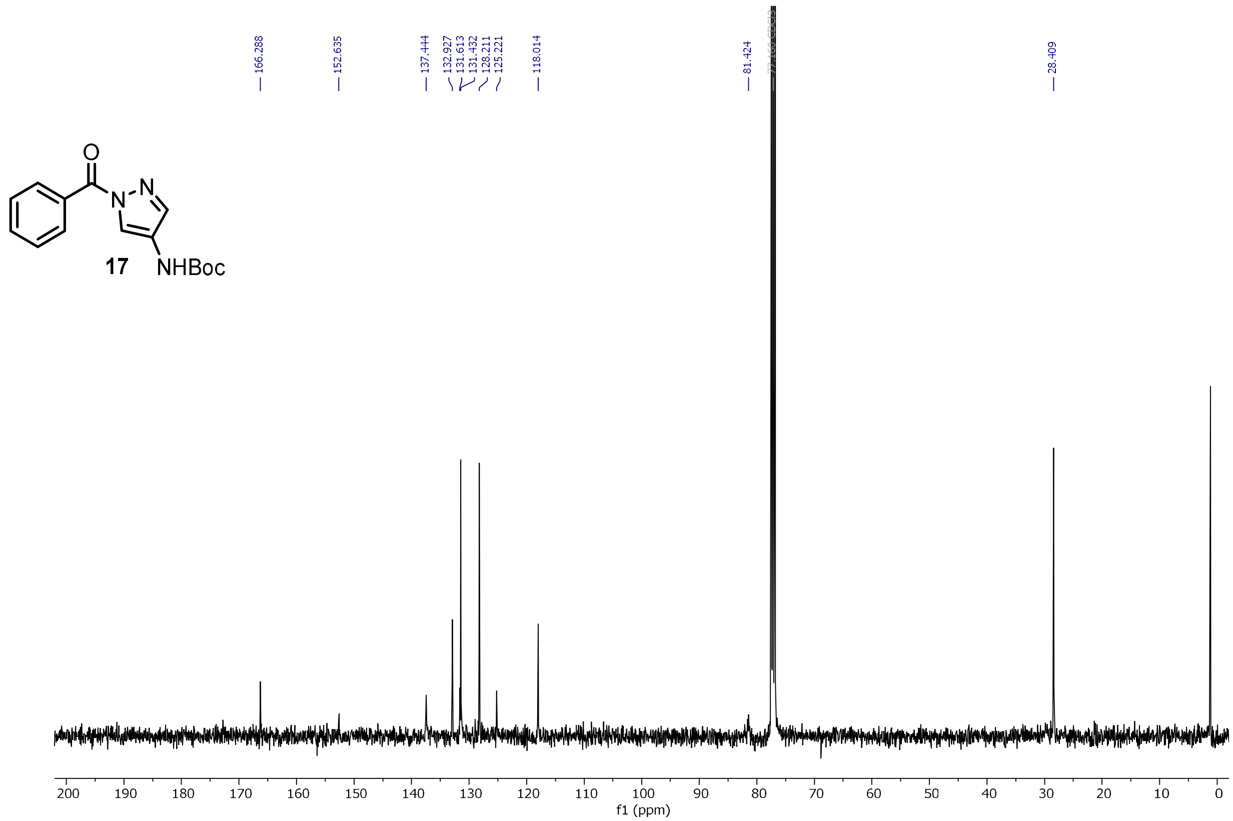

^{1}H NMR (300 MHz, Chloroform-*d*)

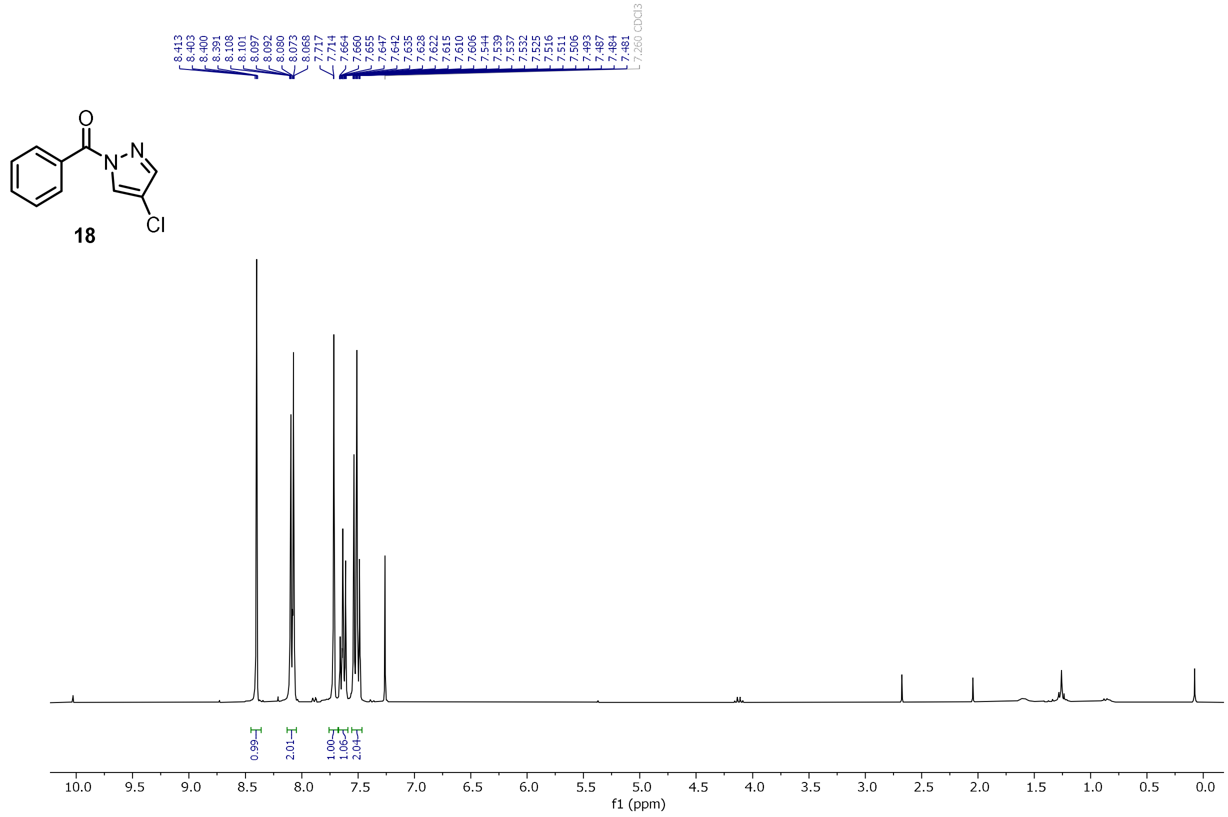

^{13}C NMR (75 MHz, Chloroform-*d*)

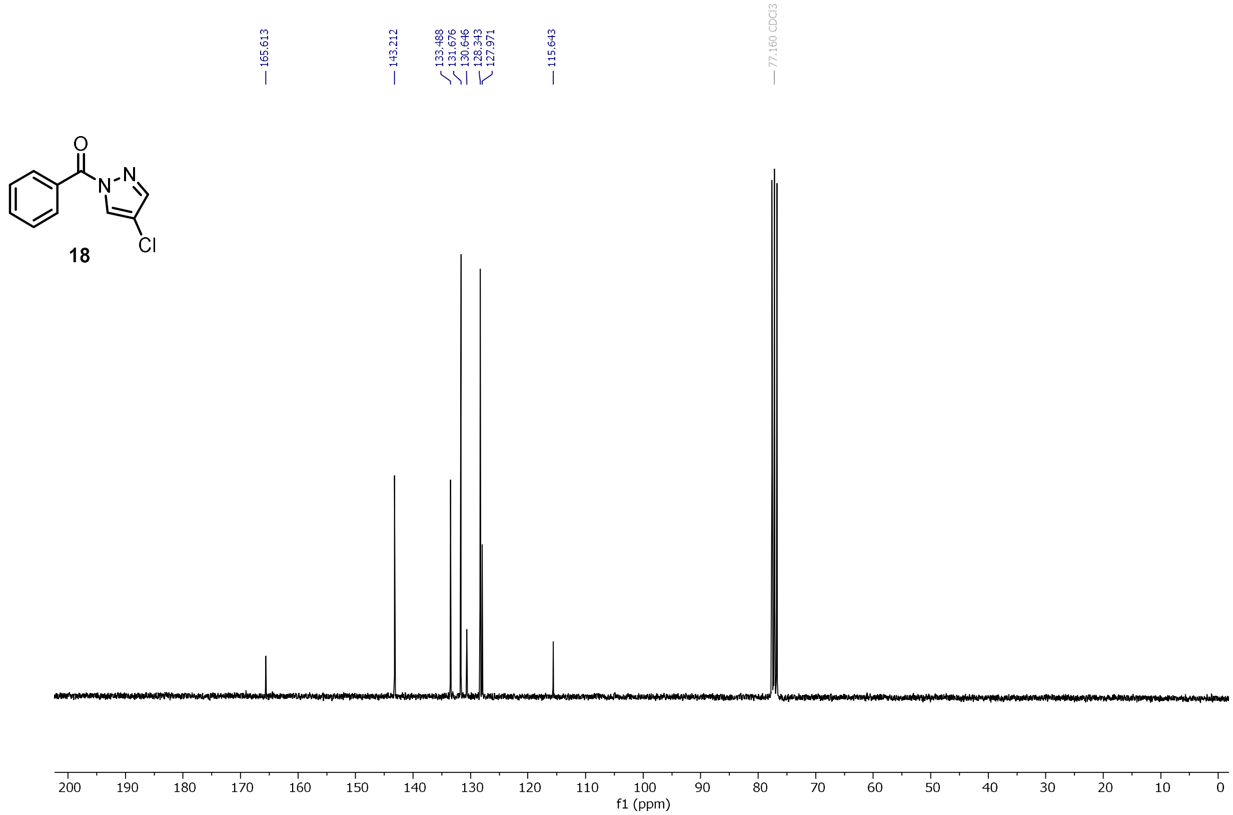

^{1}H NMR (300 MHz, Chloroform-*d*)

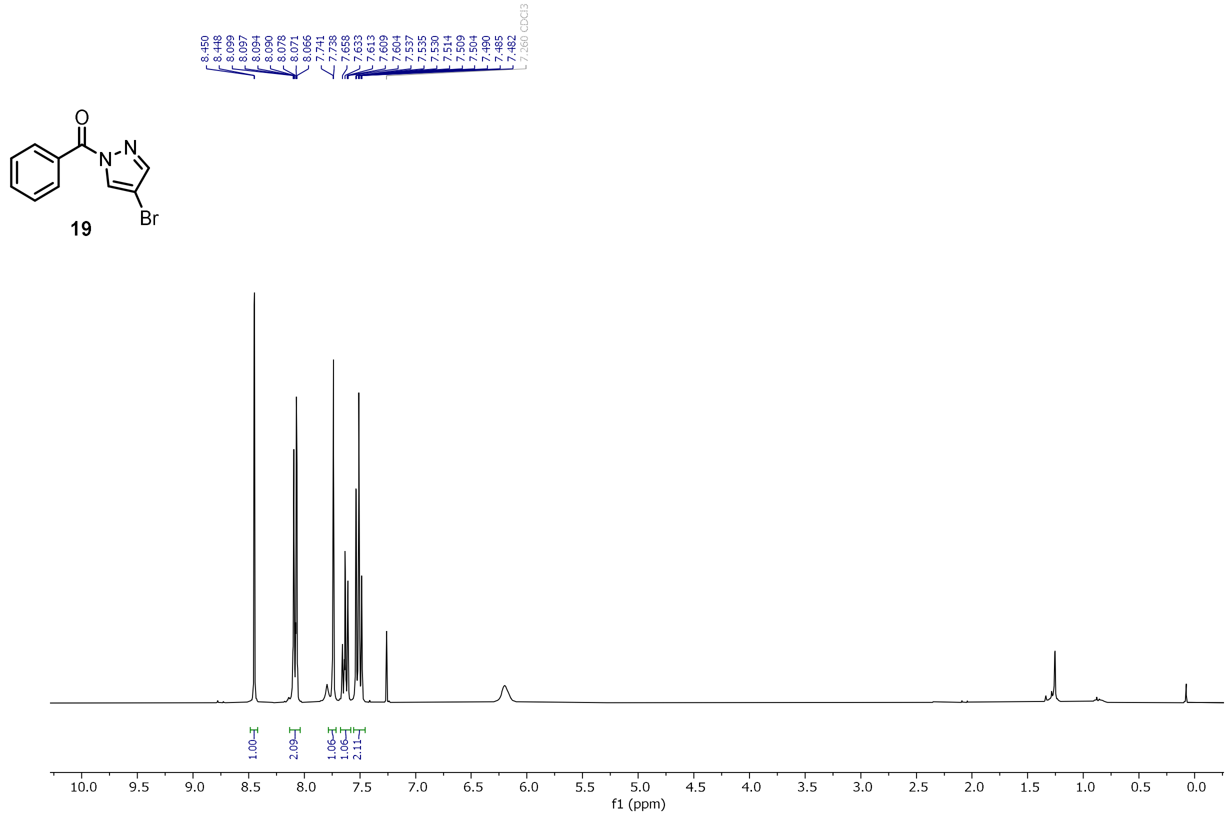

^{13}C NMR (101 MHz, Chloroform-*d*)

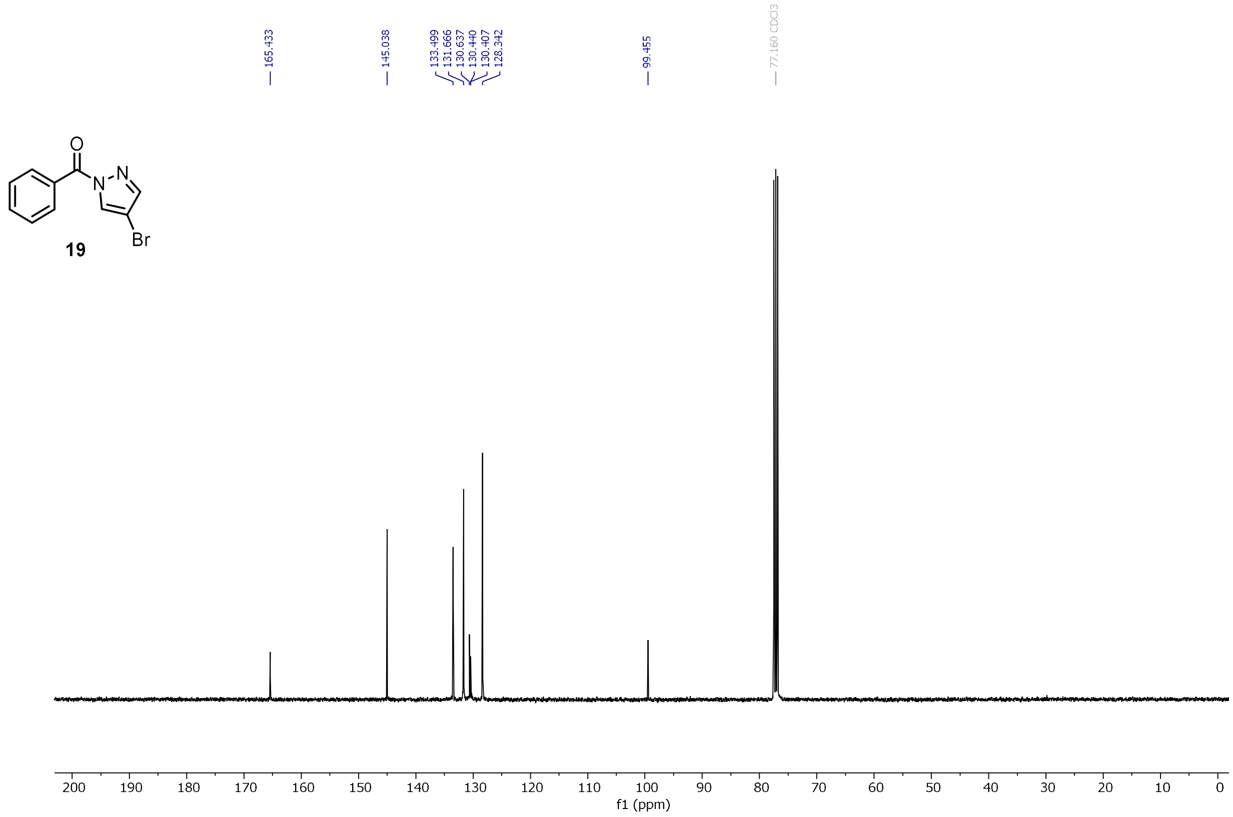

^{1}H NMR (400 MHz, Chloroform-*d*)

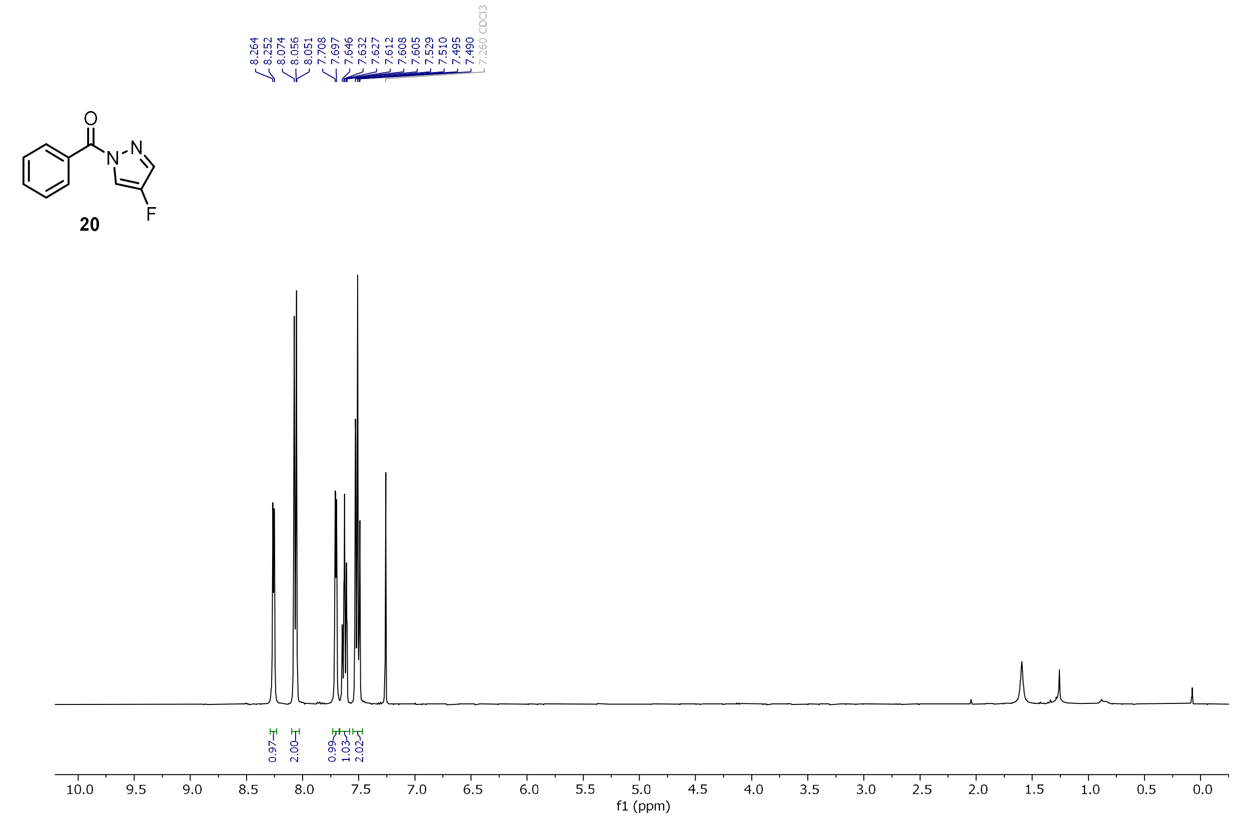

^{13}C NMR (101 MHz, Chloroform-*d*)

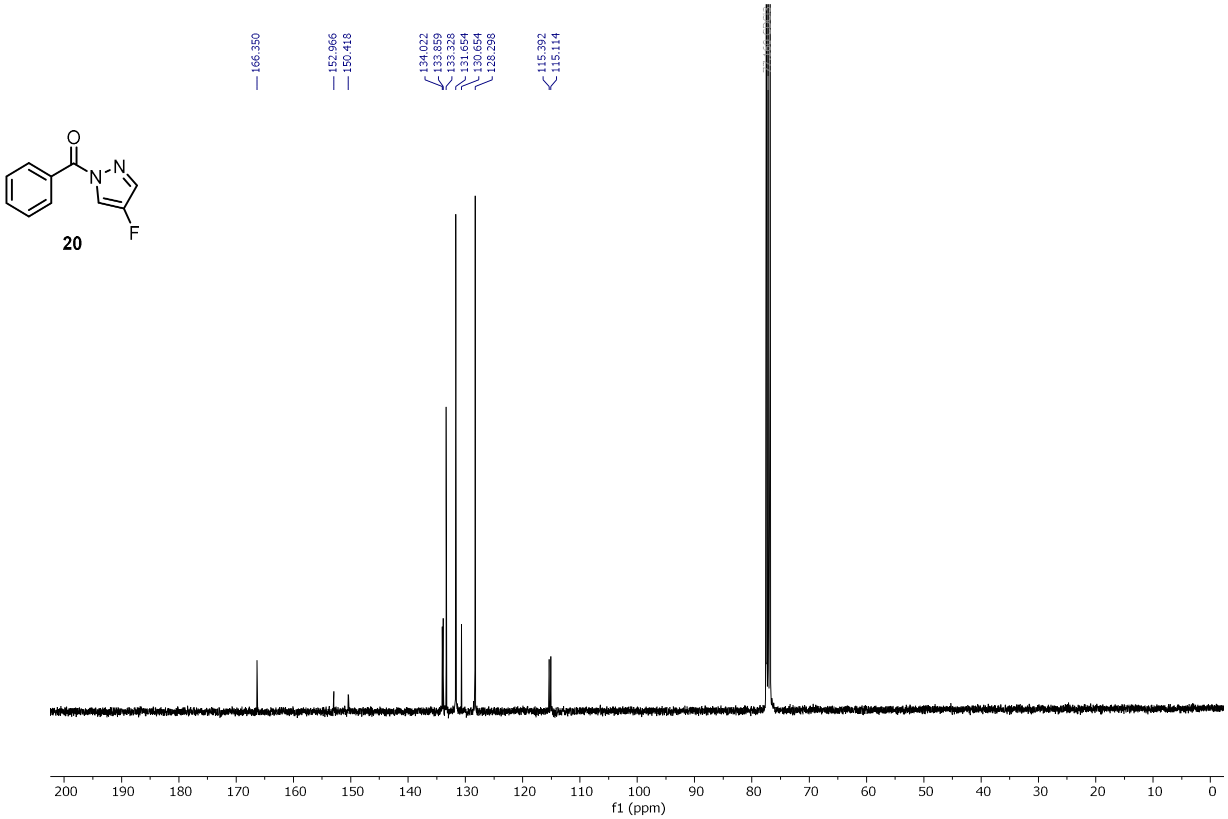

^{19}F NMR (282 MHz, Chloroform-*d*)

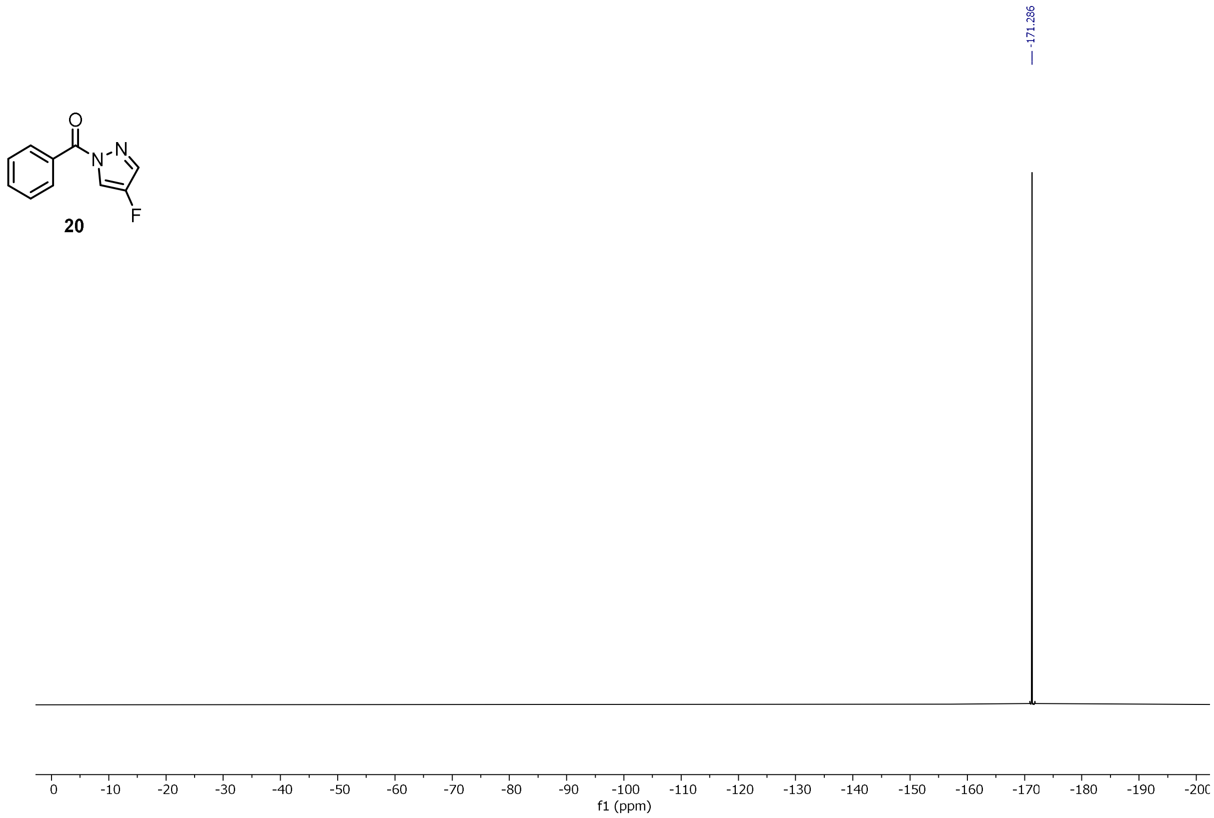

^{1}H NMR (300 MHz, Chloroform-*d*)

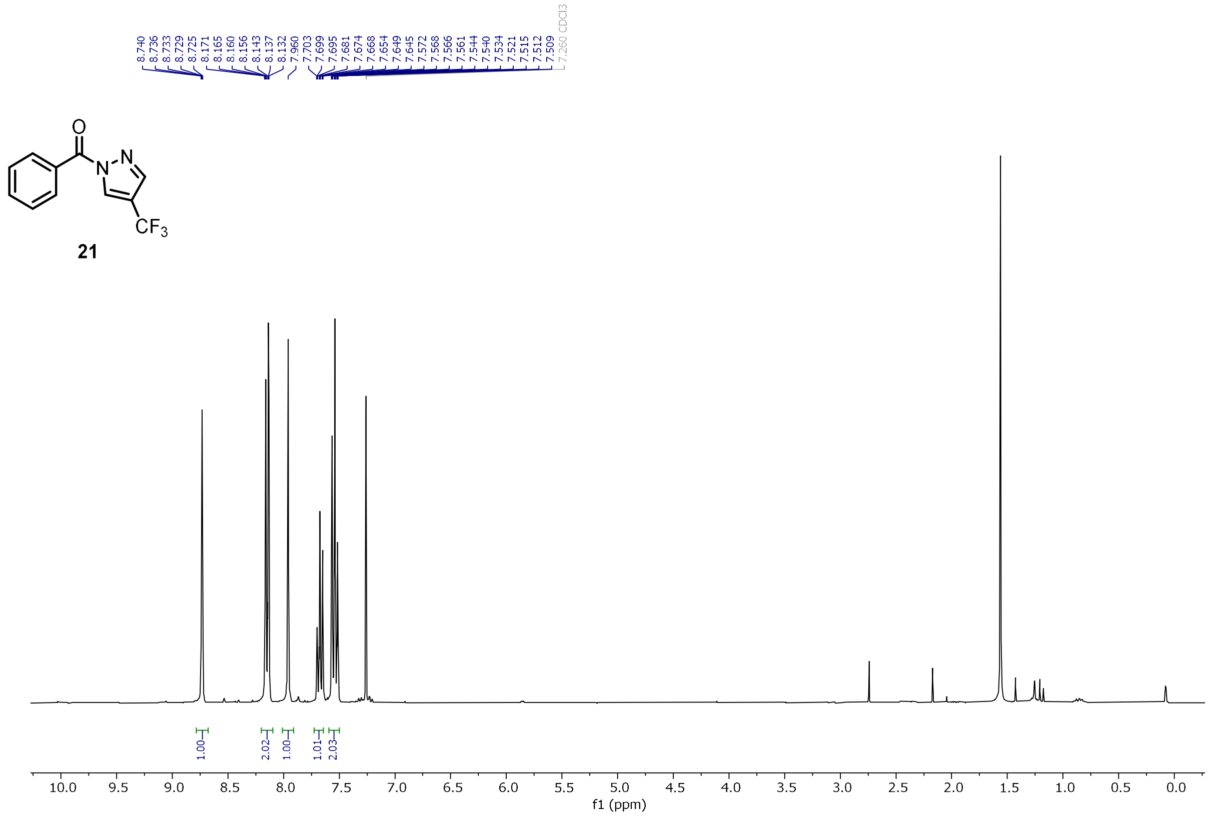

^{13}C NMR (101 MHz, Chloroform-*d*)

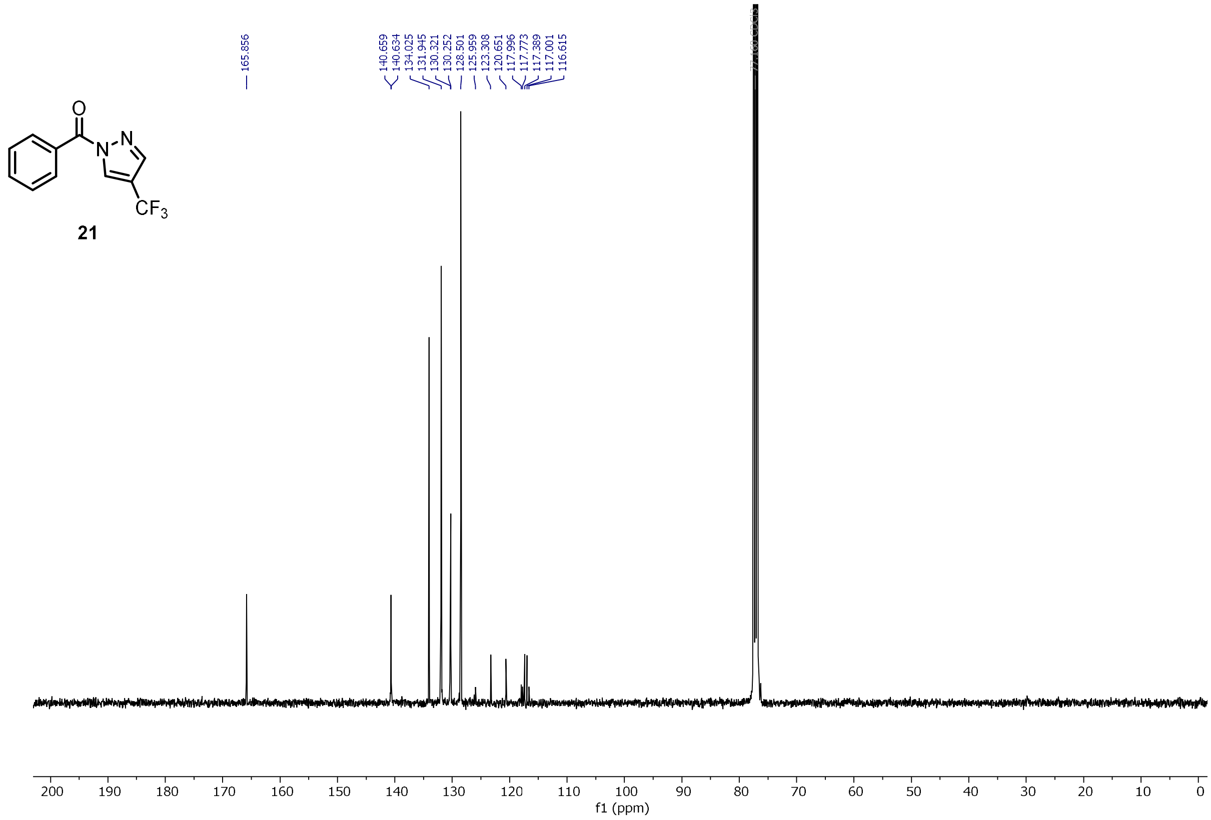

^{19}F NMR (282 MHz, Chloroform-*d*)

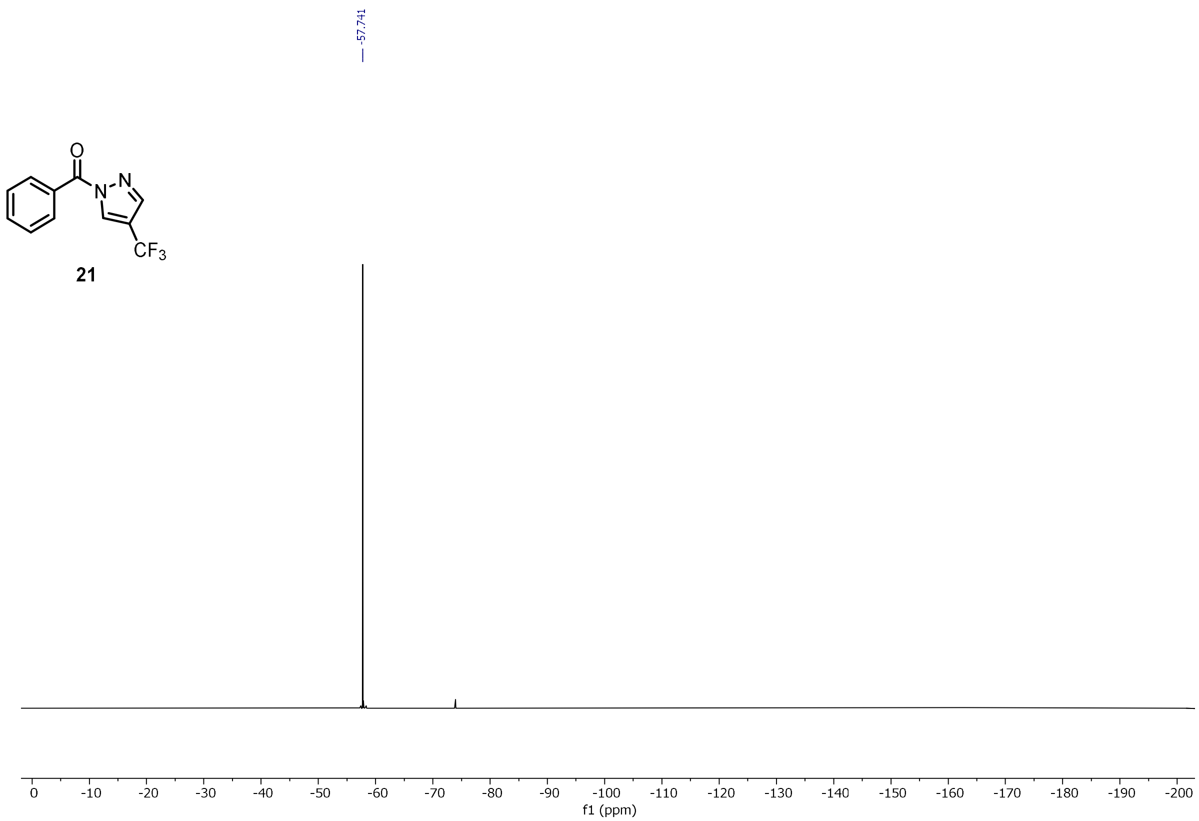

^{1}H NMR (300 MHz, Chloroform-*d*)

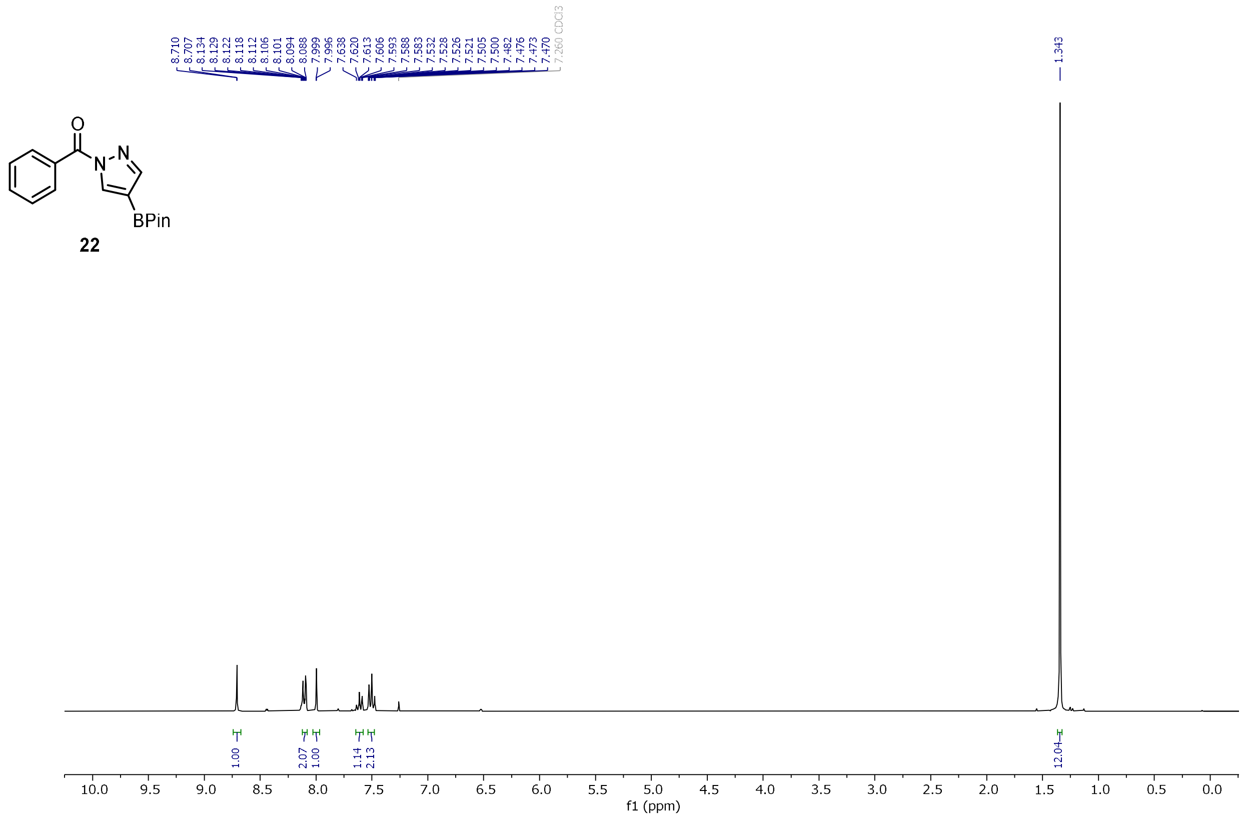

^{11}B NMR (96 MHz, Chloroform-*d*)

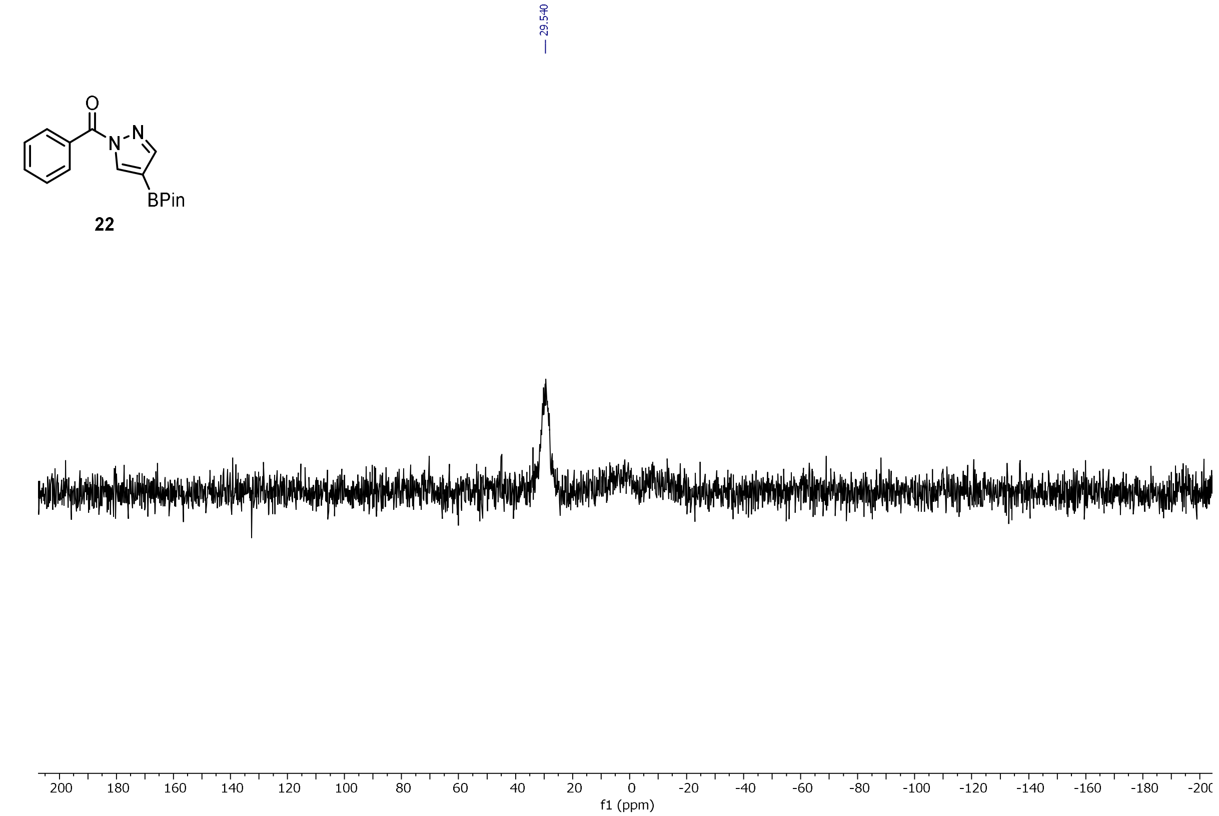

^{13}C NMR (75 MHz, Chloroform-*d*)

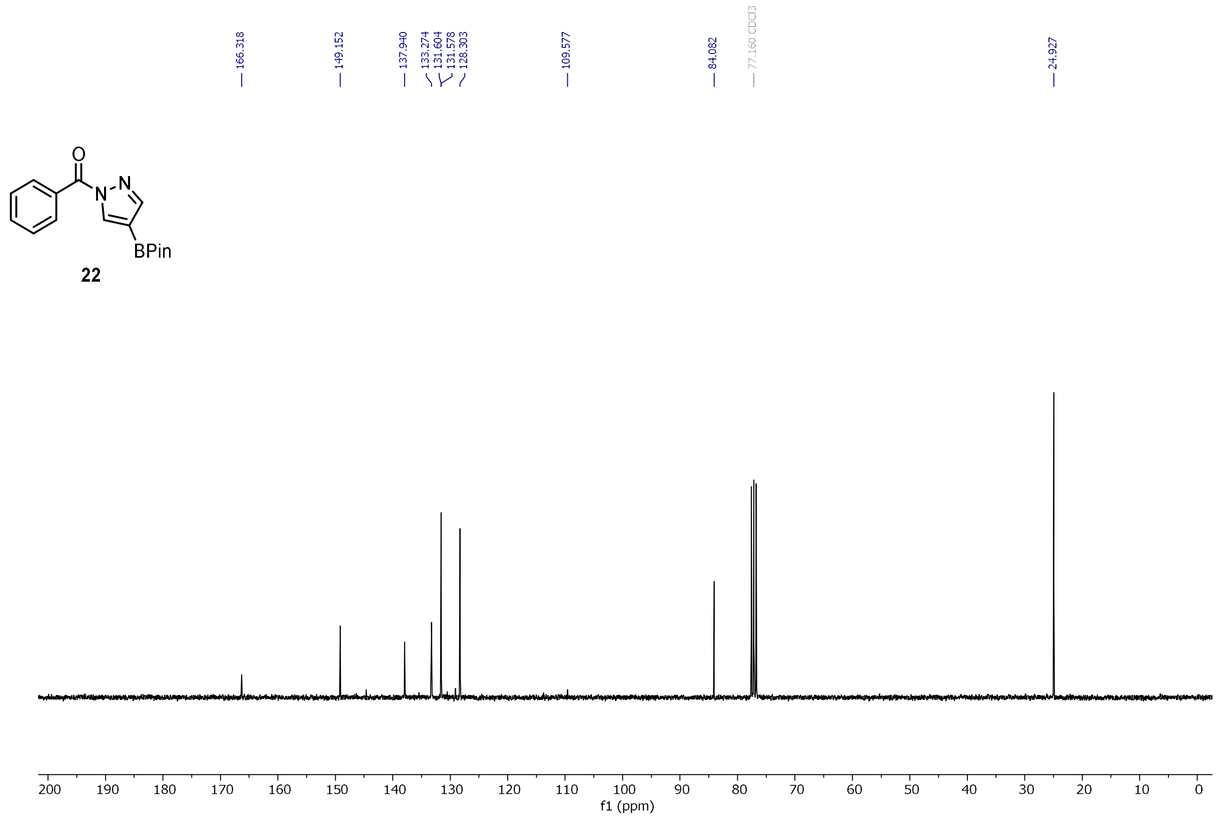

^{1}H NMR (300 MHz, Chloroform-*d*)

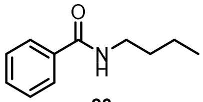

23

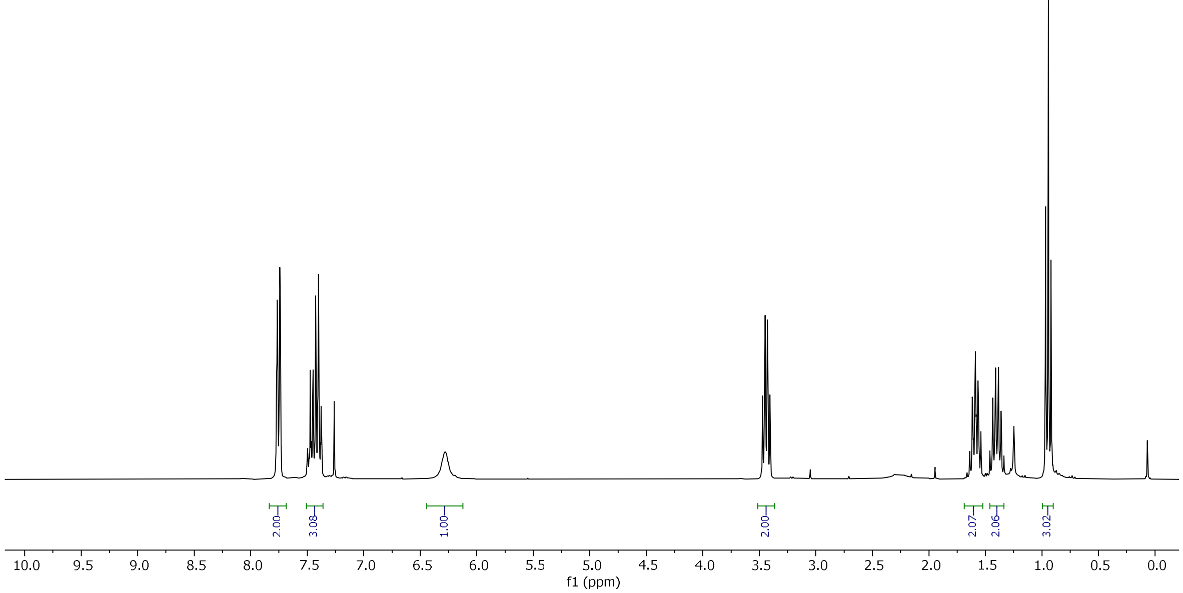

^{13}C NMR (75 MHz, Chloroform-*d*)

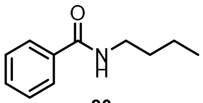

23

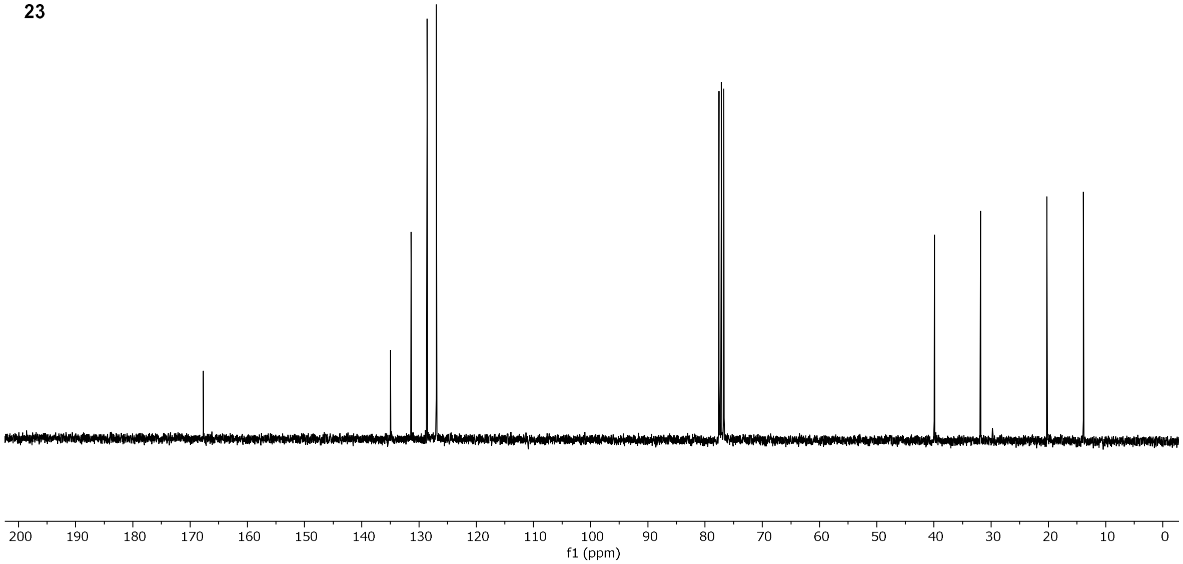

¹H NMR (300 MHz, Chloroform-*d*)

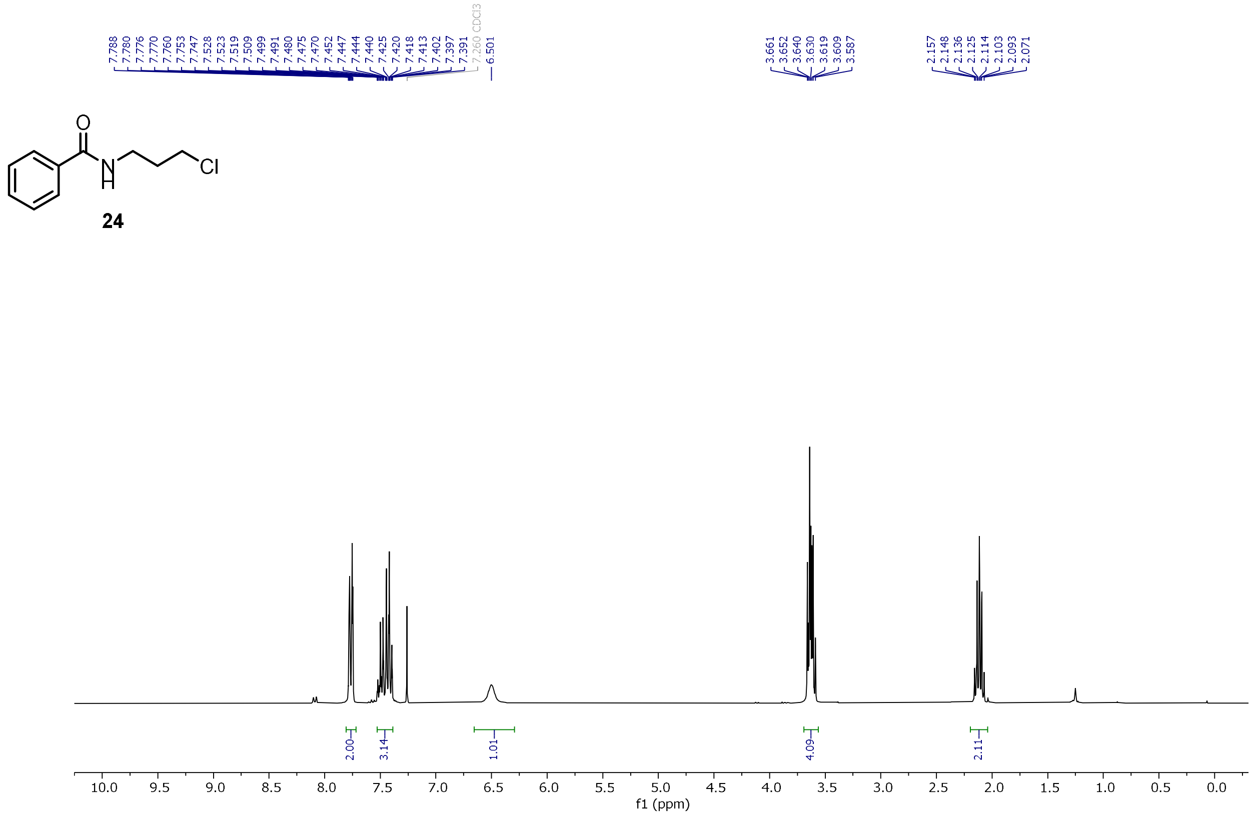

¹³C NMR (75 MHz, Chloroform-*d*)

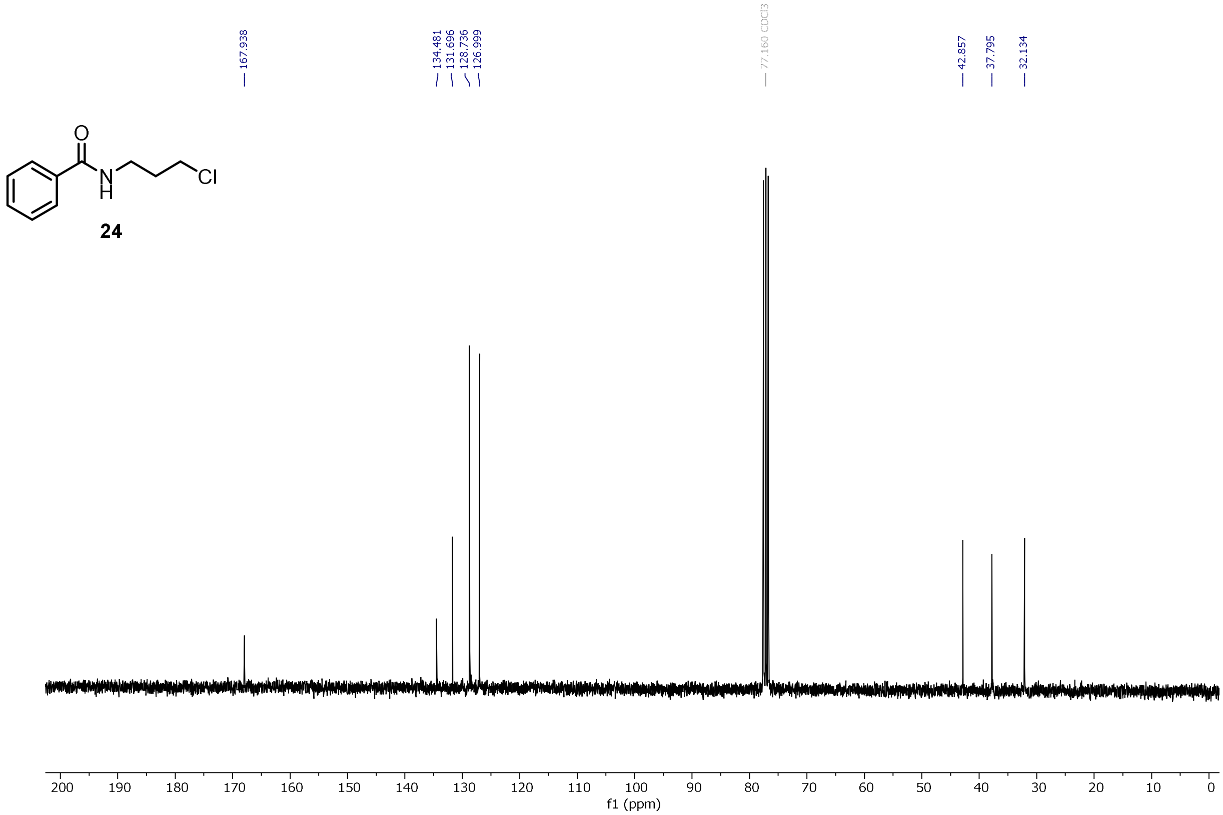

^{1}H NMR (300 MHz, Chloroform-*d*)

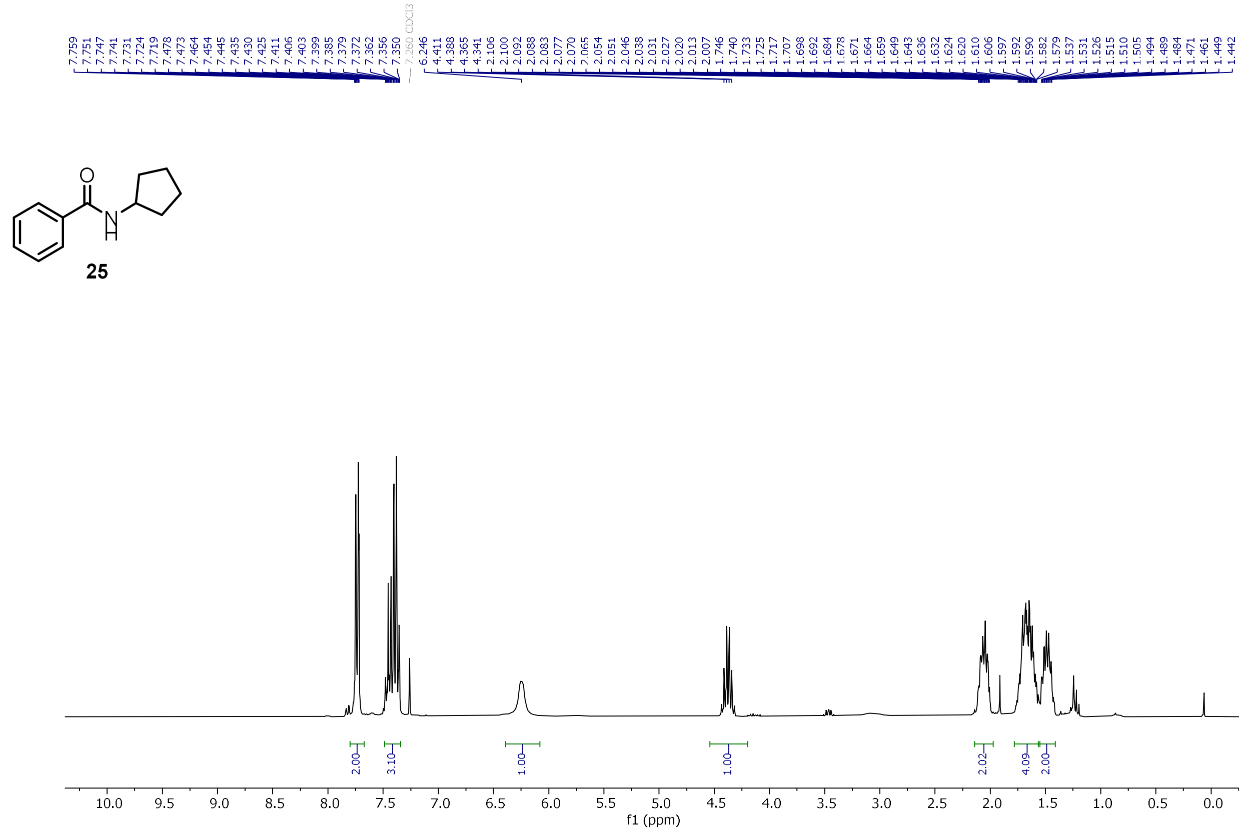

^{13}C NMR (75 MHz, Chloroform-*d*)

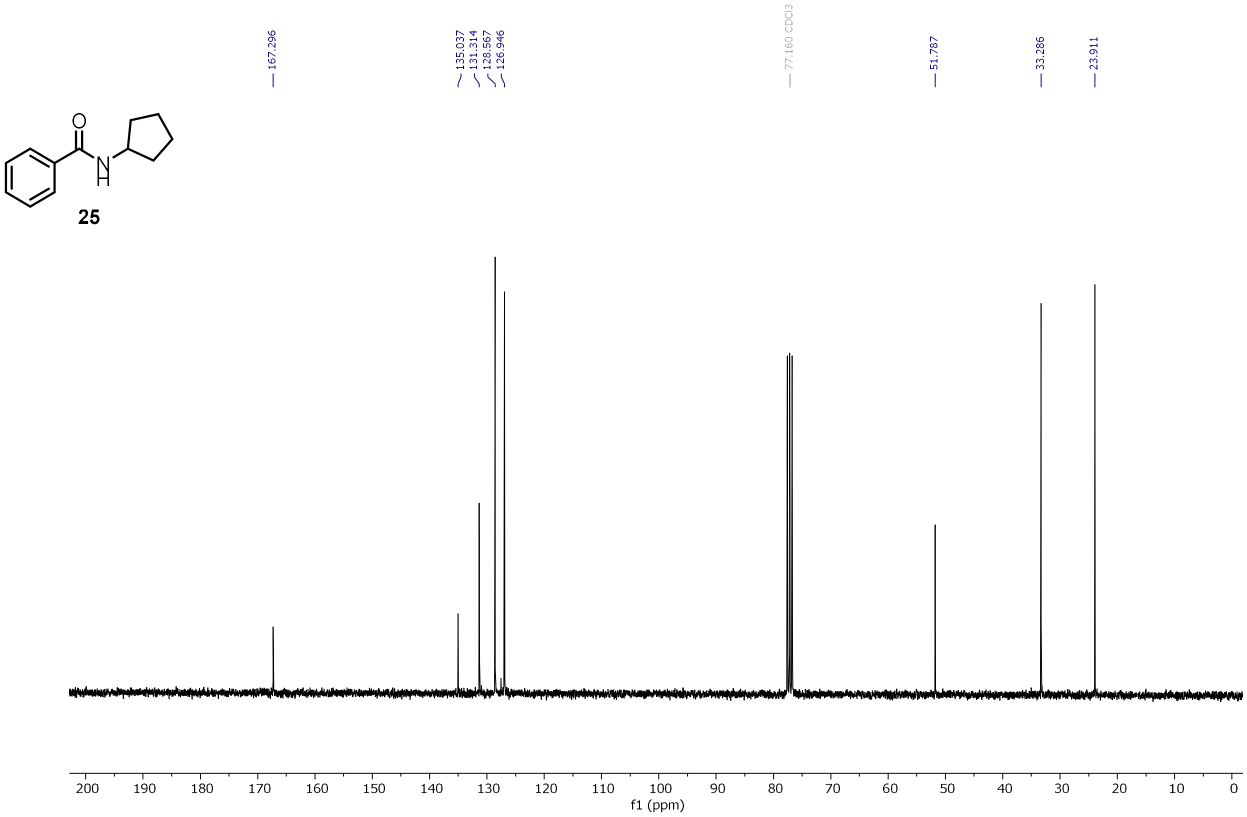

^{1}H NMR (300 MHz, Chloroform-*d*)

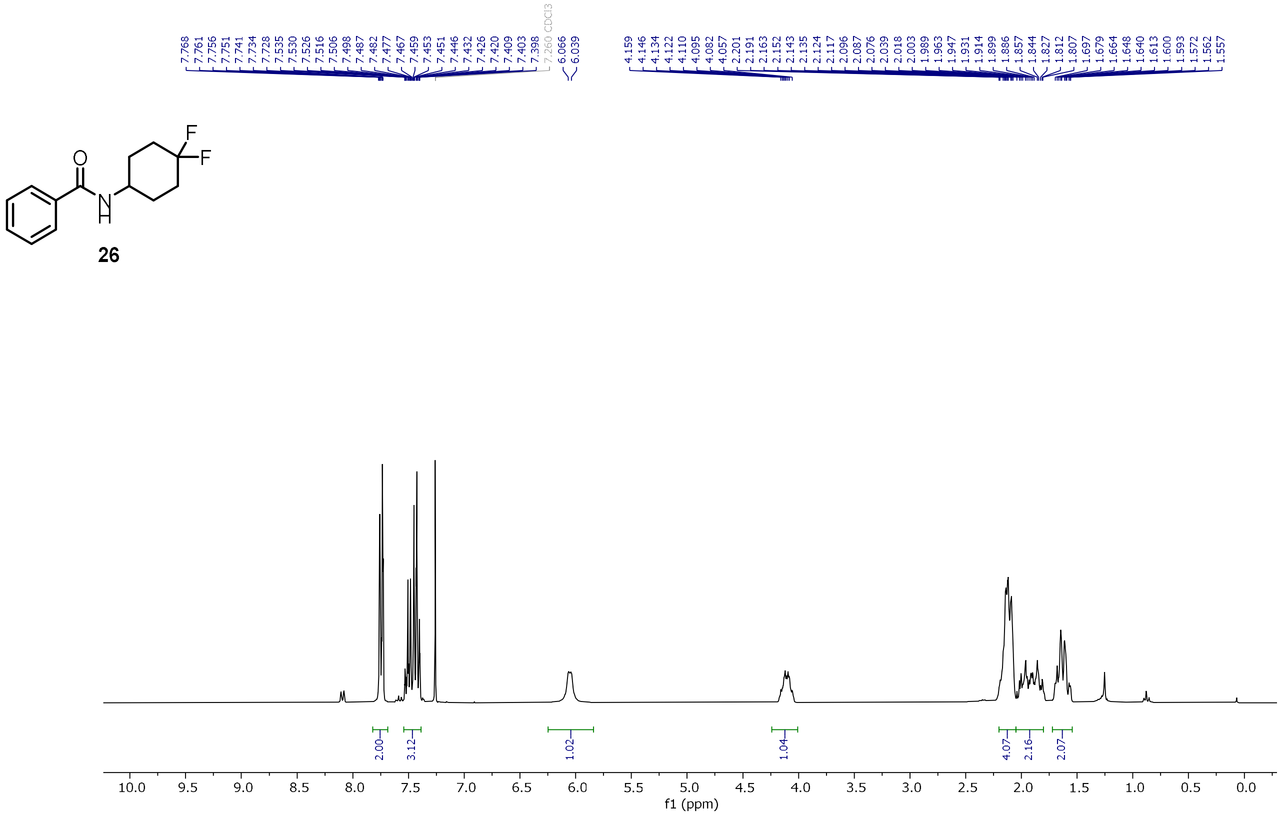

^{13}C NMR (75 MHz, Chloroform-*d*)

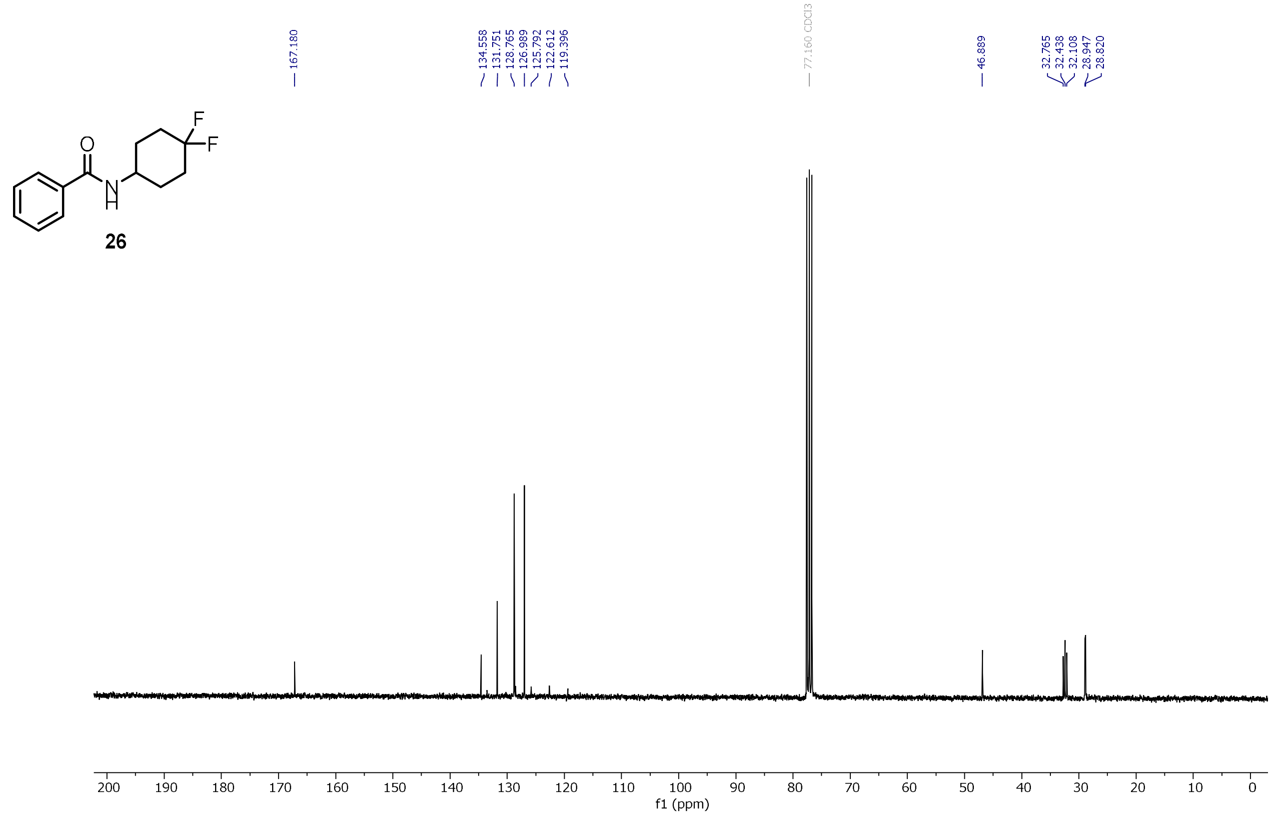

^{19}F NMR (282 MHz, Chloroform-*d*)

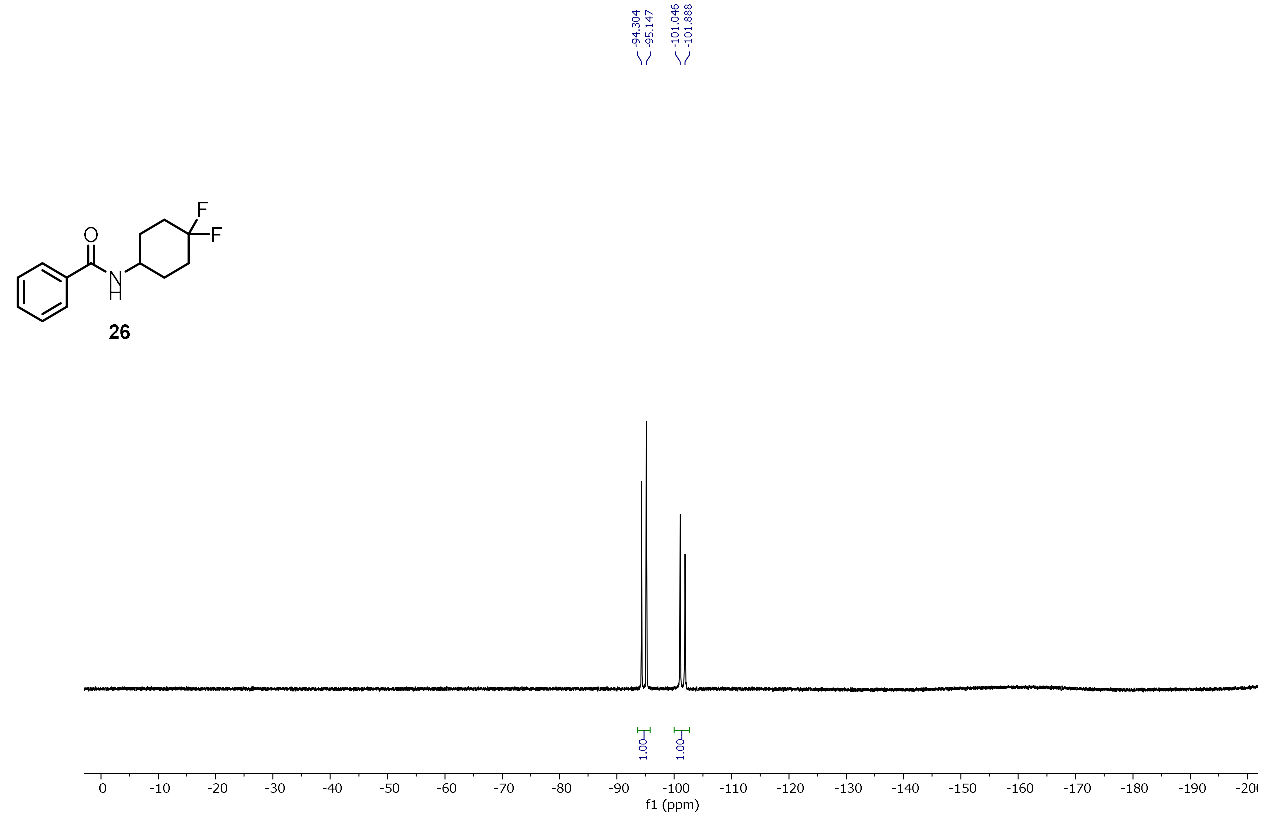

^{1}H NMR (300 MHz, Chloroform-*d*)

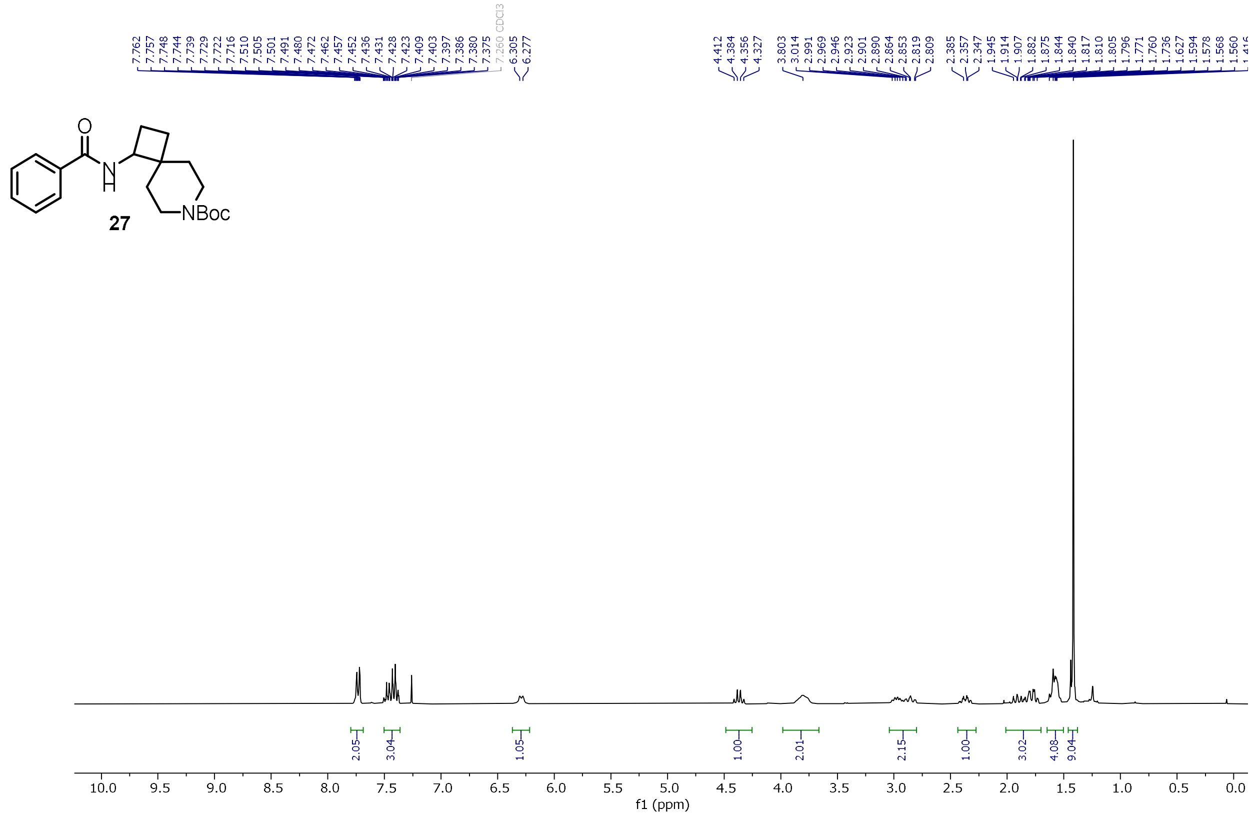

^{13}C NMR (101 MHz, Chloroform-*d*)

27

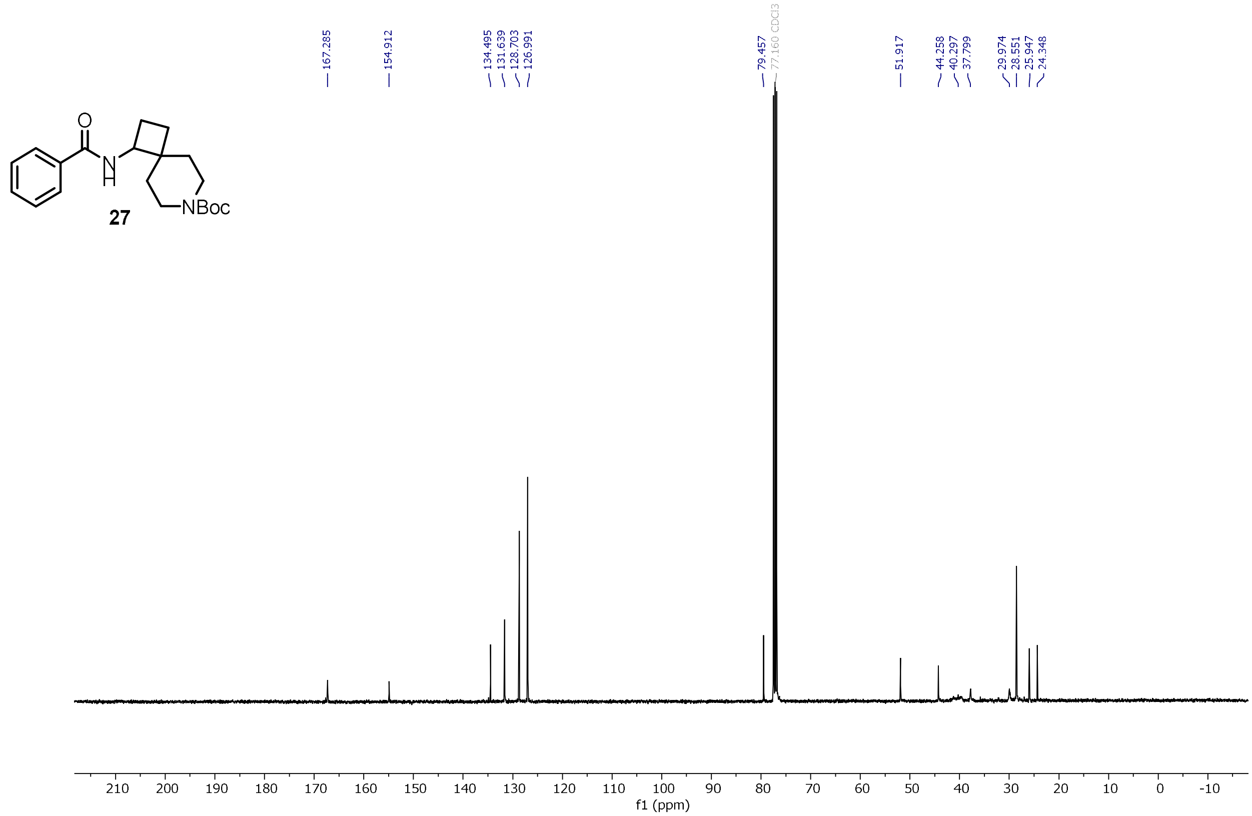

^{1}H NMR (300 MHz, Chloroform-*d*)

28

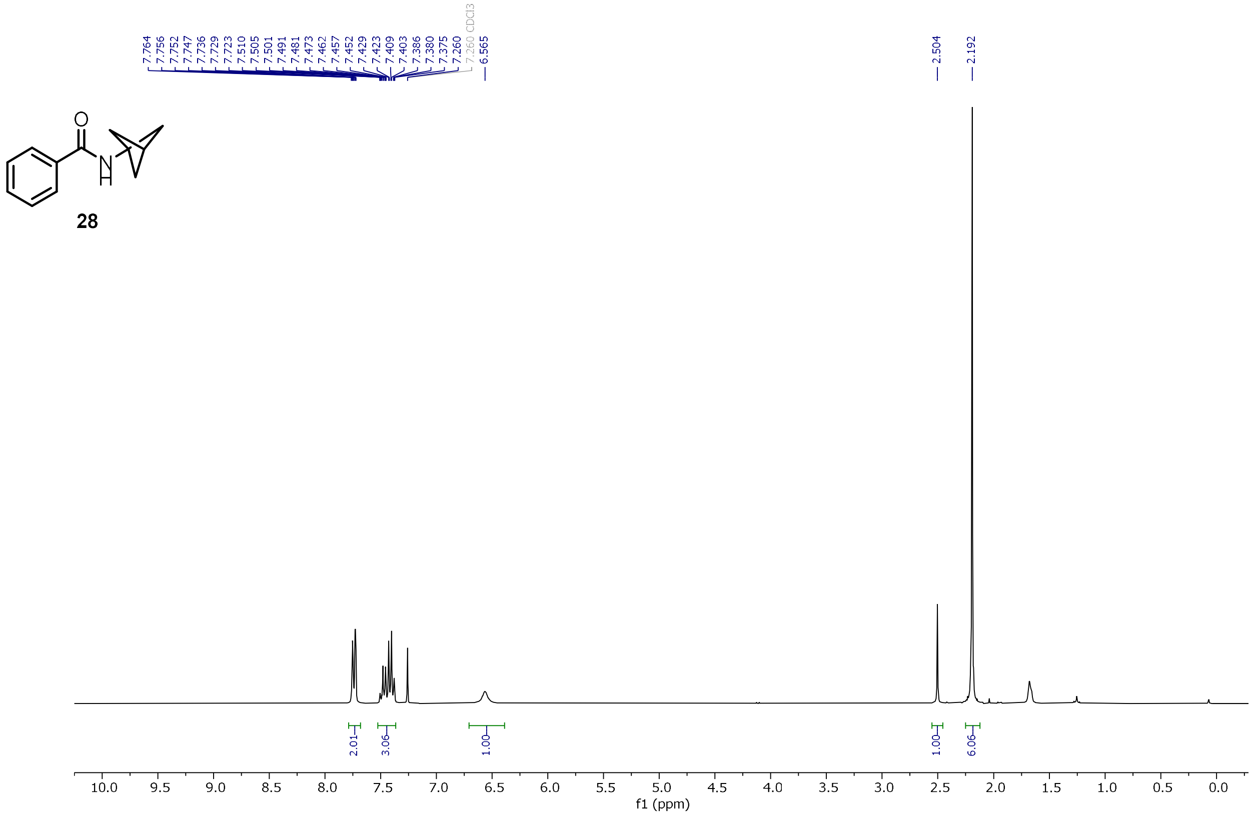

^{13}C NMR (75 MHz, Chloroform-*d*)

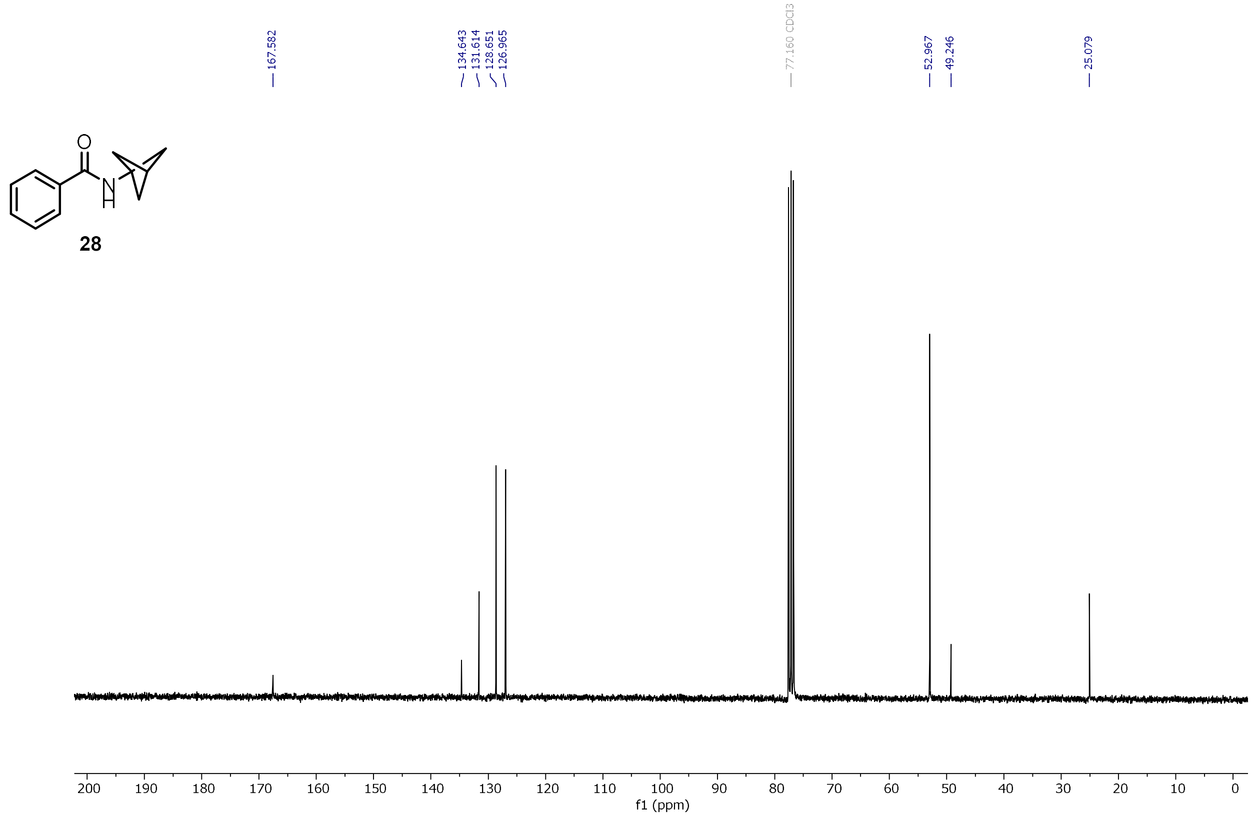

^{1}H NMR (300 MHz, Chloroform-*d*)

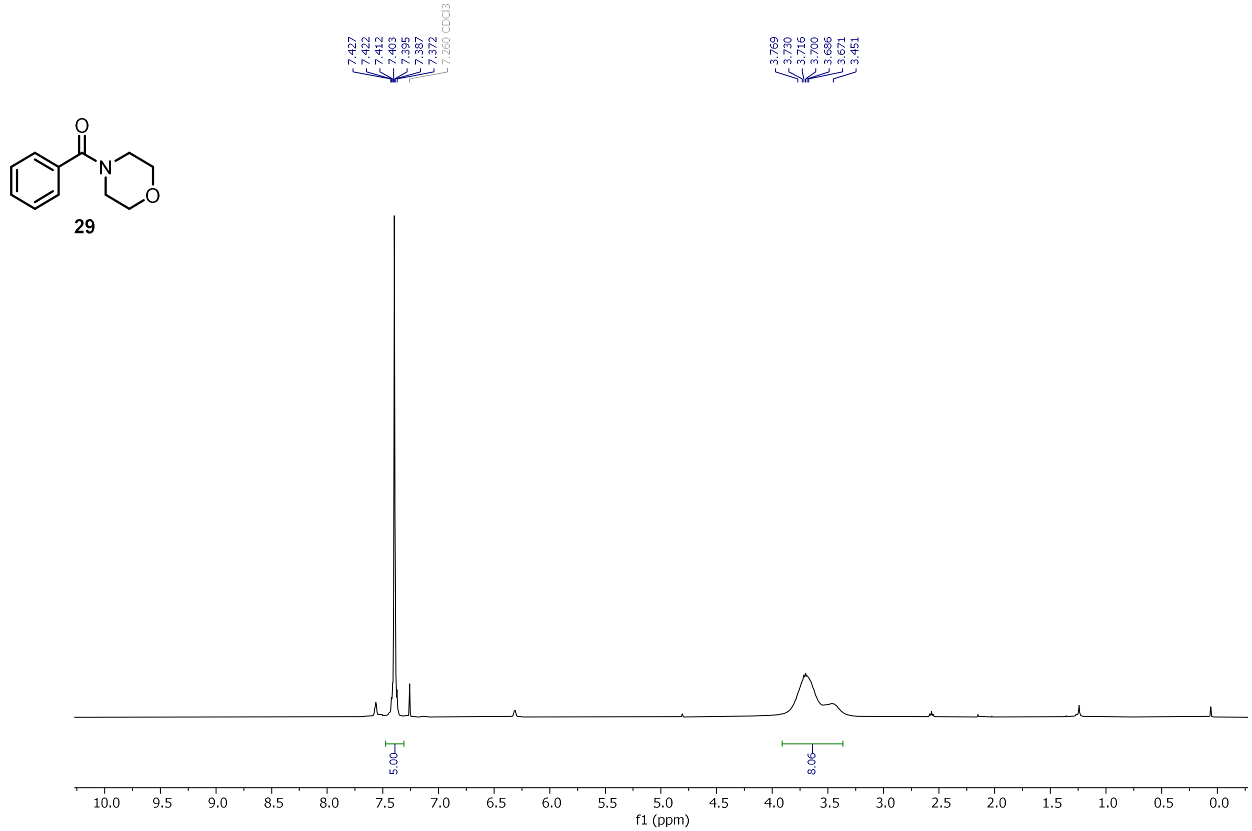

^{13}C NMR (75 MHz, Chloroform-*d*)

29

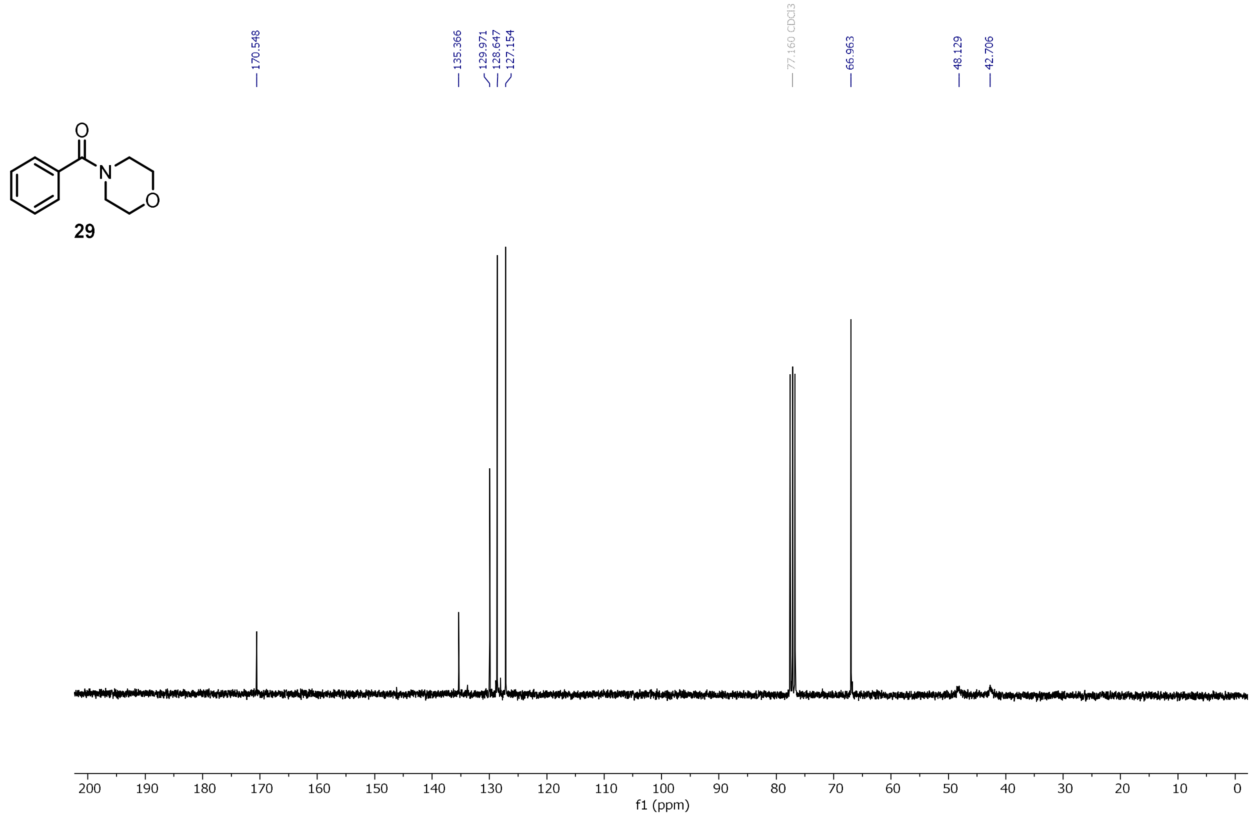

^{1}H NMR (300 MHz, Chloroform-*d*)

30

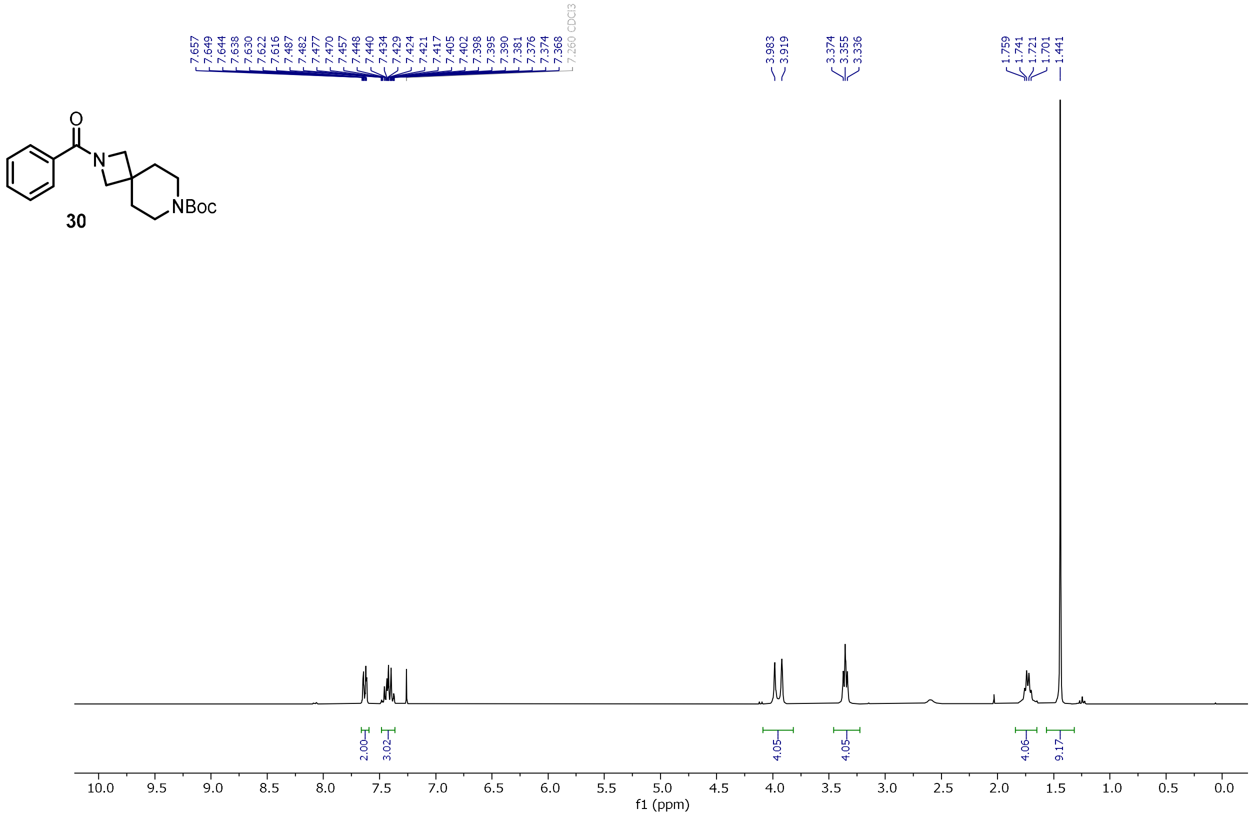

^{13}C NMR (101 MHz, Chloroform-*d*)

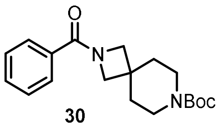

30

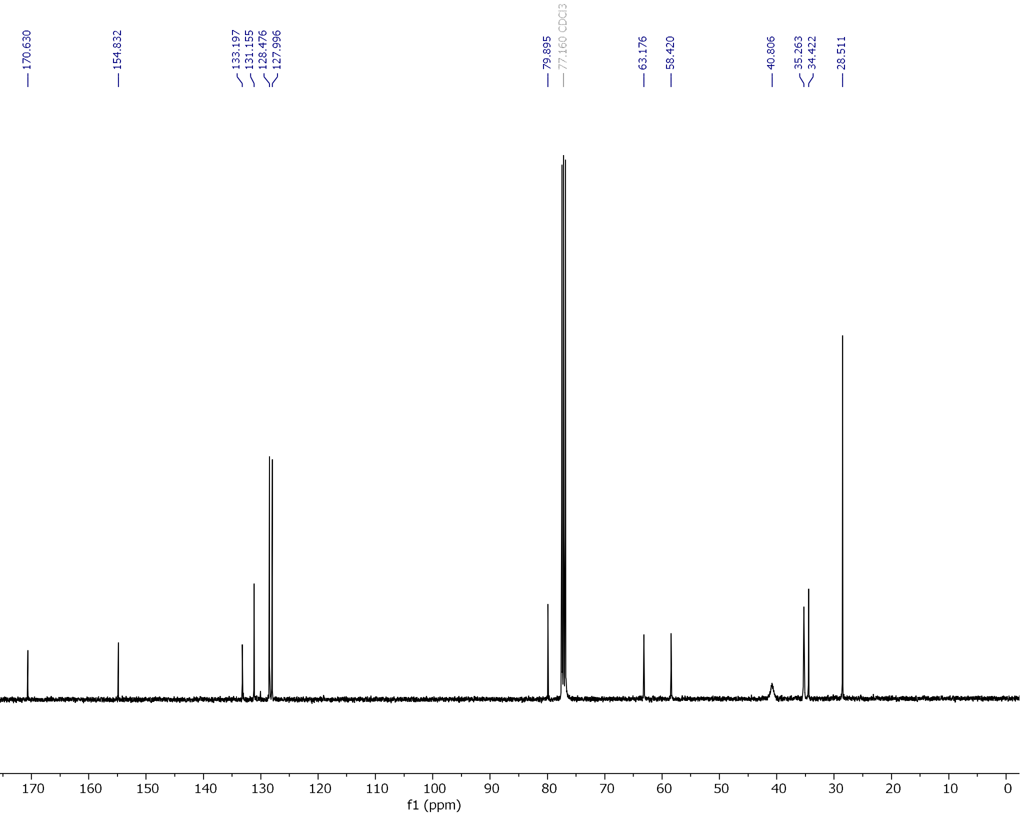

^{1}H NMR (400 MHz, Chloroform-*d*)

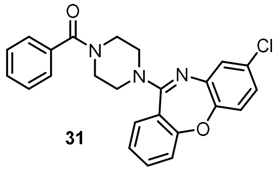

31

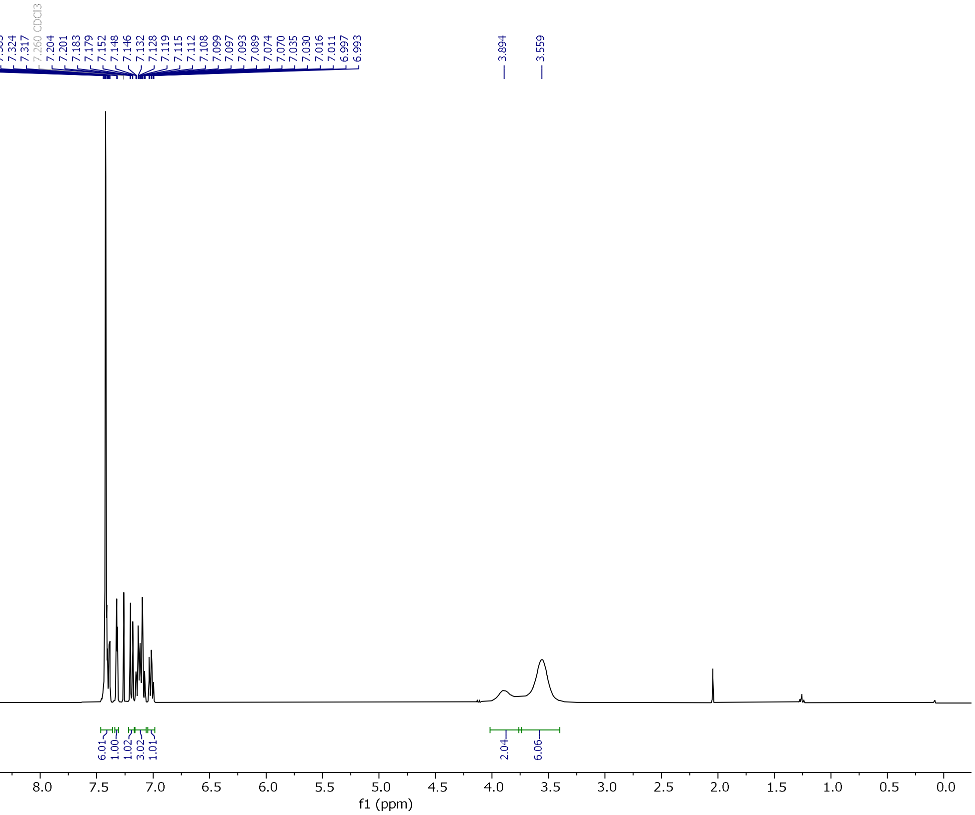

^{13}C NMR (101 MHz, Chloroform-*d*)

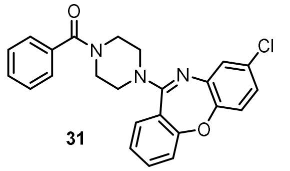

31

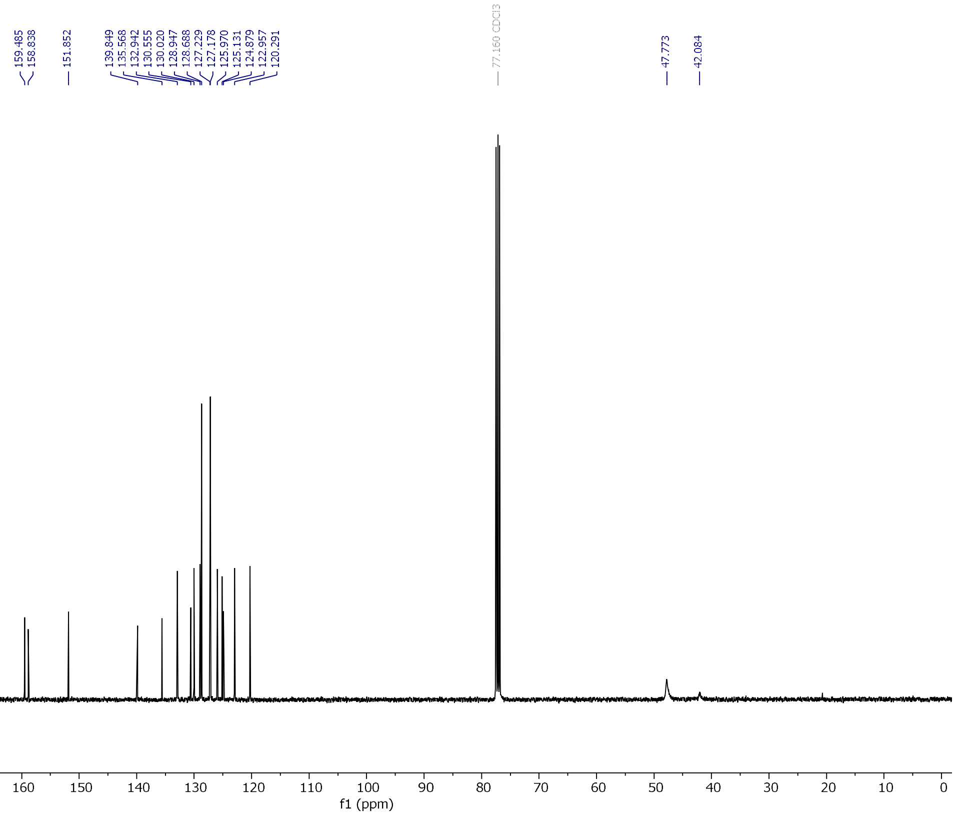

^{1}H NMR (300 MHz, Chloroform-*d*)

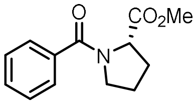

32

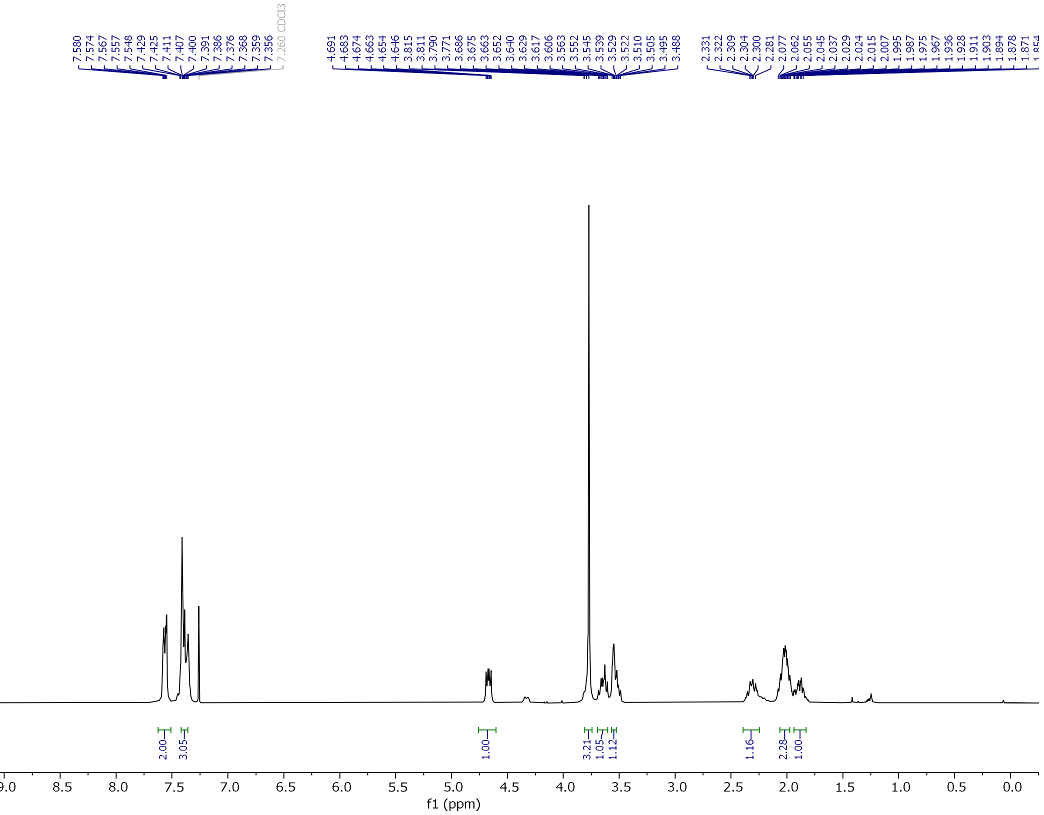

^{13}C NMR (75 MHz, Chloroform-*d*)

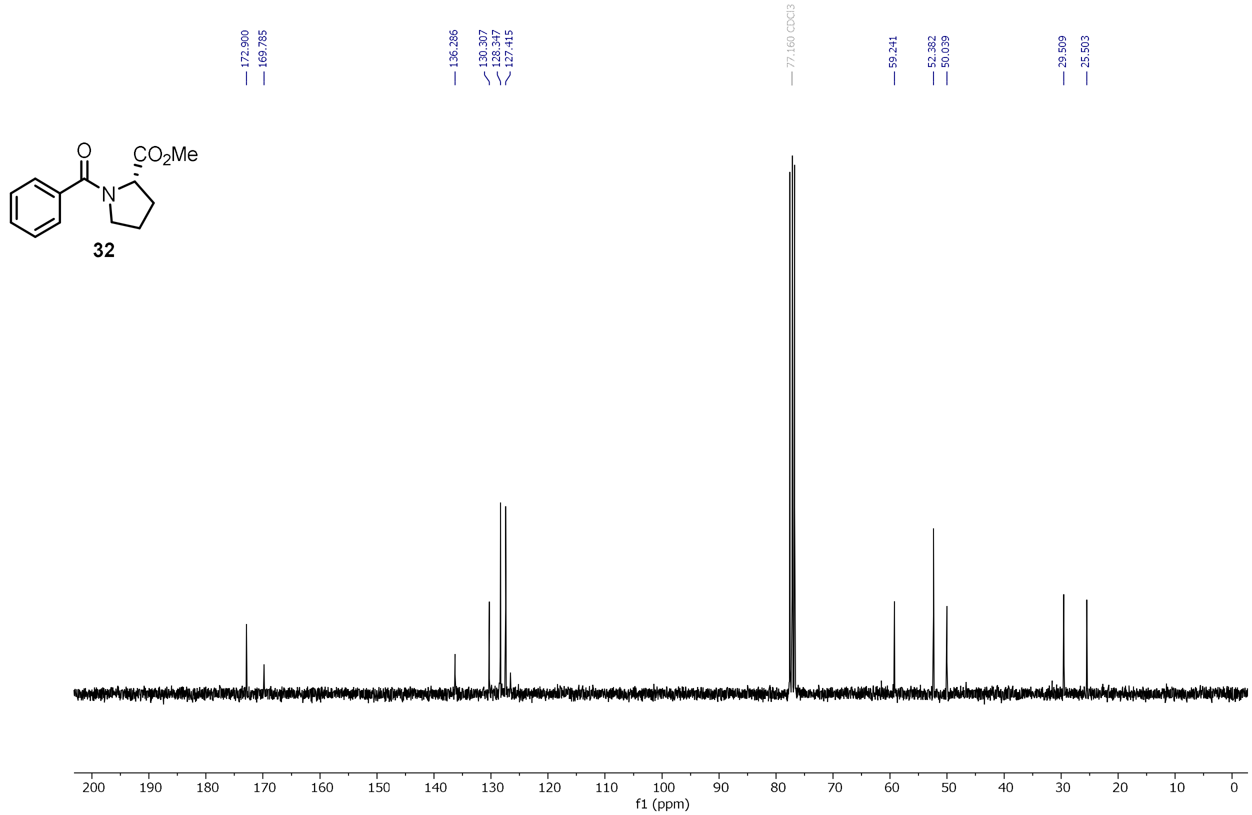

32

^{1}H NMR (400 MHz, Chloroform-*d*)

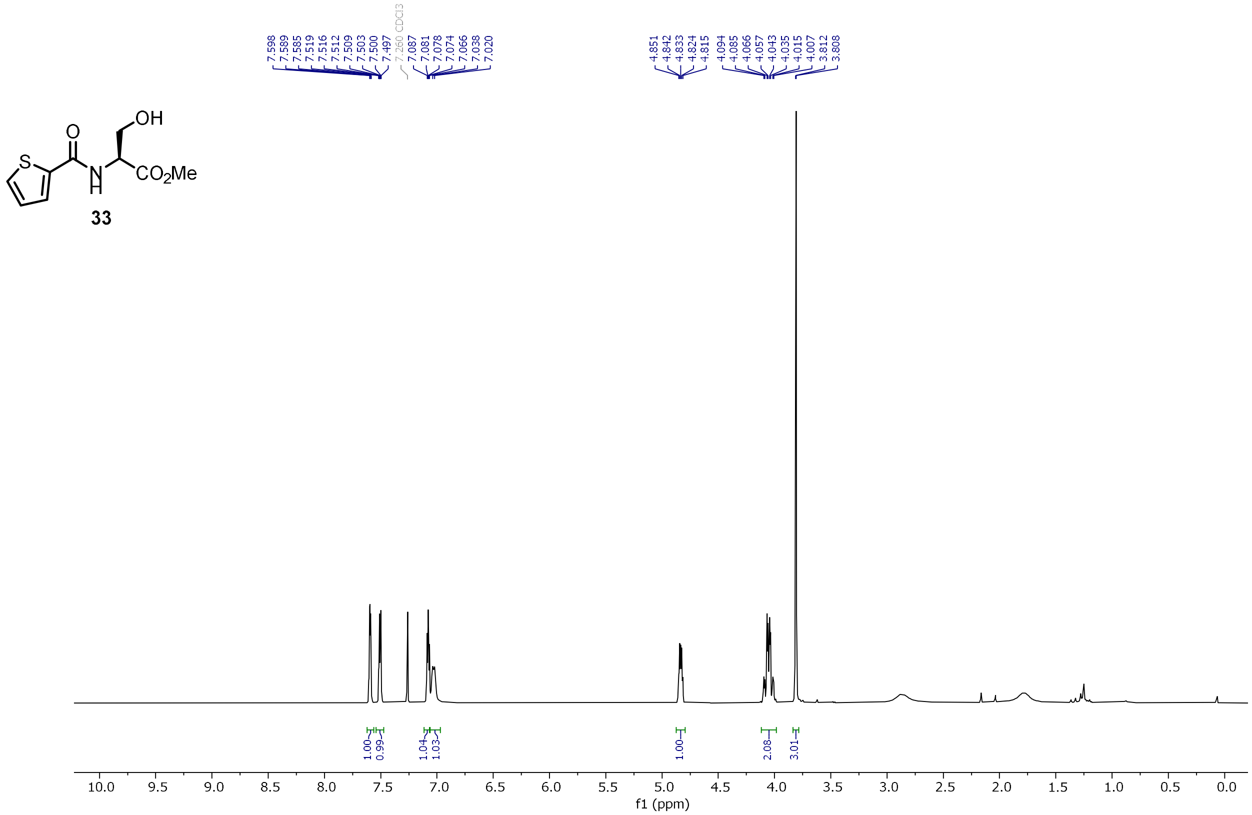

33

^{13}C NMR (101 MHz, Chloroform-*d*)

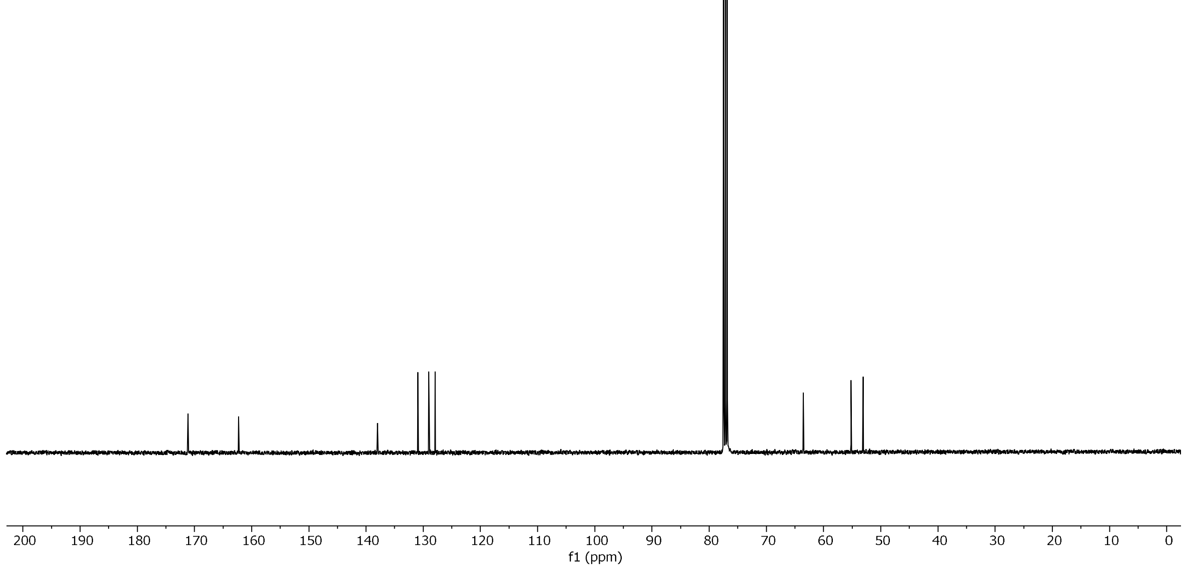

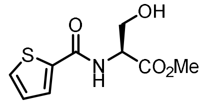

33

^{1}H NMR (400 MHz, Chloroform-*d*)

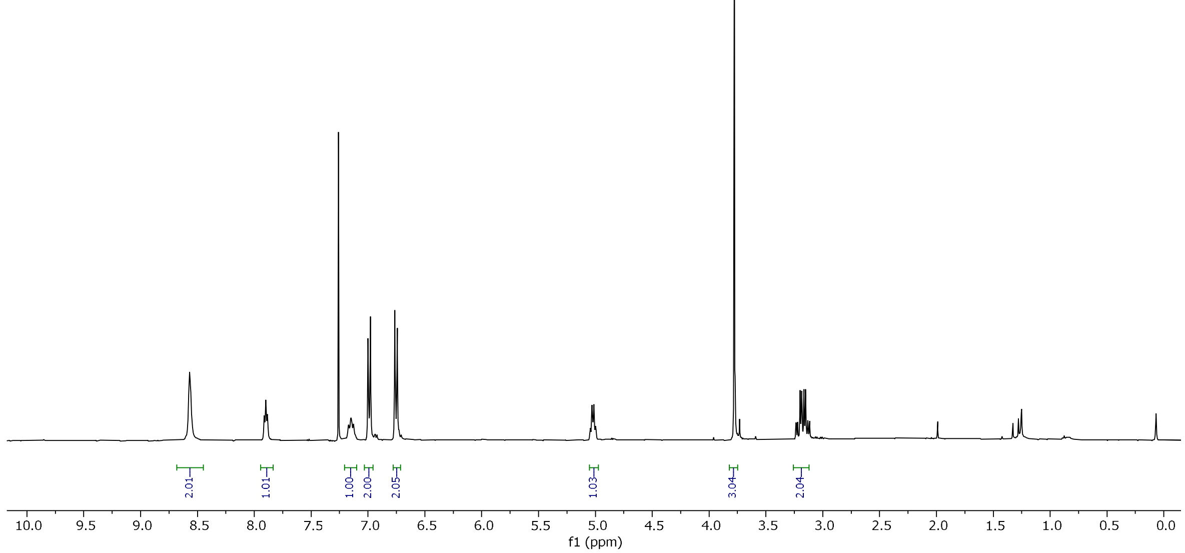

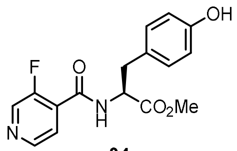

34

^{13}C NMR (75 MHz, Chloroform-*d*)

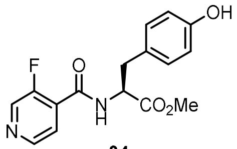

34

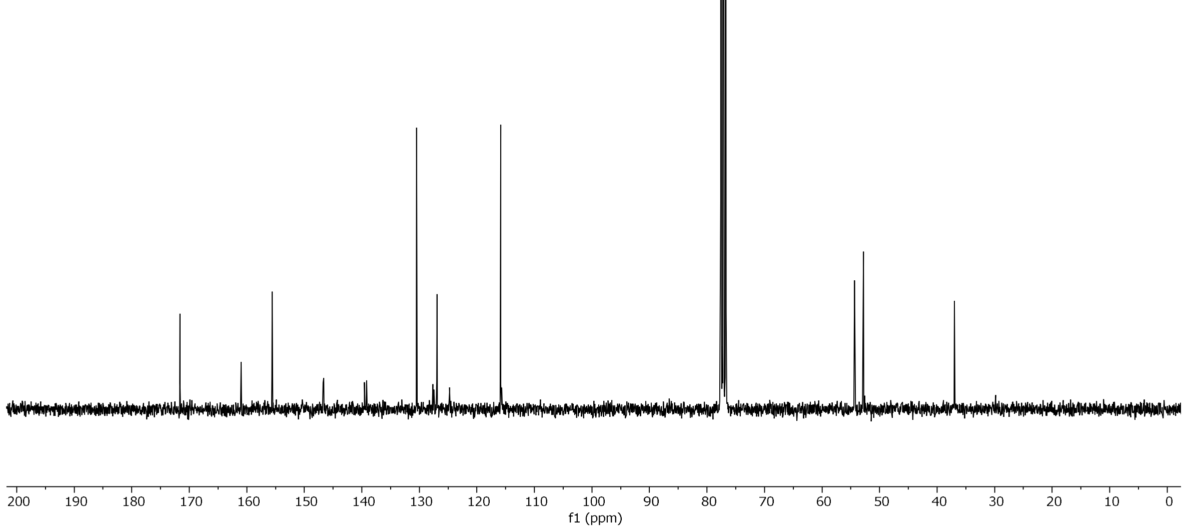

^{19}F NMR (282 MHz, Chloroform-*d*)

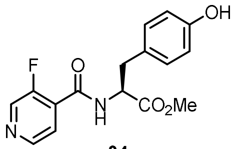

34

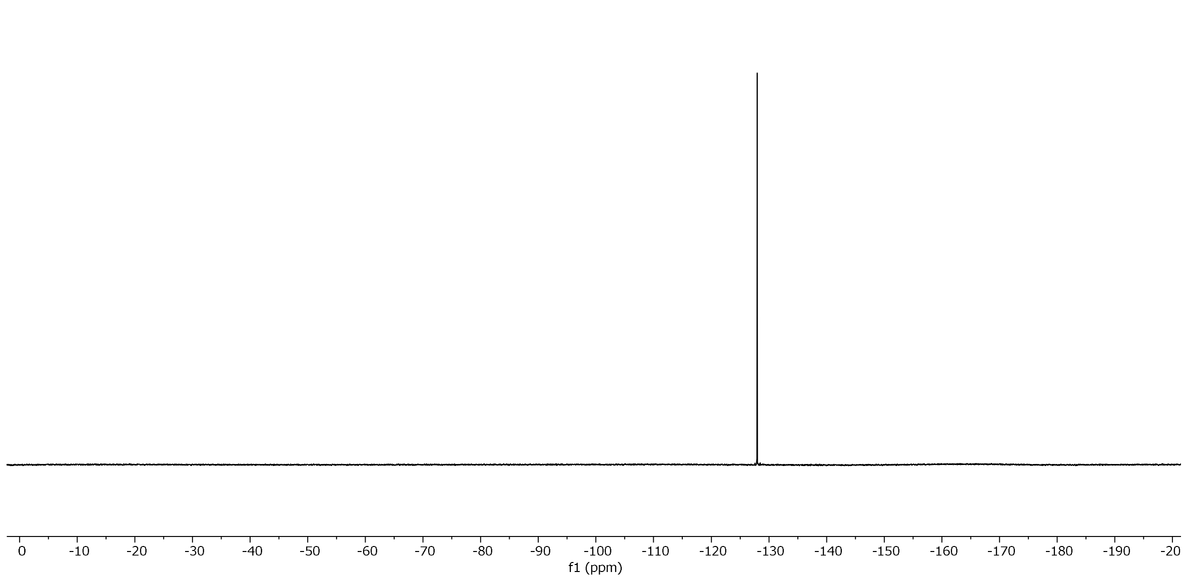

^{1}H NMR (300 MHz, Chloroform-*d*)

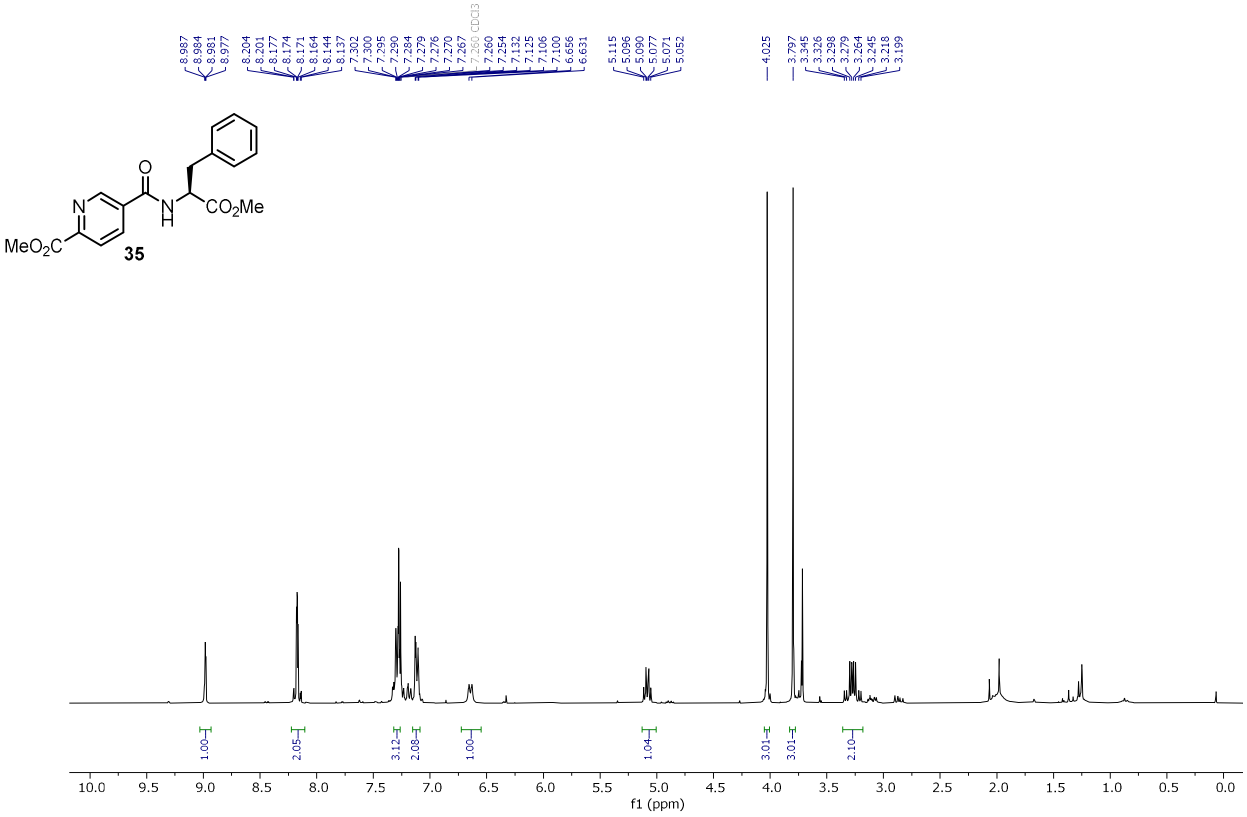

^{13}C NMR (75 MHz, Chloroform-*d*)

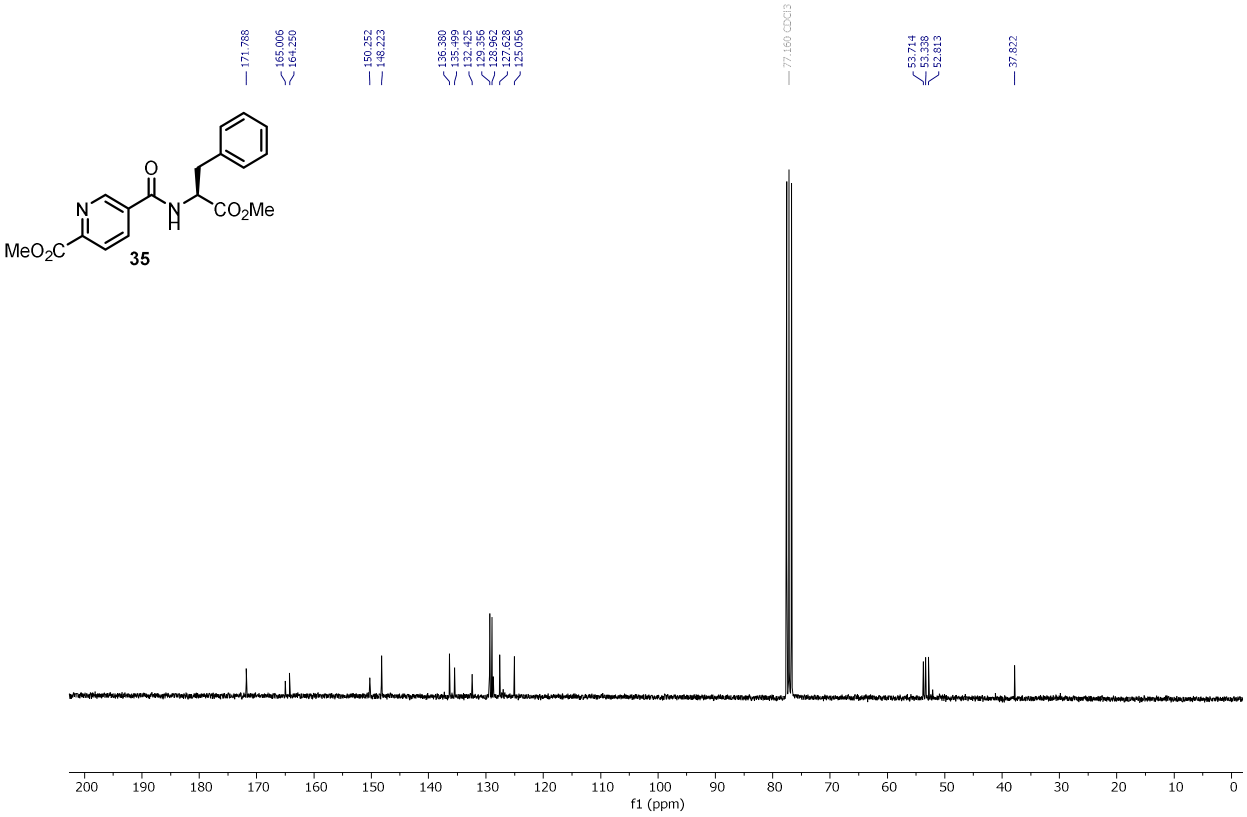

^{1}H NMR (300 MHz, Chloroform-*d*)

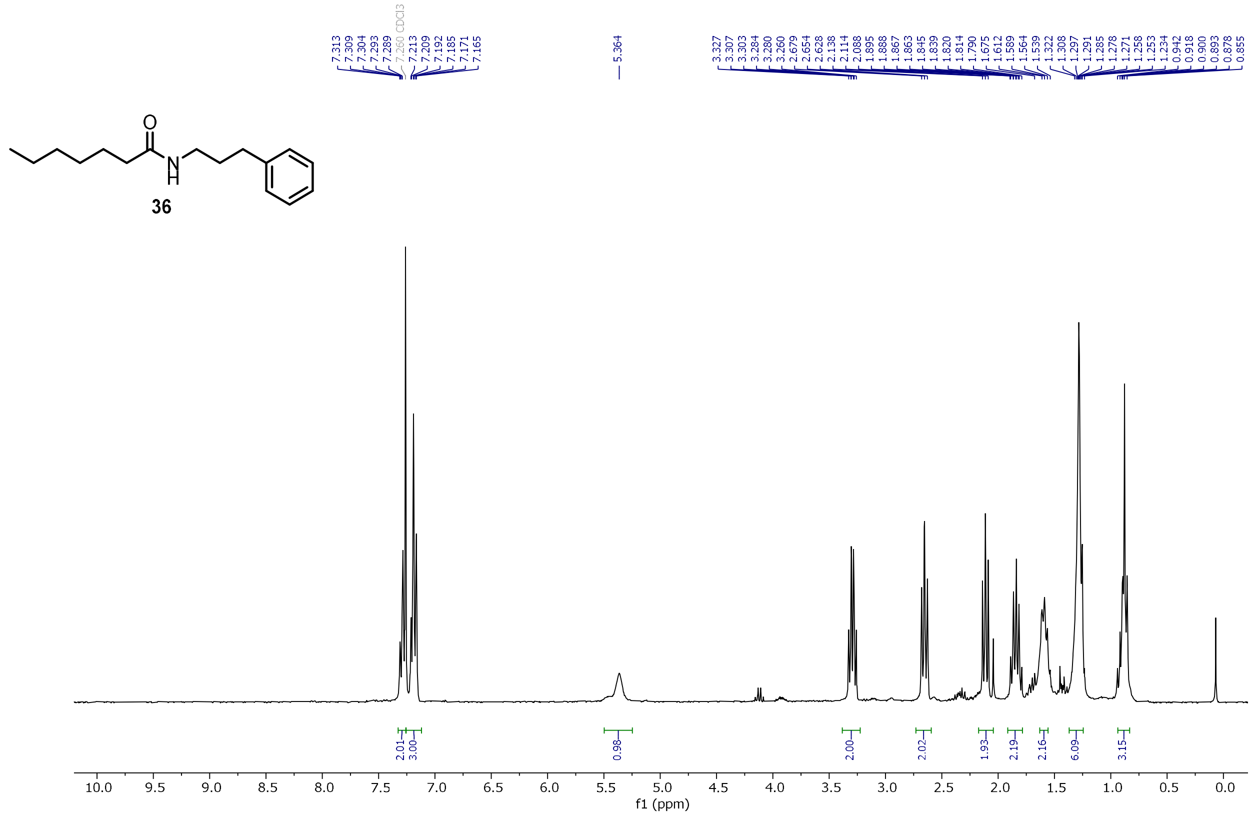

^{13}C NMR (101 MHz, Chloroform-*d*)

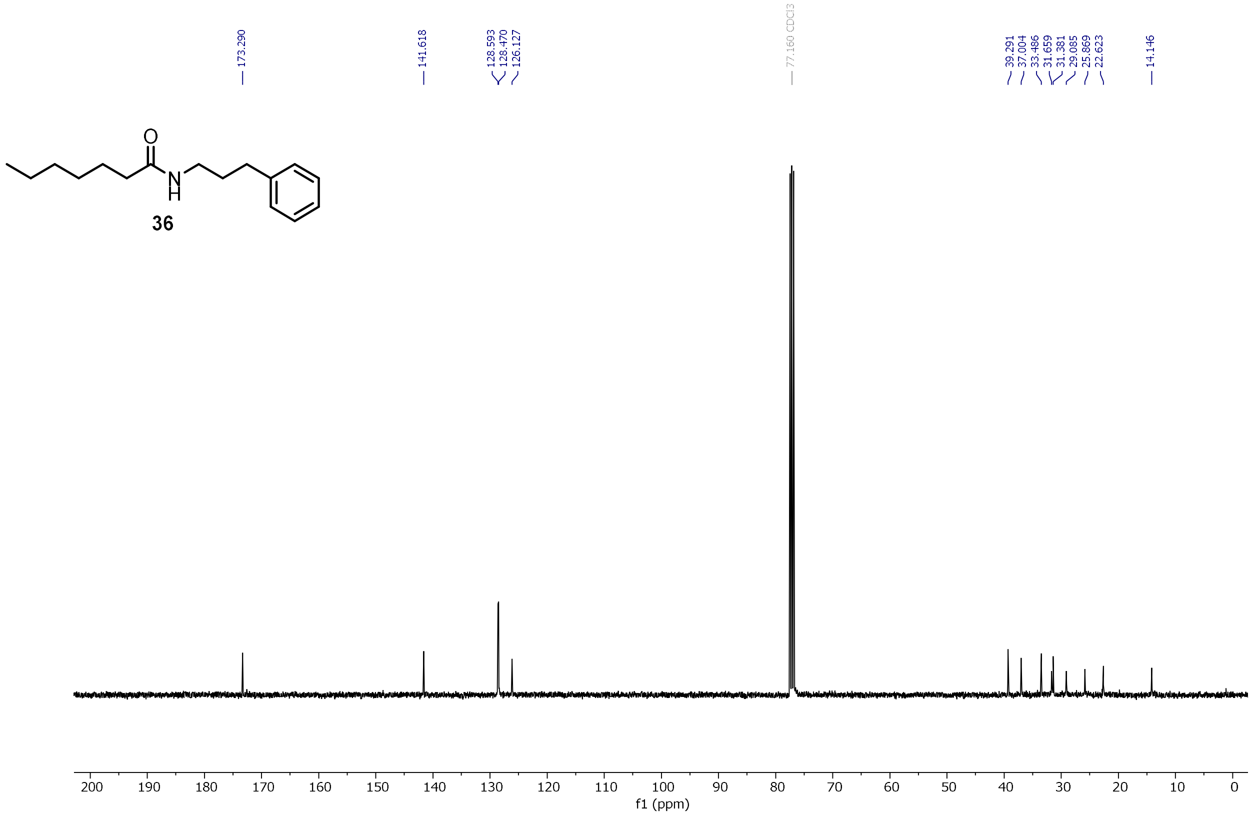

^{1}H NMR (300 MHz, Chloroform-*d*)

37

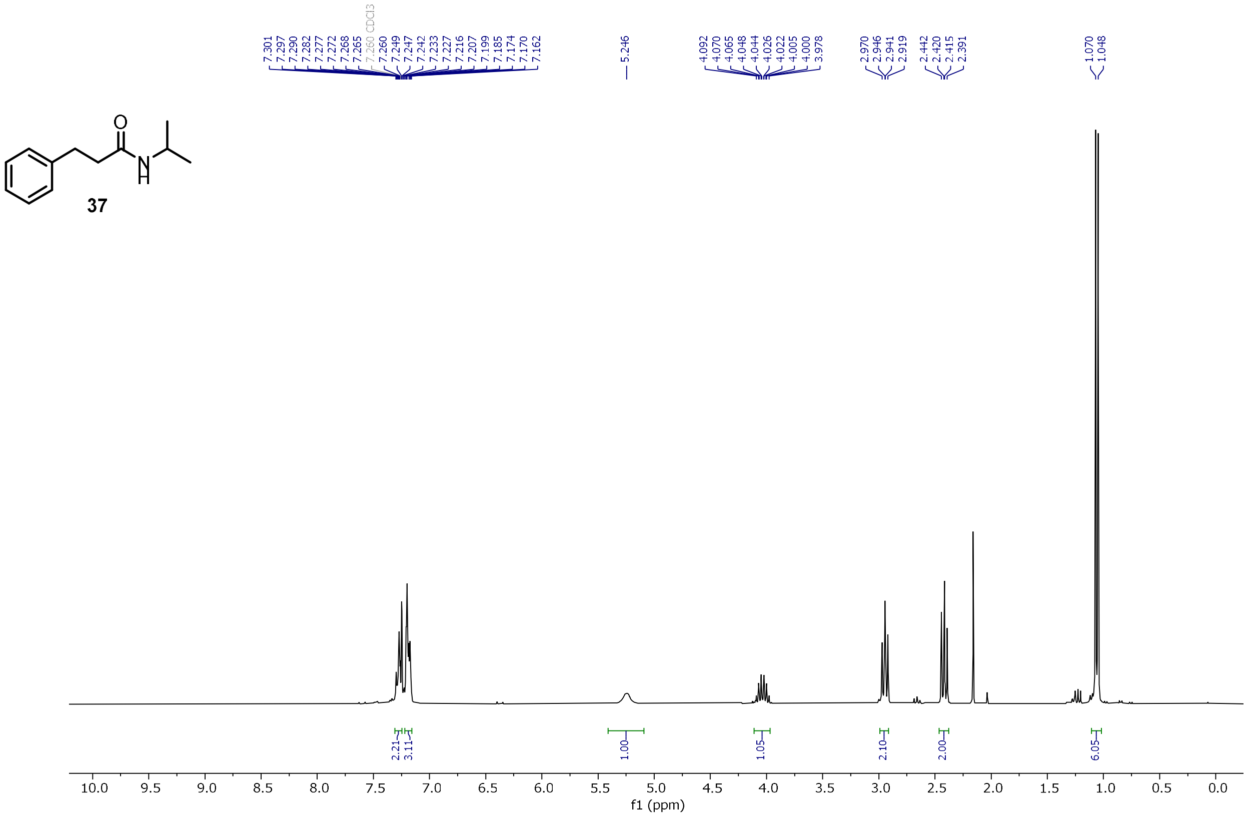

^{13}C NMR (101 MHz, Chloroform-*d*)

37

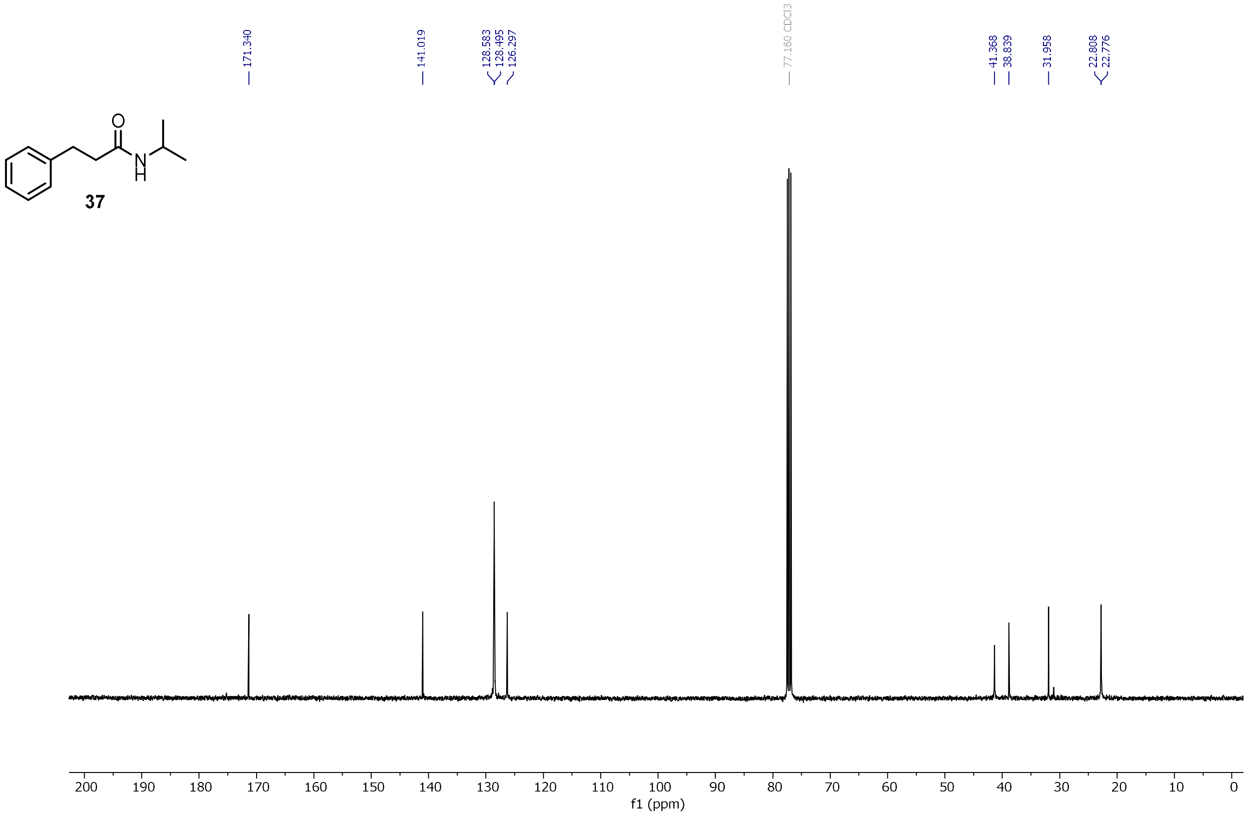

Supplement: Supplementary file 1 — Supporting Information [file CHEM-31-e02237-s001.pdf]
